# Chemical Constituents from Roots of *Sophora davidii* (Franch.) Skeels and Their Glucose Transporter 4 Translocation Activities

**DOI:** 10.3390/molecules26030756

**Published:** 2021-02-01

**Authors:** Kangdi Li, Yuanren Ma, Tongxi Zhou, Xinzhou Yang, Ho-Young Choi

**Affiliations:** 1 School of Pharmaceutical Sciences, South-Central University for Nationalities, Wuhan 430074, China; kdli@whu.edu.cn (K.L.); 13007136998@163.com (Y.M.); tc13627123095@163.com (T.Z.); 2 College of Korean Medicine, Kyung Hee University, Seoul 02447, Korea

**Keywords:** *Sophora davidii* (Franch.) Skeels, stilbene oligomers, isoflavan, arylbenzofuran, GLUT-4, ECD

## Abstract

*Sophora davidii* (Franch.) Skeels is a multi-purpose traditional medicine that has long been used for the treatment of various diseases. To discover the potential bioactive composition of *S. davidii*, a chemical investigation was thus performed. In this research, two new stilbene oligomers, Davidiol E–F (**1**–**2**), one new 4-aryl-substituted isoflavan Davidinin A (**3**), and one new 2-arylbenzofuran dimer, Shandougenine C (**4**), as well as six known compounds (**5**–**10**) were obtained from the ethyl acetate fraction of *Sophora davidii* (Franch.) Skeels. The structures of new compounds were established by extensive 1D and 2D nuclear magnetic resonance (NMR) spectra with mass spectroscopy data. The absolute configuration of **1**–**3** was assigned by comparing its experimental and calculated electronic circular dichroism (ECD) spectra. Compounds **1**–**10** promoted glucose transporter 4 (GLUT-4) translocations by the range of 1.28–2.60 folds, respectively. Compound **9** showed the most potent glucose transporter 4 translocations with 1.60 fold enhancement. The result attained in this study indicated that the separation and characterization of these compounds plays an important role in the research and development of new anti-diabetic drugs and pharmaceutical industry.

## 1. Introduction

Diabetes mellitus (DM) is a common chronic noninfectious disease, which can make the body experience continuous hyperglycemia and long-term metabolic disorder, and lead to the damage, dysfunction, and failure of the whole body’s tissues and organs [1]. According to the International Diabetes Federation, 463 million people worldwide currently have diabetes. Further, this number is expected to increase to 592 million, implying that there will be around a 50% increase in diabetes by 2035 [2]. DM is classified into type 1 diabetes mellitus (T1DM) and type 2 diabetes mellitus (T2DM), in which T2DM accounts for nearly 95% of individuals. As insulin resistance is a major characteristic of T2DM, improving insulin resistance is a primary strategy to improve metabolic control in subjects with type 2 diabetes [3]. Glucose transporter type 4 (GLUT-4) is predominantly expressed in muscle cells and adipocytes [4]. The insulin-stimulated glucose uptake is performed through the solute carrier family 2, facilitated glucose transporter type 4, which is rapidly translocated to the plasma membrane in response to the hormone [5]. Therefore, this protein has a potential role in preventive or therapeutic approaches for diabetes.

**Figure 1:**
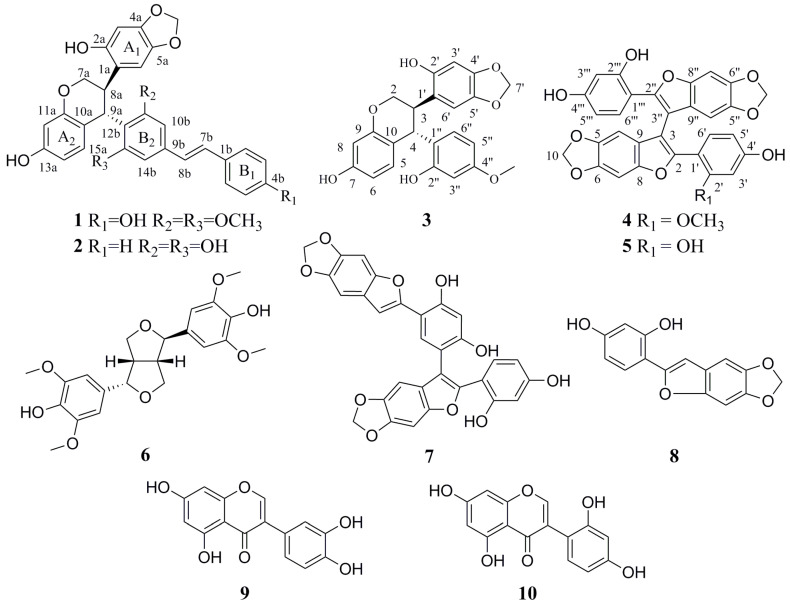
The structures of compounds **1**–**10**.

Natural products (NPs), including herbal formulas and its extracts, have been used to treat human diseases with the unique system of theories and therapies for thousands of years, which have also been increasingly applied to treat T2DM. Much evidence has indicated that herbal medicines and their active ingredients possess anti-diabetics properties with less toxicity and fewer adverse effects [6]. *Sophora davidii* (Franch.) Skeels (Fabaceae family) is a deciduous shrub or dunga-runga growing in valley scrub, hill slopes, and sandy places in valleys below 3400 m. It is mainly distributed in Gansu, Guangxi, Guizhou, Hebei, Henan, Hubei, Hunan, Jiangsu, Shaanxi, Sichuan, Xizang, Yunnan, and Zhejiang provinces of China [7]. The roots of *S. davidii* have been traditionally used to clear heat, sooth a sore throat, cool the blood and reduce swelling, as well as treat hematochezia, cough and dysentery, etc. [8]. In the previous paper, we reported that the flavonoid-rich extract of *S. davidii* showed a good effect in promoting GLUT-4 translocation and improving glucose uptake in L6 cells [9]. Further, we isolated and determined five new compounds, davidones A-E, and one new isoflavonoid, cyclolicoisoflavones A_3_, along with seven known compounds, leachianone A, brosimacutin C, crotalarin, gerontoisoflavone A, griffonianone H, acacetin, and pterostilbene from the roots of *S. davidii* with some GLUT-4 translocation activities [10]. As a continuation of our search for new bioactive natural chemical substances from *S. davidii*, we further performed purification of an EtOAc fraction of the traditional herb that led to two new stilbene oligomers, Davidiol E–F (**1**–**2**), one new 4-aryl-substituted isoflavan Davidinin A (**3**), and one new 2-arylbenzofuran dimer, Shandougenine C (**4**), together with six known compounds (Figure 1). In this paper, we described the isolation and structural elucidation of the four new compounds as well as the GLUT-4 translocation activities of compounds **1**–**10**.

## 2. Results and Discussion

**Figure 2:**
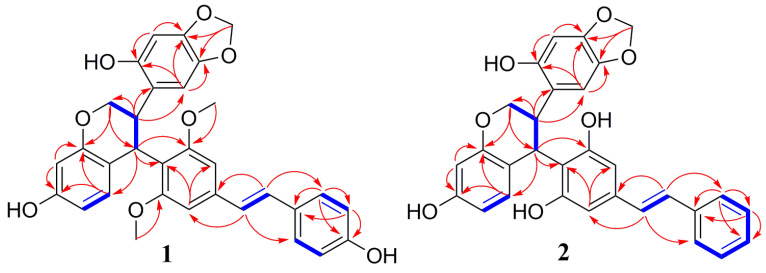
Key ^1^H–^1^H COSY (**─**) and HMBC (→) correlations of **1** and **2**.

**Figure 3:**
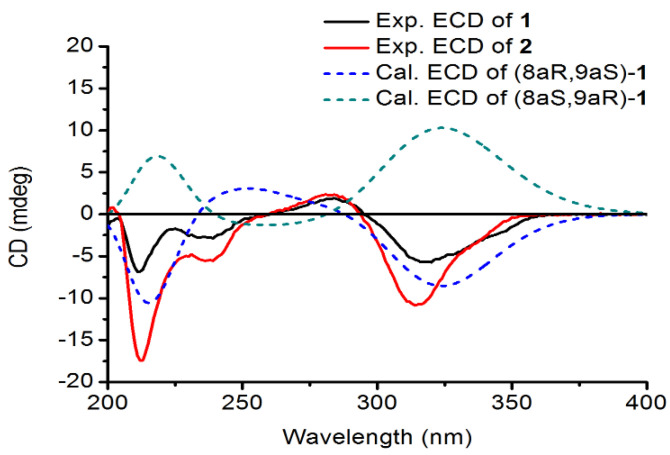
Experimental and calculated electronic circular dichroism (ECD) spectra of compounds **1** and **2**.

The IR spectrum of showed Davidiol E (**1**) the presence of hydroxyl (3327 cm^−1^) and aromatic (1647 cm^−1^ and 1450 cm^−1^) structures. The UV spectrum showed *λ*_max_ (MeOH) (log *ε*) at 230 (3.40) and 310 (3.84) nm [11]. The ^1^H NMR spectrum (Table 1) showed the presence of two *para*-coupled aromatic proton moieties on ring A_1_ at *δ*_H_ 6.76 (1H, s, H-6a), *δ*_H_ 6.30 (1H, s, H-3a), and an ABX spin system at δ_H_ 6.28 (1H, d, *J* = 8.3 Hz, H-15a), *δ*_H_ 6.17 (1H, d, *J* = 1.7 Hz, H-12a), and *δ*_H_ 6.11 (1H, dd, *J* = 8.3, 1.7 Hz, H-14a) for ring A_2_, and two groups of *meta*-coupled aromatic protons belonging to rings B_2_ at *δ*_H_ 6.79 (1H, br s, H-10b), *δ*_H_ 6.70 (1H, br s, H-14b), and 4-hydroxyphenyl group (ring B_1_) at *δ*_H_ 7.38 (2H, d, *J* = 8.4 Hz, H-2b/6b), *δ*_H_ 6.75 (2H, d, *J* = 8.4 Hz, H-3b/5b). The ^1^H NMR spectrum also displayed the presence of a *trans*-1,2-disubstituted vinyl group at *δ*_H_ 7.12 (1H, d, *J* = 16.3 Hz, H-7b), *δ*_H_ 6.91 (1H, d, *J* = 16.3 Hz, H-8b), and a methylenedioxy moiety with two non-equivalent protons at *δ*_H_ 5.82 (1H, s, -OCH_2_O-) and *δ*_H_ 5.78 (1H, s, -OCH_2_O-). The HMBC correlations of this -OCH_2_O- group (*δ*_H_ 5.82, *δ*_H_ 5.78) with C-4a (*δ*_C_ 145.4) and C-5a (*δ*_C_ 139.6) indicated that the oxygen atoms were linked to carbons C-4a and C-5a in the tetrasubstituted aromatic ring A_1_. The ^13^C NMR spectrum of **1** revealed the presence of two methoxy groups at *δ*_C_ 56.2 and 55.8, four aliphatic carbons at *δ*_C_ 100.3, 70.6, 34.7, and 34.4, besides 26 aromatic and olefinic carbons between *δ*_C_ 97.3 and 158.9, and all protonated carbons were assigned from the HMQC spectrum. In the HMBC spectrum (Figure 2), the long-range correlations of H-7b with C-2b, C-6b (*δ*_C_ 127.8), and C-9b (*δ*_C_ 137.3) and H-8b with C-1b (*δ*_C_ 128.2), C-10b (*δ*_C_ 102.3), and C-14b (*δ*_C_ 104.0) indicated that the *trans*-1,2-disubstituted vinyl group was attached to B_1_ and B_2_ rings. The correlations from H-7a to C-9a (*δ*_C_ 34.7) and C-11a (*δ*_C_ 154.5), from H-9a to C-10a (*δ*_C_ 117.6), C-11a (*δ*_C_ 154.5), C-14a (*δ*_C_ 107.7), and C-15a (*δ*_C_ 128.0), and the connections of H-7a/H-8a/H-9a were determined from the H^1^-H^1^ COSY, indicating that the H-7a, H-8a, and H-9a aliphatic protons could be assigned to the protons of the tetrahydrobenzopyran ring. The tetrahydrobenzopyran ring attached to A_1_ ring and B_2_ ring were also confirmed by the correlations between H-7a with C-1a (*δ*_C_ 117.8) and between H-8a with C-1a (*δ*_C_ 117.8), C-2a (*δ*_C_ 150.0), and C-6a (*δ*_C_ 107.3) and between H-9a with C-10b (*δ*_C_ 102.3), C-11b (*δ*_C_ 158.9). The relative configuration of H-8a and H-9a was inferred by the observed large coupling constants (*J* = 11.4 Hz) for them and confirmed by correlations between H-8a with H-10b, and between H-9a with H-3a and H-6a in the ROESY spectrum. On the basis of these observations, H-8a and H-9a were in the opposite orientation [12]. As for the absolute configuration of **1**, electronic circular dichroism (ECD) calculations of the enantiomers 8aR, 9aS-1, and 8aS, 9aR-1 were carried out using B3LYP/6-31G(d) optimized geometries at the B3LYP/6-311+G(d,p) level in MeOH. The experimental and calculated ECD spectra of **1** were in good agreement. The calculated ECD spectrum for **1** showed the positive Cotton effect at 281 nm and the negative Cotton effect around 320 nm in the ECD spectrum (Figure 3). Therefore, Davidiol E was determined to be (8aR,9aS)-8a,9a-*trans*-2a,13a-dihydroxy-4a,5a-methylenedioxy-9a-[(*E*)-6-(3,5-dimethoxyphenyl)-5-(4’-hydroxyphenyl)-ethenyl]-isoflavan.

The ^1^H and ^13^C-NMR spectra data of Davidiol F (**2**) (Table 1) were very similar to those of **1**, except for a monosubstituted phenyl group at *δ*_H_ 7.55 (2H, d, *J* = 7.4 Hz, H-2b/6b), *δ*_H_ 7.34 (2H, t, *J* = 7.8 Hz, H-3b/5b), and *δ*_H_ 7.24 (1H, t, *J* = 7.6 Hz, H-4b) for ring B_2_. Meanwhile, the absence of two methoxy carbons at C-11b-OMe and C-13b-OMe was observed in the ^13^C NMR spectrum. Compound **2** had the same stereochemistry as **1**, which was substantiated by the similar ROESY spectrum and the large coupling constant of H-8a/H-9a (*J* = 11.7 Hz). In a similar manner to **1**, the absolute configuration of **2** was established as 8aR, 9aS by the positive Cotton effect at 284 nm and the negative Cotton effect around 314 nm in the ECD spectrum (Figure 3). Therefore, Davidiol F was determined to be (8a*R*,9a*S*)-8a,9a-*trans*-2a,13a-dihydroxy-4a,5a-methylenedioxy-9a-[(*E*)-6-(3,5-dihydroxyphenyl)-5-phenyl-ethenyl]-isoflavan.

**Figure 4:**
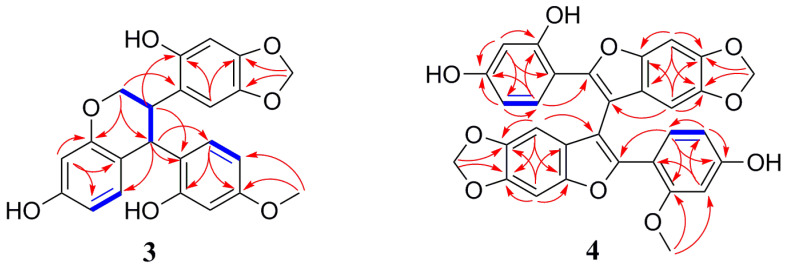
Key ^1^H–^1^H COSY (**─**) and HMBC (→) correlations of **3** and **4**.

**Figure 5:**
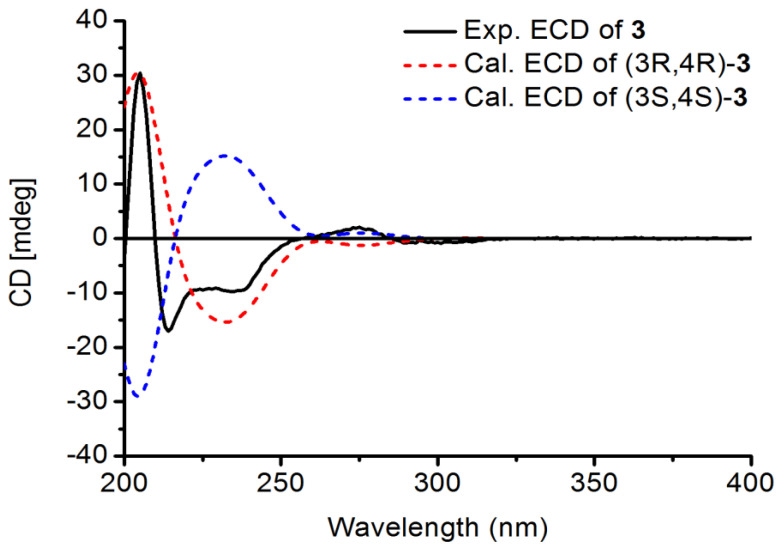
Experimental and calculated ECD spectra of compound **3**.

The IR absorptions of Davidinin A (**3**) suggested the presence of hydroxyl group (3370 cm^−1^), aromatic (1655 and 1452 cm^−1^), and methylenedioxy (-OCH_2_O-) (1115 and 1032 cm^−1^) group. The ^1^H NMR data of **3** (Table 2) established characteristic resonances for two 1,2,4-trisubstituted benzene rings [*δ*_H_ 6.67 (1H, d, *J* = 8.5 Hz, H-6”), *δ*_H_ 6.36 (1H, d, *J* = 2.2 Hz, H-3”), and *δ*_H_ 6.29 (1H, dd, *J* = 8.5, 2.2 Hz, H-5”); and *δ*_H_ 6.54 (1H, d, *J* = 8.0 Hz, H-5), *δ*_H_ 6.26 (1H, br s, H-8), and *δ*_H_ 6.25 (1H, overlapped, H-6)], and a pair of isolated singlet signals at *δ*_H_ 6.72 (1H, s, H-6’) and 6.32 (1H, s, H-3’). A methylenedioxy group signal at *δ*_H_ 5.76, 5.75 (each 1H, br s, H-7”), a methoxyl group at *δ*_H_ 3.70 (3H, s, 4”-OMe), and four aliphatic protons at *δ*_H_ 4.51 (1H, d, *J* = 7.3 Hz, H-4), *δ*_H_ 4.20 (1H, dd, *J* = 10.6 Hz, 3.1 Hz, H-2), *δ*_H_ 4.08 (1H, dd, *J* = 10.6 Hz, 7.1 Hz, H-2), and *δ*_H_ 3.60 (1H, m, H-3) were observed in the ^1^H NMR spectrum. The ^13^C NMR, DEPT (Table 2) and the HSQC spectra showed 23 carbon signals, including eighteen aromatic carbons, a methylenedioxy carbon at *δ*_C_ 101.9 (C-7’), an oxymethylene carbon at *δ*_C_ 69.1 (C-2), a methoxyl carbon at *δ*_C_ 55.5 (4”-OMe), and two methines at *δ*_C_ 39.2 (C-4) and *δ*_C_ 38.9 (C-3). The presence of a benzotetrahydropyran ring was supported by HMBC correlations (Figure 4) from H-2 to C-8 and C-4, and from H-4 to C-5, C-9 and C-10, along with the linkage moiety CH_2_(2)-CH(3)-CH(4) from the ^1^H-^1^H COSY correlations of H-2/H-3/H-4. Moreover, the HMBC correlations from a methylenedioxy proton (*δ*_H_ 5.76, 5.75) with C-4’ (*δ*_C_ 147.6) and C-5’ (*δ*_C_ 142.0) suggested that the methylenedioxy group was located at C-5’, C-6’. The location of ring B and ring C were assigned at C-3 and C-4 by the key HMBC correlations from H-3 to C-1’ and C-2’, and from H-4 to C-1’, C-2’, and C-6’. Regarding the relative configuration of **3**, ROESY cross peaks between H-3/H-6” and H-4/H-6’ suggested that H-3 and H-4 were in the opposite orientation. Finally, the absolute configuration of **3** was determined as 3R, 4R by comparison of theoretical and experimental ECD spectra (Figure 5). Its ECD spectrum displayed a negative Cotton effect at 240 nm and a positive Cotton effect in the range 260–280 nm, which was in agreement with those of Manuifolin Q [13], an analogue whose stereochemistry as (3*R*,4*R*) has been unambiguously elucidated. Thus, Davidinin A was determined to be (3*R*,4*R*)-3,4-*trans*-7,2’-dihydroxy-4’,5’-methylenedioxy-4-(4-methoxy-2-hydroxyphenyl)-isoflavan.

The IR spectrum of shandougenine C (**4**) was similar to that of **3**, also indicating that **4** had (3343 cm^−1^), aromatic (1452 cm^−1^), and methylenedioxy (1032 cm^−1^) groups. The ^1^H NMR spectrum (Table 2) exhibited two sets of ABX system signals at *δ*_H_ 7.36 (1H, d, *J* = 8.4 Hz, H-6’), *δ*_H_ 6.31 (1H, d, *J* = 2.2 Hz, H-3’), and *δ*_H_ 6.42 (1H, dd, *J* = 8.4, 2.2 Hz, H-5’); and at *δ*_H_ 7.17 (1H, d, *J* = 8.3 Hz, H-6‴), *δ*_H_ 6.26 (1H, d, *J* = 2.4 Hz, H-3‴), and *δ*_H_ 6.24 (1H, dd, *J* = 8.3, 2.4 Hz, H-5‴); four singlet signals [*δ*_H_ 7.01 (1H, s, H-7), *δ*_H_ 6.99 (1H, s, H-7”), *δ*_H_ 6.38 (1H, s, H-4”), and *δ*_H_ 6.72 (1H, s, H-4)], two methylenedioxy [*δ*_H_ 5.88, 5.87 (each 2H, overlapped, H-10, H-10”)], and a methoxyl group [*δ*_H_ 3.33 (3H, s, 2’-OMe)] were shown in the ^1^H NMR spectrum. Furthermore, two sets of similar carbon chemical shift values were found in the ^13^C NMR spectrum, which suggested that **4** might be a dimer. These spectroscopic characteristics of compound **4** were similar to the known compound shandougenine B [14], a 2-arylbenzofuran dimer previously isolated from the roots of *Sophora tonkinensis*. The difference was that 2’-OH was substituted by a methoxy group in compound **4**. The correlation of H_3_-2’-OMe to C-2’ (*δ*_C_ 159.4) and C-3’ (*δ*_C_ 100.2) suggested that 2’-OMe was linked to C-2’ according to the HMBC spectrum (Figure 4). Thus, shandougenine C was determined to be 3,3”-bis[2-(2-methoxy-4-hydroxyphenyl)-2-(2,4-dihydroxyphenyl)-5,6-methylenedioxybenzofuran].

The known compounds Shandougenine A (**5**) [14], (+)-Lirioresinol-A (**6**) [15], Shandougenine B (**7**) [14], 2-(2’,4’-dihydroxyphenyl)-5,6-methylenedioxybenzofuran (**8**) [16], isoluteolin (**9**) [17], and 2’,4’,5,7-tetrahydroxyisoflavone (**10**) [18] were identified by comparison of their spectroscopic data with those in the literature.

**Figure 6:**
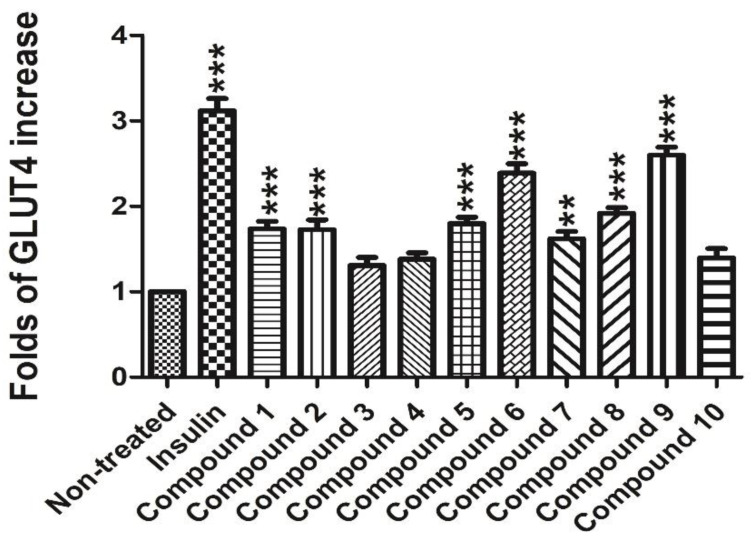
GLUT-4 translocation activities of compounds **1–10** (** *p* < 0.01, compared with non-treated groups; *** *p* < 0.001, compared with non-treated group).

**Figure 7:**
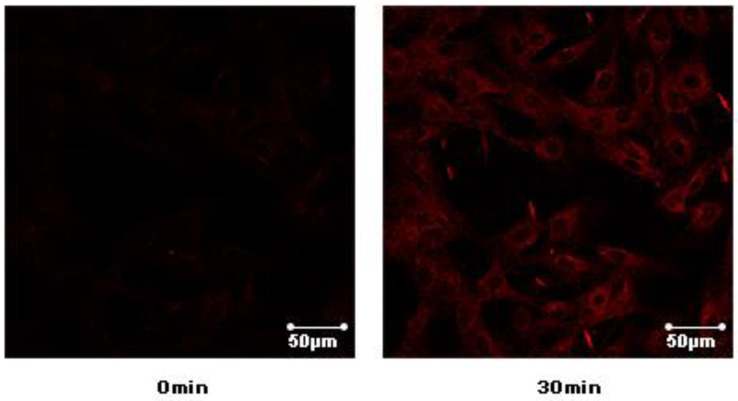
Effects of compound **9** on stimulating GLUT-4 translocation in L6 cells.

In order to test the potential GLUT-4 translocation activity of compounds **1**–**10**, a L6 cell line which stably expressed Myc-GLUT4-mOrange was used to evaluate the effects. Insulin (100 nM) was used as the positive control. The compounds Davidiol E-F (**1–2**), Davidinin A (**3**), Shandougenine C (**4**), Shandougenine A (**5**), Shandougenine B (**7**), 2-(2’,4’-dihydroxyphenyl)-5,6-methylenedioxybenzofuran (**8**) and 2’,4’,5,7-tetrahydroxyisoflavone (**10**) exerted weak activity, increasing GLUT-4 translocation by 0.41–0.92 folds, respectively. (+)-Lirioresinol-A (**6**) possesses a moderate effect on promoting GLUT-4 translocation, which increased GLUT-4 translocation to 2.39 folds. Isoluteolin (**9**) was the most active compound, exhibiting good GLUT-4 translocation activity with 1.60-fold enhancement (Figure 6). Compared with **10**, they all exhibited a set of ABX system signals on B ring; the only difference was the hydroxyl group connected to 3’ and 4’ of B ring in compound **9**, which may enhance the activities of GLUT-4 translocation. The laser-scanning confocal microscope LSM 700 (Carl Zeiss, Jena, Germany) was used to detect the fluorescence to indirectly reflect the content of GLUT4 on the plasma membrane in L6 cells (Supplementary Materials S46). The greater intensity of the fluorescence reflects the greater content of GLUT4 on the plasma membrane. Confocal images in L6 cells incubated in the absence (basal) or presence of compound **9** for 30 min (L6 cells were infected with Myc-GLUT4-mOrange in order to detect externalized GLUT-4 by confocal microscopy) (Figure 7).

## 3. Materials and Methods

### 3.1. General Information

UV and IR spectra were determined on a Shimadzu UV-250 spectrometer (Shimadzu (China) Co., Ltd., Shanghai, China) and a Shimadzu FTIR-8400S spectrometer (Shimadzu (China) Co., Ltd., Shanghai, China), respectively. Optical rotations were measured using an Autopol IV-T automatic polarimeter (Rudolph Research Analytical, Hackettstown, NJ, USA). Circular dichroism (CD) spectra were recorded on a JASCO J-720 W spectrophotometer (JASCO China (Shanghai) Co., Ltd., Shanghai, China). The HRESIMS data were recorded on a UHPLC System and the Q Exactive HF Mass Spectrometer (Thermo Fisher Scientific, Waltham, MA, USA). A Thermo 70105-159070 Betasil C18 column (5 μm, 10 mm × 150 mm, Thermo Fisher Scientific, Waltham, MA, USA) was used for semipreparative HPLC. A Waters 2535 HPLC fitted with a 2998 Photodiode Array Detector and a 2707 Autosampler (Waters, Milford, MA, USA) was used for the semipreparative separations. All the solvents used for chromatography were of HPLC-grade and all the other chemicals were of analytical reagent grade. HPLC-grade acetonitrile was purchased from Merck Chemical Company (Darmstadt, Germany). Silica gel (300–400 mesh) was used for medium-pressure column chromatography and GF254 for TLC (Qingdao HaiYang Chemical Group Co., Qingdao, China). Sephadex LH-20 (Amersham Pharmacia Biotech Co., Piscataway, NJ, USA) was also used for column chromatography.

### 3.2. Materials

The roots of *S. davidii* (Franch.) Skeels (age 12–15 years) were collected from Xiuwen county, Guizhou province, China (at altitudes of 1200 to 1300 m), in June 2014. The roots were dried at room temperature, macerated into a fine powder, and stored at room temperature. The identification was done by Professor Dingrong Wan of School of Pharmaceutical Sciences, South-Central University for Nationalities (SCUN), Wuhan, China. A voucher specimen (SC0801) is deposited in School of Pharmaceutical Sciences, SCUN, Wuhan, China.

### 3.3. Extraction and Isolation

Air-dried roots of *Sophora davidii* (18 kg) were triturated and then extracted with 80% EtOH (4 × 20 L, 3 days each) at room temperature. The EtOH extract (850 g) was suspended in H_2_O (2.0 L) and then partitioned successively with petroleum ether (PE) (4 × 10 L), ethyl acetate (EtOAc) (4 × 10 L), and n-butyl alcohol (*n*-BuOH) (4 ×10 L) to give a PE extract (90 g), EtOAc extract (215 g), and *n*-BuOH extract (110 g), respectively. The EtOAc extraction (200 g) was separated into sixteen fractions (F1–F16) by silica-gel column chromatography (300–400 mesh) eluting with a gradient solvent system of CH_2_Cl_2_/MeOH (200:1, 100:1, 80:1, 60:1, 40:1, 20:1, 10:1, 5:1, 3:1, 0:1, *v*/*v*). The fraction 5 (2.60 g) was subjected to a Sephadex LH-20 (eluted with MeOH) to afford six subfractions (F5-1 to F5-6). F5-2 was applied to semi-preparative HPLC (CNCH_3_/H_2_O, 15:85–25:75, 20 min) at a rate of 4 mL/min, an injection volume of 200 μL, and UV at 254 nm with column temperature at 30 °C to obtain compound **6** (t_R_ = 16.25 min; 56.3 mg). The subfraction F5-6 was further purified by semi-preparative HPLC (CNCH_3_/H_2_O, 25:75–55:45, 20 min) at a rate of 4 mL/min, an injection volume of 100 μL, and UV at 254 nm with column temperature at 30 °C to give **8** (t_R_ = 15.10 min; 5.3 mg). Fraction 8 (5.63 g) was chromatographed on a silica gel column (100–200 mesh) eluting with a gradient of CH_2_Cl_2_/MeOH (100:1–1:1, *v*/*v*) to afford five subfractions (F8-1 to F8-5). The subfraction F8-2 was separated by Sephadex LH-20 (eluted with 90% MeOH) and semi-preparative HPLC (CNCH_3_/H_2_O, 40:60–54:46, 20 min) at a rate of 4 mL/min, an injection volume of 100 μL, and UV at 254 nm with column temperature at 30 °C to give **3** (t_R_ = 13.35 min; 2.7 mg). Fraction 9 (2.63 g) was subjected to Sephadex LH-20 with eluted with 90% MeOH to give six subfractions (F9-1 to F9-6). Subfraction F9-6 was purified using semi-preparative HPLC (CNCH_3_/H_2_O, 37:63–80:20, 20 min) at a rate of 4 mL/min, an injection volume of 150 μL, and UV at 254 nm with column temperature at 30 °C to yield **4** (t_R_ = 16.65 min; 8.0 mg). Fraction 11 (2.03 g) was injected into a Sephadex LH-20 and eluted with MeOH and further separated by semi-preparative HPLC (CNCH_3_/H_2_O, 43:57–60:40, 20 min) at a rate of 4 mL/min, an injection volume of 200 μL, and UV at 254 nm with column temperature at 30 °C to give **1** (t_R_ = 11.05 min; 23.5 mg). Fraction 12 (1.62 g) was subjected to Sephadex LH-20 to afford seven subfractions (F12-1 to F12-7). Fraction F12-2 was separated by semi-preparative HPLC (CNCH_3_/H_2_O, 10:90–45:55, 25 min), at a rate of 4 mL/min, an injection volume of 100 μL, and UV at 254 nm with column temperature at 30 °C to afford **10** (t_R_ = 10.86 min; 5.7 mg). Similarly, fraction F12-4 was also separated by semi-preparative HPLC (CNCH_3_/H_2_O, 10:90–50:50, 25 min), at a rate of 4 mL/min, an injection volume of 150 μL, and UV at 254 nm with column temperature at 30 °C to obtain **2** (t_R_ = 21.28 min; 7.3 mg). Fraction 14 (3.60 g) was chromatographed over Sephadex LH-20 eluting with MeOH and afforded six fractions (F14-1 to F14-6). F14-3 was isolated by semi-preparative HPLC (CNCH_3_/H_2_O, 45:55–77:23, 20 min) at a rate of 4 mL/min, an injection volume of 200 μL, and UV at 254 nm with column temperature at 30 °C to give **5** (t_R_ = 17.34 min; 97.8 mg). Fr14-4 was separated by semi-preparative HPLC (CNCH_3_/H_2_O, 30:70–70:30, 20min) at a rate of 4 mL/min, an injection volume of 200 μL, and UV at 254 nm with column temperature at 30 °C to obtain **9** (t_R_ = 11.18 min; 23.6 mg). Fr14-6 was further separated by semi-preparative HPLC (CNCH_3_/H_2_O, 50:50–60:40, 20min) at a rate of 4 mL/min, an injection volume of 150 μL, and UV at 254 nm with column temperature at 30 °C to obtain compound **7** (t_R_ = 14.44 min; 12.5 mg).

#### 3.3.1. Davidiol E (**1**)

${\left[\alpha \right]}_{D}^{20}$ − 294.2 (*c* 0.50, MeOH); UV (MeOH) λ_max_ (log*ε*) = 230 (3.40), 310 (3.84) nm; IR *ν*_max_ = 3327, 2953, 2843, 2115, 1647, 1450, 1111, 1016 cm^−1^; ECD (*c* 0.50, MeOH) λ_max_ (Δ*ε*) = 204 (−0.15), 211 (−2.24), 225 (−0.57), 239 (−0.95), 284 (+0.64), 320 (−1.87) nm; For ^1^H NMR (600 MHz) and ^13^C NMR (150 MHz) data, see Table 1; HRESIMS *m/z* 541.1861 [M + H]^+^ (calcd. for C_32_H_29_O_8_ 541.1857).

#### 3.3.2. Davidiol F (**2**)

${\left[\alpha \right]}_{D}^{20}$ − 263.8 (*c* 0.50, MeOH); UV (MeOH) λ_max_ (log*ε*) = 210 (2.22), 305 (1.71) nm; IR *ν*_max_ = 3414, 2951, 2841, 2129, 1647, 1450, 1112, 1016 cm^−1^; ECD (*c* 0.50, MeOH) λ_max_ (Δ*ε*) = 202 (+0.25), 212 (−5.25), 231 (−1.45), 239 (−1.67), 284 (+0.71), 314 (−3.27) nm; For ^1^H NMR (600 MHz) and ^13^C NMR (150 MHz) data, see Table 1; HRESIMS *m/z* 497.1592 [M + H]^+^ (calcd. for C_30_H_25_O_7_ 497.1595).

#### 3.3.3. Davidinin A (**3**)

${\left[\alpha \right]}_{D}^{20}$ − 79.8 (*c* 0.50, MeOH); UV (MeOH) λ_max_ (log*ε*) = 210 (2.18), 285 (0.60) nm; IR *ν*_max_ = 3370, 2947, 2833, 1655, 1452, 1115, 1032 cm^−1^; ECD (*c* 0.50, MeOH) λ_max_ (Δ*ε*) = 205 (+7.52), 214 (−4.23), 275 (+0.51), 299 (−0.20) nm; For ^1^H NMR (600 MHz) and ^13^C NMR (150 MHz) data, see Table 2; HRESIMS *m/z* 409.1282 [M + H]^+^ (calcd. for C_23_H_21_O_7_ 409.1282).

#### 3.3.4. Shandougenine C (**4**)

UV (MeOH) λ_max_ (log*ε*) = 210 (1.45), 325 (1.00) nm; IR *ν*_max_ = 3343, 2943, 2832, 1452, 1032 cm^−1^; For ^1^H NMR (600 MHz) and ^13^C NMR (150 MHz) data, see Table 2; HRESIMS *m/z* 575.0949 [M + Na]^+^ (calcd. for C_31_H_20_NaO_10_, 575.0949).

### 3.4. ECD Calculations

The conformational search was performed on Spartan’14 using the MMFF (Merck molecular forcefield) [19]. The conformers with a Boltzmann population of over 1% were chosen for further optimized by the DFT method at the B3LYP/6-31G(d) level in the gas phase. The calculation of ECD was conducted in MeOH using TDDFT at the B3LYP/6-311+G(d,p) level for all conformers of compounds **1** and **3**. All theoretical calculations were performed using the Gaussian 09 program package [20]. The IEF-PCM solvent model for MeOH was used [21]. The ECD data were processed with SpecDis [22] using σ value of 0.3 eV and UV correction of −3 nm.

### 3.5. GLUT-4 Translocation Assay

Construction of myc-GLUT4-mOrange plasmid and cell line were performed as described previously [23]. Myc-GLUT4-mOrange-L6 cells were cultured on glass coverslips for 12 h, and then L6 myoblasts were differentiated to L6 myotubes. Cells were starved in a PSS solution for 2 h. After starvation, mOrange fluorescence was detected by laser-scanning confocal microscopy at an excitation wavelength of 555 nm. Cells were treated with 10 μg/mL tested samples and images were taken every 5 min over a period of 30 min. Zen 2010 Software (Carl Zeiss, Jena, Germany) was used to analyze the fluorescence intensity of mOrange. The detailed method of GLUT-4 fusion with the plasma membrane was described in previous reports [24].

## 4. Conclusions

In this study, two new stilbene oligomers Davidiol E–F (**1**–**2**), one new 4-aryl-substituted isoflavan Davidinin A (**3**), and one new 2-arylbenzofuran dimer, Shandougenine C (**4**), as well as six known compounds (**5**–**10**) were obtained from the roots of *Sophora davidii*. These compounds presented the effects of stimulated GLUT-4 translocation in L6 cells for 1.28–2.60 folds, respectively. Specifically, compound **9** exerted the strongest activity for GLUT-4 translocation with 1.60 fold enhancement and compound **6** showed moderate translocation activity with increasing GLUT-4 translocation by 1.39 fold. Our research suggested that compounds **6** and **9** could offer promising lead structures with GLUT-4 translocation activity, which could be meaningful to the development of pharmaceutical products. Meanwhile, it also provided a clue for potentially active anti-diabetic constituents in the plants of genus *Sophora*.

## Figures and Tables

**Table 1:** ^1^H and^13^ C NMR data of compounds **1** and **2** (δ_H_ in ppm, *J* in Hz).

No.	1		2	
	*δ*c	*δ*_H_ Mult. (*J* in Hz)	*δ*c	*δ*_H_ Mult. (*J* in Hz)
1a	117.8		118.2	
2a	150.0		150.1	
3a	97.3	6.30, s	97.3	6.35, s
4a	145.4		145.4	
5a	139.6		139.5	
6a	107.3	6.76, s	107.4	6.93, s
7a	70.6	4.10, dd (10.3, 3.2)	70.7	4.15, dd (10.2, 3.5)
		3.85, t (10.7)	34.0	3.72, t (10.7)
8a	34.4	4.32, td (11.3, 2.7)	34.2	4.49, td (11.4, 3.5)
9a	34.7	4.89, d (11.6)	117.7	4.82, d (11.7)
10a	117.6		154.7	
11a	154.5		102.2	
12a	102.3	6.17, d (1.7)	155.8	6.15, d (2.4)
13a	155.8		107.7	
14a	107.7	6.11, dd (8.3, 1.7)	128.4	6.13, dd (8.3, 2.4)
15a	128.0	6.28, d (8.3)	137.1	6.38, d (8.3)
1b	128.2		126.4	
2b	127.8	7.38, d (8.4)	128.7	7.55, d (7.4)
3b	115.6	6.75, d (8.4)	128.4	7.34, t (7.8)
4b	157.3		128.7	7.24, t (7.4)
5b	115.6	6.75, d (8.4)	126.4	7.34, t (7.8)
6b	127.8	7.38, d (8.4)	127.2	7.55, d (7.4)
7b	128.4	7.12, d (16.3)	128.9	6.86, d (16.3)
8b	125.4	6.91, d (16.3)	135.7	6.98, d (16.3)
9b	137.3		105.6	
10b	102.3	6.79, s	157.2	6.31, s
11b	158.9		115.4	
12b	117.9		156.9	
13b	158.9		104.2	
14b	104.0	6.70, s	100.3	6.50, s
-OCH_2_O-	100.3	5.82, s	118.2	5.82, d (0.7)
		5.78, s		5.80, d (0.7)
2a-OH		9.06, s		9.11, s
13a-OH		9.05, s		9.01
4b-OH		9.59, s		
11b-OH				8.95, s
13b-OH				9.52, s
11b-OMe	56.2	3.80, s		
13b-OMe	55.8	3.49, s		

^1^H NMR and ^13^C NMR were measured at 600 MHz and 150 MHz in DMSO-*d*_6_.

**Table 2:** ^1^H and^13^ C NMR data of compounds **3** and **4** (*δ*_H_ in ppm, *J* in Hz).

No.	3		4	
	*δ*c	*δ*_H_ Mult. (*J* in Hz)	*δ*c	*δ*_H_ Mult. (*J* in Hz)
2	69.1	4.20, dd, (10.6, 3.1)	151.8	
		4.08, dd, (10.6, 7.1)		
3	38.9	3.60, m	111.2	
4	39.2	4.51, d, (7.3)	99.5	6.72, s
5	132.1	6.54, d, (8.0)	145.5	
6	109.4	6.25, overlapped	147.2	
7	157.7		94.0	7.01, s
8	103.4	6.26, br s	150.9	
9	156.8		123.5	
10	117.7		102.5	5.88, overlapped
1’	120.4		113.4	
2’	150.8		159.4	
3’	98.3	6.32, s	100.2	6.31, d, (2.2)
4’	147.6		161.0	
5’	142.0		108.7	6.42, dd, (8.4, 2.2)
6’	108.6	6.72, s	132.0	7.36, d, (8.4)
7’	101.9	5.76, br s		
		5.75, br s		
1”	124.8			
2”	157.0		151.3	
3”	102.1	6.36, d, (2.2)	110.4	
4”	160.5		99.8	6.38, s
5”	105.9	6.29, dd, (8.5, 2.2)	145.5	
6”		6.67, d, (8.5)	147.2	
7”			93.9	6.99, s
8”			151.0	
9”			123.5	
10”			102.4	5.87, overlapped
1‴			111.3	
2‴			157.3	
3‴			103.8	6.26, d, (2.4)
4‴			160.6	
5‴			108.4	6.24, dd, (8.3, 2.4)
6‴			132.2	7.17, d, (8.3)
2’-OMe			55.3	3.33, s
4”-OMe	55.5	3.70, s		

^1^H NMR and ^13^C NMR were measured at 600 MHz and 150 MHz in MeOH-*d*_4_.

## Data Availability

The data presented in this study are available in Supplementary Material.

## Supplementary Materials

Supplementary Materials are available online. S1: ECD calculation details; S2–S12: UV, IR, HRESIMS, 1D and 2D NMR spectra and ECD spectrum of compound **1**; S13–S23: UV, IR, HRESIMS, 1D and 2D NMR spectra and ECD spectrum of compound **2**; S24–S34: UV, IR, HRESIMS, 1D and 2D NMR spectra and ECD spectrum of compound **3**; S35–S44: UV, IR, HRESIMS, 1D and 2D NMR spectra of compound **4**; S45: The HPLC chromatograms of compounds **1**–**10**; S46: Screening methodology validation.

## Sample Availability

Sample of the compound is available from the authors.
