# Supplementary material for: Chemical Constituents from Roots of Sophora davidii (Franch.) Skeels and Their Glucose Transporter 4 Translocation Activities
Source: Molecules. 2021 Feb 1;26(3):756. doi: 10.3390/molecules26030756 (PMC7867181; doi:10.3390/molecules26030756)
Supplement: Supplementary file 1 [file molecules-26-00756-s001.pdf]

Chemical Constituents from Roots of *Sophora davidii* (Franch.) Skeels and Their Glucose Transporter 4 Translocation Activities

Kangdi Li [1,†], Yuanren Ma [1,†], Tongxi Zhou [1], Xinzhou Yang [1,*] and Ho-Young Choi [2,*]

1 School of Pharmaceutical Sciences, South-Central University for Nationalities, Wuhan 430074, China; kdli@whu.edu.cn (K.L.); 13007136998@163.com (Y.M.); tc13627123095@163.com (T.Z.)

2 College of Korean Medicine, Kyung Hee University, Seoul 02447, Korea

* Correspondence: xzyang@mail.scuec.edu.cn (X.Y.); hychoi@khu.ac.kr (H.-Y.C.); Tel.: +86-27-6784-1196 (X.Y.); +82-2-961-9372 (H.-Y.C.)

† These authors contributed equally to this work.

Supporting Information Contents:

S1. ECD calculation details

S2. UV spectrum of Davidiol E (1)

S3. IR spectrum of Davidiol E (1)

S4. HRESIMS of Davidiol E (1)

S5. ^{1}H NMR spectrum (600 MHz, DMSO-d_6) of Davidiol E (1)

S6. ^{13}C NMR spectrum (150 MHz, DMSO-d_6) of Davidiol E (1)

S7. DEPT 135° spectrum (150 MHz, DMSO-d_6) of Davidiol E (1)

S8. COSY spectrum of Davidiol E (1)

S9. HSQC spectrum of Davidiol E (1)

S10. HMBC spectrum of Davidiol E (1)

S11. ROESY spectrum of Davidiol E (1)

S12. ECD spectrum of Davidiol E (1)

S13. UV spectrum of Davidiol F (2)

S14. IR spectrum of Davidiol F (2)

S15. HRESIMS of Davidiol F (2)

S16. ^{1}H NMR spectrum (600 MHz, DMSO-d_6) of Davidiol F (2)

S17. ^{13}C NMR spectrum (150 MHz, DMSO-d_6) of Davidiol F (2)

S18. DEPT 135° spectrum (150 MHz, DMSO-d_6) of Davidiol F (2)

S19. COSY spectrum of Davidiol F (2)

S20. HSQC spectrum of Davidiol F (2)

S21. HMBC spectrum of Davidiol F (2)

S22. ROESY spectrum of Davidiol F (2)

S23. ECD spectrum of Davidiol F (2)

S24. UV spectrum of Davidinin A (3)

S25. IR spectrum of Davidinin A (3)

S26. HRESIMS of Davidinin A (3)

S27. ^{1}H NMR spectrum (600 MHz, MeOH-d_4) of Davidinin A (3)

S28. ^{13}C NMR spectrum (150 MHz, MeOH-d_4) of Davidinin A (3)

S29. DEPT 135° spectrum (150 MHz, MeOH-d_4) of Davidinin A (3)

S30. COSY spectrum of Davidinin A (3)

S31. HSQC spectrum of Davidinin A (3)

S32. HMBC spectrum of Davidinin A (3)

S33. ROESY spectrum of Davidinin A (3)

S34. ECD spectrum of Davidinin A (3)

S35. UV spectrum of Shandougenine C (4)

S36. IR spectrum of Shandougenine C (4)

S37. HRESIMS of Shandougenine C (4)

S38. ^{1}H NMR spectrum (600 MHz, MeOH-d_4) of Shandougenine C (4)

S39. ^{13}C NMR spectrum (150 MHz, MeOH-d_4) of Shandougenine C (4)

S40. DEPT 135° spectrum (150 MHz, MeOH-d_4) of Shandougenine C (4)

S41. COSY spectrum of Shandougenine C (4)

S42. HSQC spectrum of Shandougenine C (4)

S43. HMBC spectrum of Shandougenine C (4)

S44. ROESY spectrum of Shandougenine C (4)

S45. The HPLC chromatograms of compounds 1-10

S46. Screening methodology validation

S1. ECD calculation details

1. Conformers and Bolzmann distributions of the optimized 8aR, 9aS-1

species	*E'=E+ZPE*	*E*	*H*	*G*	*ΔG*	*ΔE(kcal/mol)*	*p%*
1	-1837.440801	-1837.405775	-1837.404831	-1837.510722	0	0	32.62%
2	-1837.440688	-1837.405706	-1837.404761	-1837.510352	0.00037	0.232178515	22.04%
3	-1837.440453	-1837.405424	-1837.40448	-1837.510314	0.000408	0.256023876	21.17%
4	-1837.440342	-1837.405349	-1837.404405	-1837.510093	0.000629	0.394703475	16.75%
5	-1837.436997	-1837.401506	-1837.400562	-1837.508295	0.002427	1.522965556	2.49%
6	-1837.437004	-1837.401505	-1837.40056	-1837.508006	0.002716	1.704315802	1.83%
7	-1837.436705	-1837.401216	-1837.400272	-1837.507871	0.002851	1.789029584	1.59%
8	-1837.436726	-1837.401207	-1837.400263	-1837.507819	0.002903	1.821660079	1.50%

***E*, *E'*, *H, G*: total energy, total energy with zero point energy (ZPE), enthalpy, and Gibbs free energy**

2. The coordinate for the lowest-energy conformer ECD calculations

8aR, 9aS-1 Conf. 1		Standard Orientation (Ångstroms)		
I	atom	X	Y	Z
1	C	4.307708	3.934187	-0.185681
2	C	3.487249	4.921744	0.354748
3	C	2.265154	4.573209	0.939804
4	C	1.884318	3.235699	0.961832
5	C	2.677374	2.215299	0.420425
6	C	3.906581	2.593876	-0.146231
7	C	2.267026	0.740853	0.459588
8	C	0.759045	0.524869	0.446864
9	C	-0.058874	0.805219	-0.664474
10	C	-1.442465	0.648674	-0.638662
11	C	-2.077538	0.192265	0.527653
12	C	-1.283982	-0.115108	1.642875
13	C	0.103773	0.051242	1.603024
14	O	3.831937	6.247476	0.34366
15	O	4.781333	1.705567	-0.712382
16	C	4.538589	0.317347	-0.470013
17	C	2.813859	-1.531289	-0.638184
18	O	0.908806	-0.213682	2.676621
19	C	-3.528076	0.0117	0.63013
20	C	-4.458733	0.421774	-0.256724
21	C	-5.908129	0.239239	-0.172014
22	C	-6.733607	0.851761	-1.135951
23	C	-8.116578	0.723829	-1.111132

24	C	-8.722007	-0.036304	-0.105586
25	C	-7.925703	-0.662114	0.862551
26	C	-6.543193	-0.525617	0.824874
27	O	-10.084194	-0.136492	-0.119715
28	O	0.608931	1.210822	-1.804584
29	C	-0.134854	1.830001	-2.852493
30	C	0.3249	-0.716421	3.867287
31	C	3.369113	-2.346118	0.377195
32	C	3.181565	-3.708009	0.306038
33	C	2.455574	-4.288587	-0.728992
34	C	1.872376	-3.533278	-1.721879
35	C	2.053652	-2.135799	-1.660102
36	O	3.597581	-4.690644	1.186995
37	C	3.311134	-5.922395	0.523309
38	O	2.396426	-5.651044	-0.545798
39	O	1.463459	-1.428744	-2.673556
40	C	3.055068	-0.033898	-0.63636
41	H	5.266095	4.17511	-0.639416
42	H	1.635982	5.349397	1.362524
43	H	0.930021	2.969732	1.407433
44	H	2.607244	0.328165	1.415144
45	H	-2.02655	0.843707	-1.528321
46	H	-1.767898	-0.472496	2.543988
47	H	4.696021	6.340033	-0.086571
48	H	4.899203	0.063748	0.537165
49	H	5.151865	-0.216574	-1.199775
50	H	-3.853896	-0.494548	1.537623
51	H	-4.129818	0.9666	-1.140874
52	H	-6.273408	1.445983	-1.922091
53	H	-8.741357	1.202188	-1.858676
54	H	-8.39134	-1.261939	1.64293
55	H	-5.950586	-1.032086	1.580823
56	H	-10.368854	-0.688036	0.625628
57	H	0.607775	2.213988	-3.553909
58	H	-0.782021	1.109914	-3.367553
59	H	-0.738006	2.657662	-2.463497
60	H	1.152744	-0.864534	4.563061
61	H	-0.388574	-0.003023	4.300047
62	H	-0.179971	-1.675444	3.694476
63	H	3.932925	-1.912858	1.196755
64	H	1.282673	-3.968426	-2.519888
65	H	4.239037	-6.344626	0.108211

66	H	2.84703	-6.617492	1.228034
67	H	1.324502	-0.501752	-2.403996
68	H	2.783023	0.374433	-1.611521

3. Conformers and Bolzmann distributions of the optimized 8aS, 9aR-1

species	*E'=E+ZPE*	*E*	*H*	*G*	*ΔG*	*ΔE(kcal/mol)*	*p%*
1	-1837.440805	-1837.405751	-1837.404807	-1837.511319	0	0	41.04%
2	-1837.4408	-1837.405775	-1837.40483	-1837.510722	0.000597	0.374623171	21.80%
3	-1837.440438	-1837.405415	-1837.404471	-1837.510522	0.000797	0.500125071	17.64%
4	-1837.440453	-1837.405424	-1837.40448	-1837.510319	0.001	0.6275095	14.22%
5	-1837.436997	-1837.401506	-1837.400562	-1837.50829	0.003029	1.900726275	1.66%
6	-1837.437034	-1837.401509	-1837.400565	-1837.508228	0.003091	1.939631865	1.55%
7	-1837.436707	-1837.401217	-1837.400272	-1837.507889	0.00343	2.152357585	1.08%
8	-1837.436726	-1837.401207	-1837.400263	-1837.507819	0.0035	2.19628325	1.01%

***E*, *E'*, *H, G*: total energy, total energy with zero point energy (ZPE), enthalpy, and Gibbs free energy**

4. The coordinate for the lowest-energy conformer ECD calculations

8aS, 9aR-1 Conf. 1		Standard Orientation (Ångstroms)		
I	atom	X	Y	Z
1	C	-4.688979	-3.624367	-0.121164
2	C	-3.893317	-4.699609	-0.510054
3	C	-2.567502	-4.483822	-0.901081
4	C	-2.060714	-3.188709	-0.882666
5	C	-2.826064	-2.083514	-0.487372
6	C	-4.159484	-2.328765	-0.11755
7	C	-2.271498	-0.656095	-0.47654
8	C	-0.771728	-0.587914	-0.218537
9	C	0.111023	-0.224777	-1.262625
10	C	1.49518	-0.201813	-1.086563
11	C	2.061585	-0.546256	0.151553
12	C	1.201721	-0.899018	1.20262
13	C	-0.180313	-0.912719	1.012795
14	O	-4.361453	-5.986823	-0.532154
15	O	-5.021991	-1.345978	0.288392
16	C	-4.609538	0.00524	0.06717
17	C	-2.759754	1.686325	0.491706
18	O	-1.056703	-1.217185	2.037354
19	C	3.504674	-0.550697	0.404712
20	C	4.487398	-0.270306	-0.476336

21	C	5.929863	-0.273503	-0.230529
22	C	6.805594	0.013844	-1.296633
23	C	8.185294	0.025663	-1.135855
24	C	8.736701	-0.253587	0.118387
25	C	7.890262	-0.540898	1.197394
26	C	6.511817	-0.549121	1.021604
27	O	10.09769	-0.230881	0.232433
28	O	-0.485028	0.083293	-2.454515
29	C	0.325341	0.476415	-3.549812
30	C	-0.55722	-1.89451	3.189097
31	C	-3.061149	2.520779	-0.611148
32	C	-2.75336	3.859897	-0.528527
33	C	-2.148858	4.397937	0.603072
34	C	-1.81067	3.619329	1.687459
35	C	-2.117198	2.244297	1.615742
36	O	-2.923053	4.851764	-1.478101
37	C	-2.622814	6.069239	-0.794374
38	O	-1.925283	5.742316	0.413905
39	O	-1.770616	1.514978	2.722028
40	C	-3.144751	0.219565	0.468303
41	H	-5.72503	-3.761731	0.17914
42	H	-1.958174	-5.327426	-1.207806
43	H	-1.027596	-3.025838	-1.176185
44	H	-2.411155	-0.238601	-1.479153
45	H	2.138682	0.080665	-1.908963
46	H	1.626333	-1.147771	2.168194
47	H	-5.286432	-5.985754	-0.241089
48	H	-5.277232	0.616733	0.678676
49	H	-4.775993	0.260378	-0.989158
50	H	3.776098	-0.818089	1.424864
51	H	4.213313	-0.011334	-1.498263
52	H	6.387552	0.232211	-2.276669
53	H	8.848615	0.248204	-1.965598
54	H	8.314186	-0.756519	2.176891
55	H	5.881401	-0.771877	1.877213
56	H	10.342021	-0.437997	1.147952
57	H	-0.365081	0.679667	-4.370413
58	H	0.89654	1.385661	-3.322761
59	H	1.017006	-0.322333	-3.847962
60	H	-0.001116	-2.79298	2.900144
61	H	0.082273	-1.242099	3.795826
62	H	-1.437179	-2.177612	3.769148

63	H	-3.527534	2.119657	-1.504831
64	H	-1.316185	4.019125	2.564706
65	H	-1.982293	6.692954	-1.423629
66	H	-3.557364	6.593699	-0.54318
67	H	-1.679016	0.571182	2.493313
68	H	-3.0744	-0.185226	1.479359

5. Conformers and Bolzmann distributions of the optimized 3R, 4R-3

species	*E'=E+ZPE*	*E*	*H*	*G*	*ΔG*	*ΔE(kcal/mol)*	*p%*
1	-1414.609168	-1414.584094	-1414.583149	-1414.664672	0	0	18.49%
2	-1414.609175	-1414.584036	-1414.583092	-1414.664645	2.7E-05	0.016942757	17.97%
3	-1414.608961	-1414.583807	-1414.582863	-1414.664469	0.000203	0.127384429	14.91%
4	-1414.608812	-1414.58372	-1414.582776	-1414.664286	0.000386	0.242218667	12.28%
5	-1414.608778	-1414.583635	-1414.582691	-1414.664176	0.000496	0.311244712	10.93%
6	-1414.60823	-1414.5831	-1414.582156	-1414.664089	0.000583	0.365838039	9.97%
7	-1414.608559	-1414.583403	-1414.582459	-1414.66397	0.000702	0.440511669	8.79%
8	-1414.607868	-1414.582721	-1414.581777	-1414.66371	0.000962	0.603664139	6.67%

***E*, *E'*, *H, G*: total energy, total energy with zero point energy (ZPE), enthalpy, and Gibbs free energy**

6. The coordinate for the lowest-energy conformer ECD calculations

3R, 4R-3 Conf. 1		Standard Orientation (Ångstroms)		
I	atom	X	Y	Z
1	C	-0.465558	3.455563	0.57722
2	C	-0.267032	4.13961	-0.619345
3	C	0.209988	3.460965	-1.749322
4	C	0.478978	2.102397	-1.652092
5	C	0.2943	1.380811	-0.463345
6	C	-0.182591	2.085408	0.650683
7	C	0.600305	-0.10179	-0.381554
8	C	-0.238044	-0.746019	0.7831
9	C	-0.050422	0.125433	2.02802
10	O	-0.443662	1.495169	1.856691
11	O	-0.522208	5.477155	-0.749569
12	C	-1.688972	-1.034375	0.414268
13	C	-2.743673	-0.169722	0.795973
14	C	-4.033378	-0.514984	0.46149
15	C	-4.321385	-1.683022	-0.234775
16	C	-3.329921	-2.552216	-0.630947
17	C	-2.001847	-2.210594	-0.301022
18	O	-5.212995	0.14431	0.763837

19	C	-6.21691	-0.537174	0.013161
20	O	-5.680546	-1.798672	-0.409038
21	O	-1.065661	-3.124595	-0.699627
22	C	2.09304	-0.403249	-0.229541
23	C	2.590296	-1.678974	-0.561047
24	C	3.931031	-2.012128	-0.424592
25	C	4.840007	-1.060305	0.058477
26	C	4.38449	0.216928	0.394761
27	C	3.027548	0.520036	0.241402
28	O	1.680784	-2.60064	-1.041357
29	O	6.131911	-1.484182	0.156004
30	C	7.102241	-0.566694	0.639004
31	H	-0.841025	3.958262	1.465122
32	H	0.353505	4.006095	-2.676009
33	H	0.845646	1.571189	-2.5278
34	H	0.268733	-0.563459	-1.318971
35	H	0.233763	-1.703569	1.03973
36	H	-0.653047	-0.239708	2.862972
37	H	1.006702	0.105479	2.32304
38	H	-0.863294	5.814493	0.093273
39	H	-2.543511	0.737075	1.352352
40	H	-3.532748	-3.4666	-1.175621
41	H	-7.091526	-0.710956	0.64583
42	H	-6.485959	0.055846	-0.874382
43	H	-0.162714	-2.759672	-0.685441
44	H	4.29852	-3.001006	-0.687443
45	H	5.061964	0.977581	0.763568
46	H	2.68875	1.521857	0.485568
47	H	2.133182	-3.433507	-1.247022
48	H	8.050477	-1.107043	0.637116
49	H	7.18393	0.312896	-0.012519
50	H	6.87272	-0.239035	1.66123

7. Conformers and Bolzmann distributions of the optimized 3S, 4S-3

species	*E'=E+ZPE*	*E*	*H*	*G*	*ΔG*	*ΔE(kcal/mol)*	*p%*
1	-1414.609168000	-1414.584094000	-1414.583149	-1414.664672	0	0	16.84%
2	-1414.609176000	-1414.584036000	-1414.583092	-1414.664646	2.6E-05	0.016315247	16.38%
3	-1414.608959000	-1414.583805000	-1414.582861	-1414.664469	0.000203	0.127384429	13.58%
4	-1414.608812000	-1414.583720000	-1414.582776	-1414.664286	0.000386	0.242218667	11.19%
5	-1414.608778000	-1414.583635000	-1414.582691	-1414.664176	0.000496	0.311244712	9.96%
6	-1414.608230000	-1414.583100000	-1414.582156	-1414.664089	0.000583	0.365838039	9.08%
7	-1414.608559000	-1414.583403000	-1414.582459	-1414.66397	0.000702	0.440511669	8.00%

8	-1414.607868000	-1414.582722000	-1414.581778	-1414.663712000	0.00096	0.60240912	6.09%
9	-1414.607259000	-1414.581934000	-1414.580989	-1414.663054	0.001618	1.015310371	3.03%
10	-1414.607020000	-1414.581671000	-1414.580727	-1414.662859	0.001813	1.137674724	2.47%
11	-1414.606787000	-1414.581422000	-1414.580478	-1414.662656	0.002016	1.265059152	1.99%
12	-1414.606240000	-1414.581090000	-1414.580146	-1414.661393	0.003279	2.057603651	0.52%
13	-1414.606128000	-1414.580974000	-1414.58003	-1414.661273	0.003399	2.132904791	0.46%
14	-1414.605683000	-1414.580521000	-1414.579576	-1414.660901	0.003771	2.366338325	0.31%
15	-1414.603223000	-1414.577559000	-1414.576615	-1414.659825	0.004847	3.041538547	0.10%

***E*, *E'*, *H, G*: total energy, total energy with zero point energy (ZPE), enthalpy, and Gibbs free energy**

8. The coordinate for the lowest-energy conformer ECD calculations

3S, 4S-3 Conf. 1		Standard Orientation (Ångstroms)		
I	atom	X	Y	Z
1	C	0.465559	3.455563	0.57722
2	C	0.267032	4.13961	-0.619345
3	C	-0.209988	3.460965	-1.749322
4	C	-0.478978	2.102397	-1.652092
5	C	-0.2943	1.380811	-0.463345
6	C	0.182591	2.085408	0.650683
7	C	-0.600305	-0.10179	-0.381554
8	C	0.238044	-0.746019	0.7831
9	C	0.050422	0.125433	2.02802
10	O	0.443662	1.495169	1.856691
11	O	0.522208	5.477155	-0.749569
12	C	1.688972	-1.034375	0.414268
13	C	2.743673	-0.169722	0.795973
14	C	4.033378	-0.514984	0.46149
15	C	4.321385	-1.683022	-0.234775
16	C	3.329921	-2.552216	-0.630947
17	C	2.001847	-2.210594	-0.301022
18	O	5.212995	0.14431	0.763837
19	C	6.21691	-0.537174	0.013161
20	O	5.680546	-1.798672	-0.409038
21	O	1.065661	-3.124595	-0.699627
22	C	-2.09304	-0.403249	-0.229541
23	C	-2.590296	-1.678974	-0.561047
24	C	-3.931031	-2.012128	-0.424592
25	C	-4.840007	-1.060305	0.058477
26	C	-4.38449	0.216928	0.394761
27	C	-3.027548	0.520036	0.241402

28	O	-1.680784	-2.60064	-1.041357
29	O	-6.131911	-1.484182	0.156004
30	C	-7.102241	-0.566694	0.639004
31	H	0.841025	3.958262	1.465122
32	H	-0.353504	4.006095	-2.676009
33	H	-0.845646	1.571189	-2.5278
34	H	-0.268733	-0.563459	-1.318971
35	H	-0.233763	-1.70357	1.03973
36	H	-1.006702	0.105479	2.32304
37	H	0.653047	-0.239708	2.862972
38	H	0.863294	5.814493	0.093273
39	H	2.543511	0.737075	1.352352
40	H	3.532748	-3.4666	-1.175621
41	H	6.485959	0.055846	-0.874381
42	H	7.091526	-0.710956	0.64583
43	H	0.162714	-2.759672	-0.685441
44	H	-4.29852	-3.001006	-0.687443
45	H	-5.061964	0.977581	0.763568
46	H	-2.68875	1.521857	0.485568
47	H	-2.133182	-3.433507	-1.247022
48	H	-8.050477	-1.107043	0.637116
49	H	-6.87272	-0.239035	1.66123
50	H	-7.18393	0.312896	-0.012519

S2. UV spectrum of Davidiol E (1)

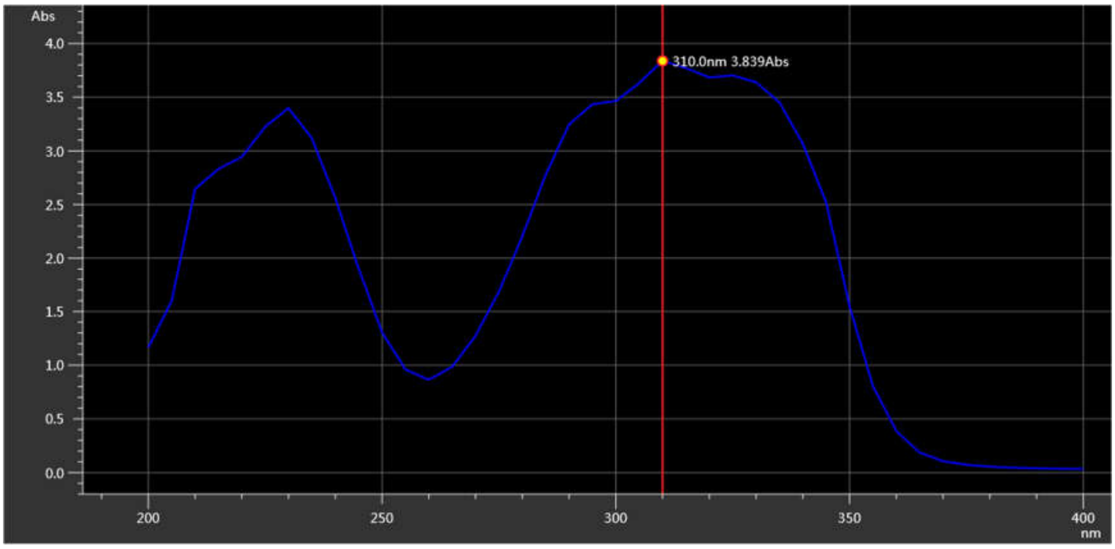

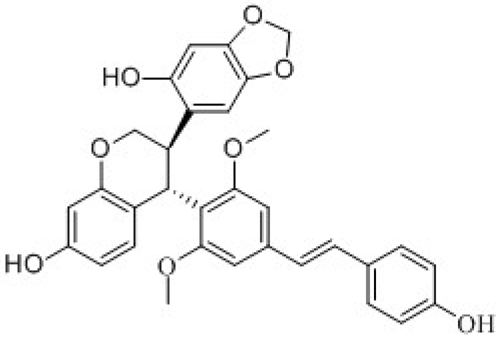

S3. IR spectrum of Davidiol E (1)

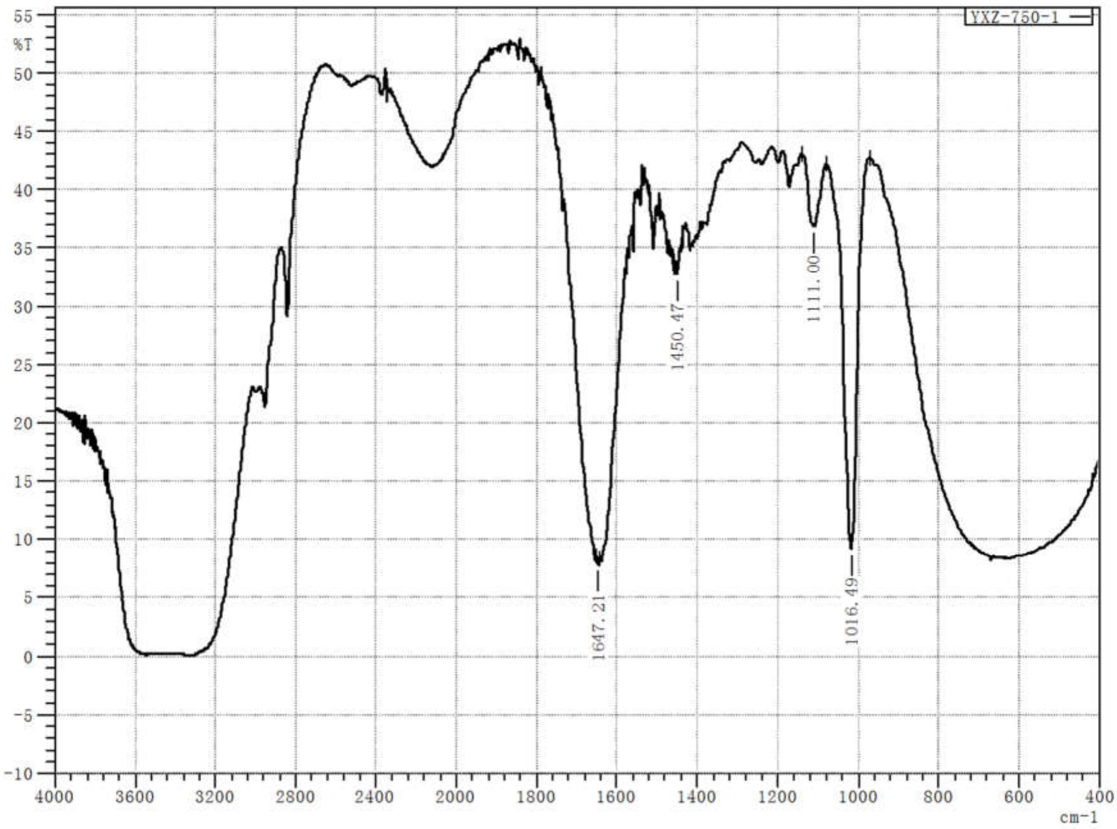

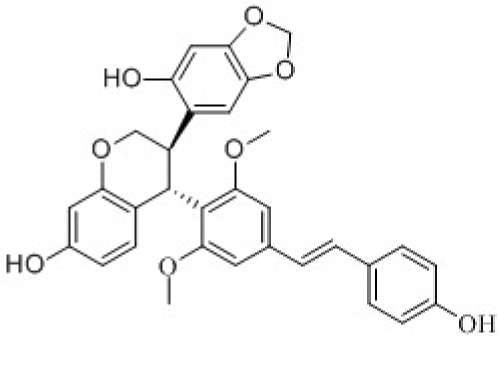

S4. HRESIMS of Davidiol E (1)

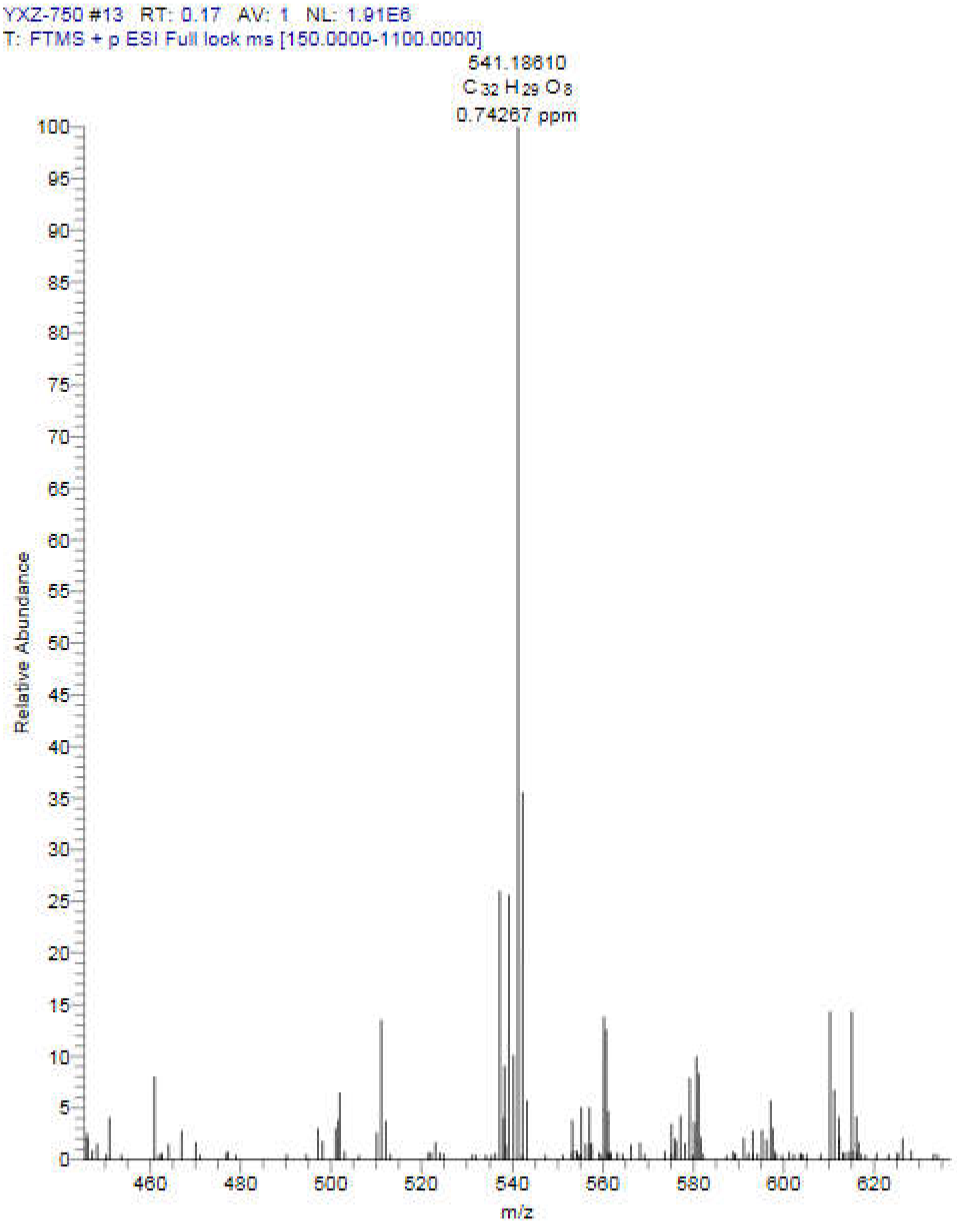

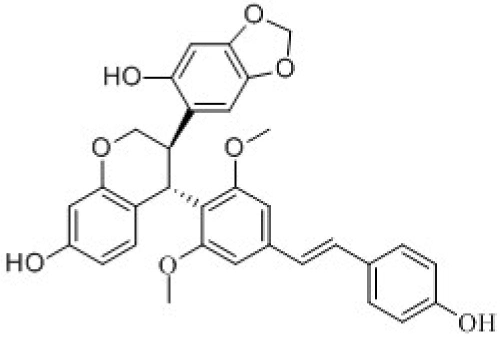

S5. ^{1}H NMR spectrum (600 MHz, DMSO-d_6) of Davidiol E (1)

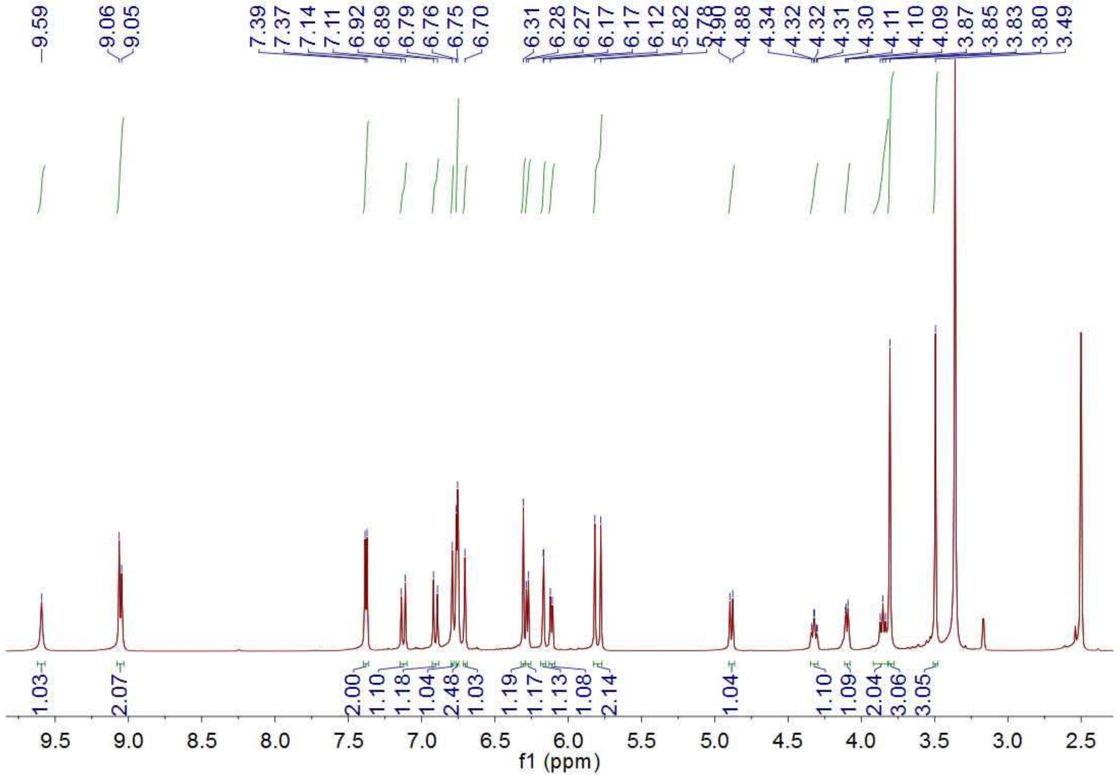

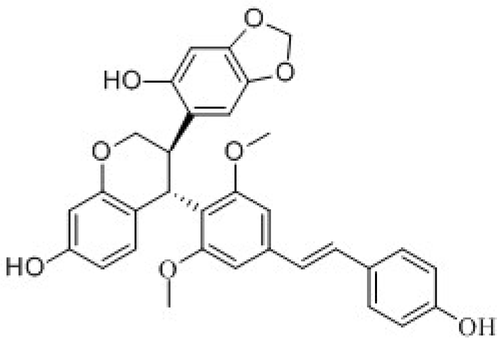

S6. ^{13}C NMR spectrum (150 MHz, DMSO-d_6) of Davidiol E (1)

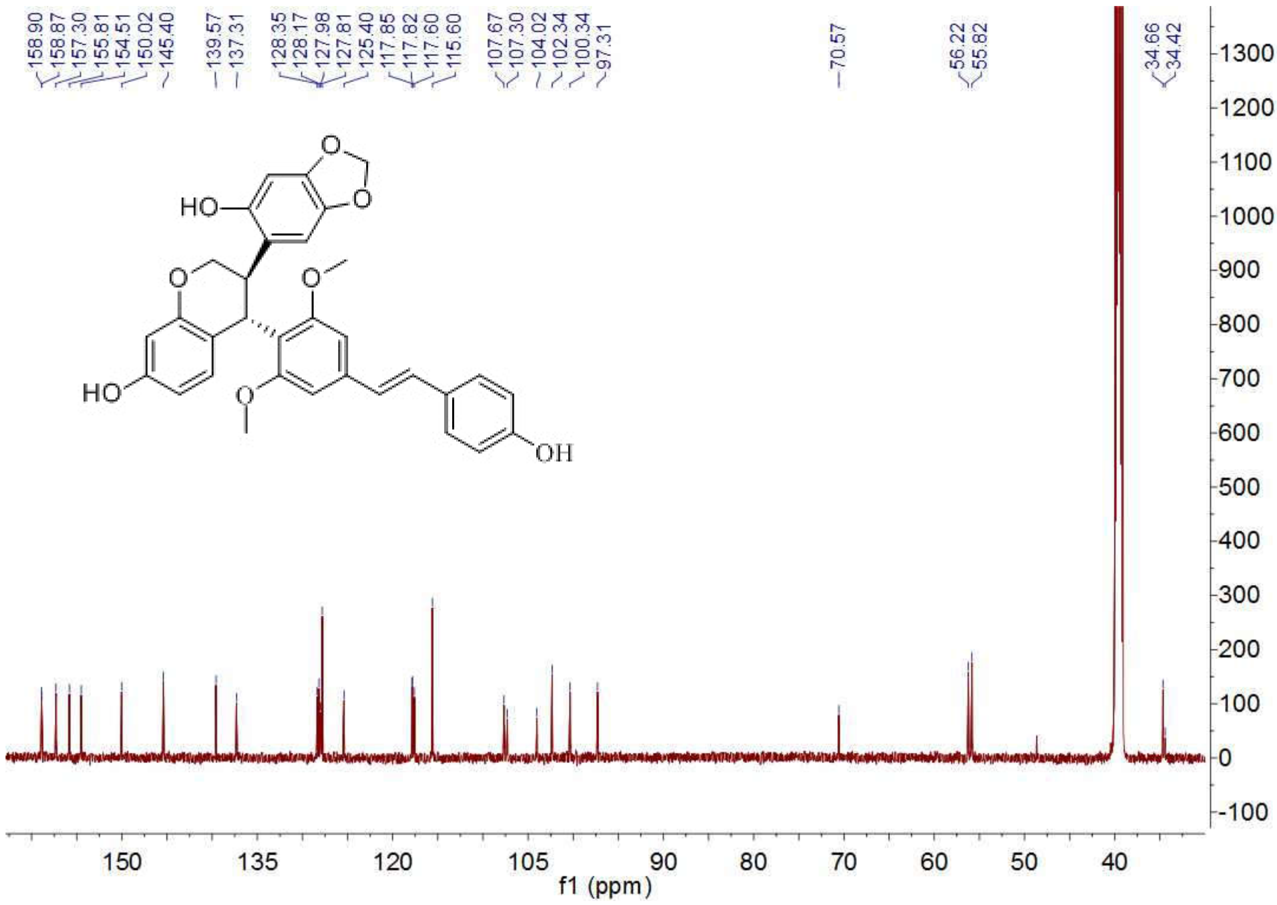

S7. DEPT 135º spectrum (150 MHz, DMSO-d_6) of Davidiol E (1)

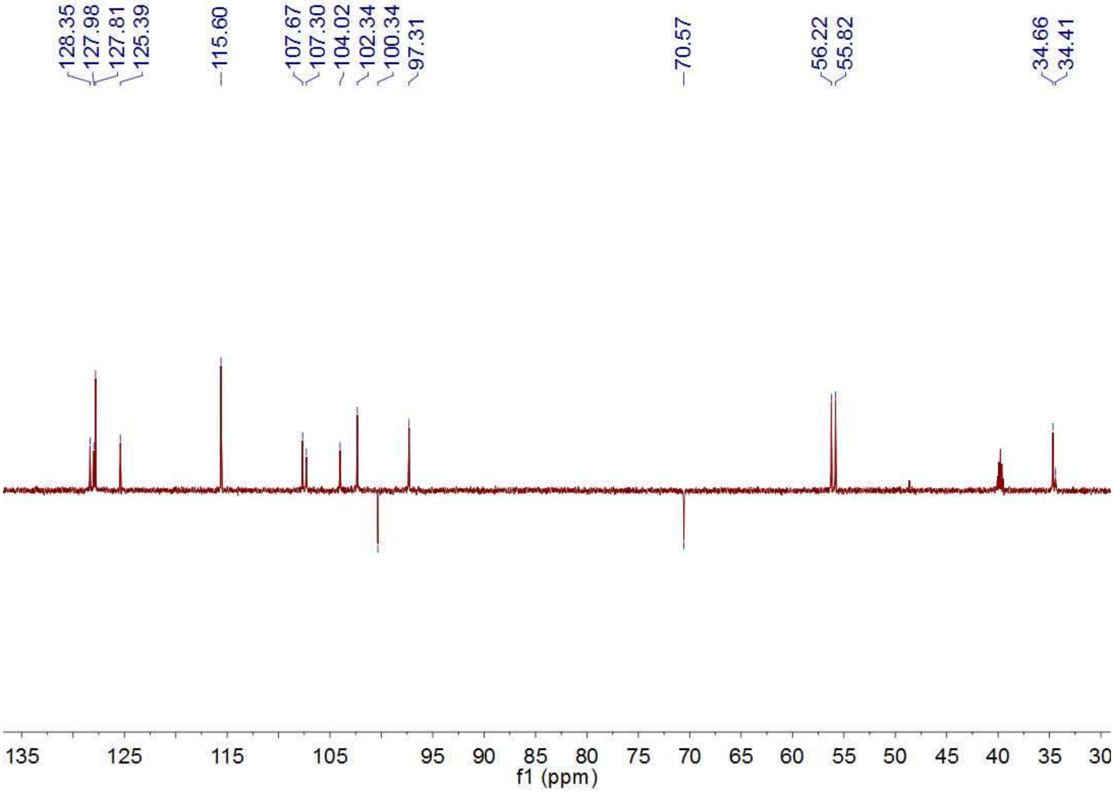

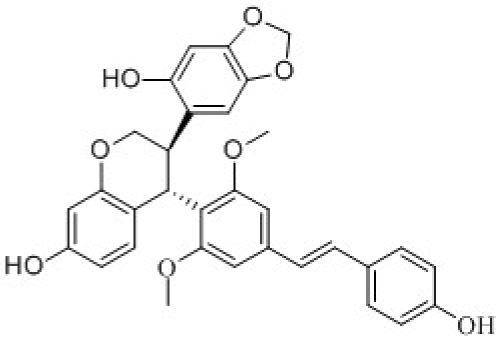

S8. COSY spectrum of Davidiol E (1)

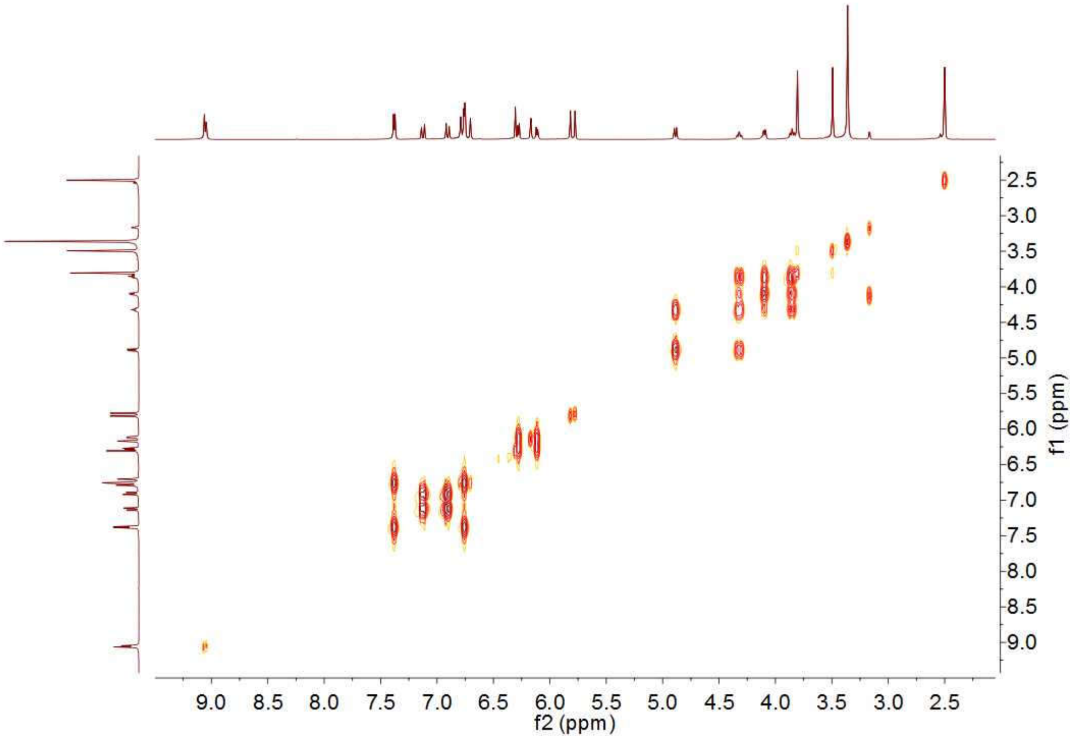

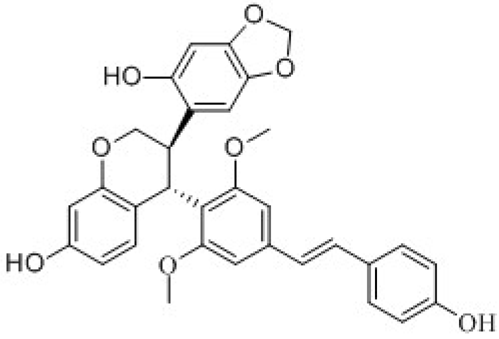

S9. HSQC spectrum of Davidiol E (1)

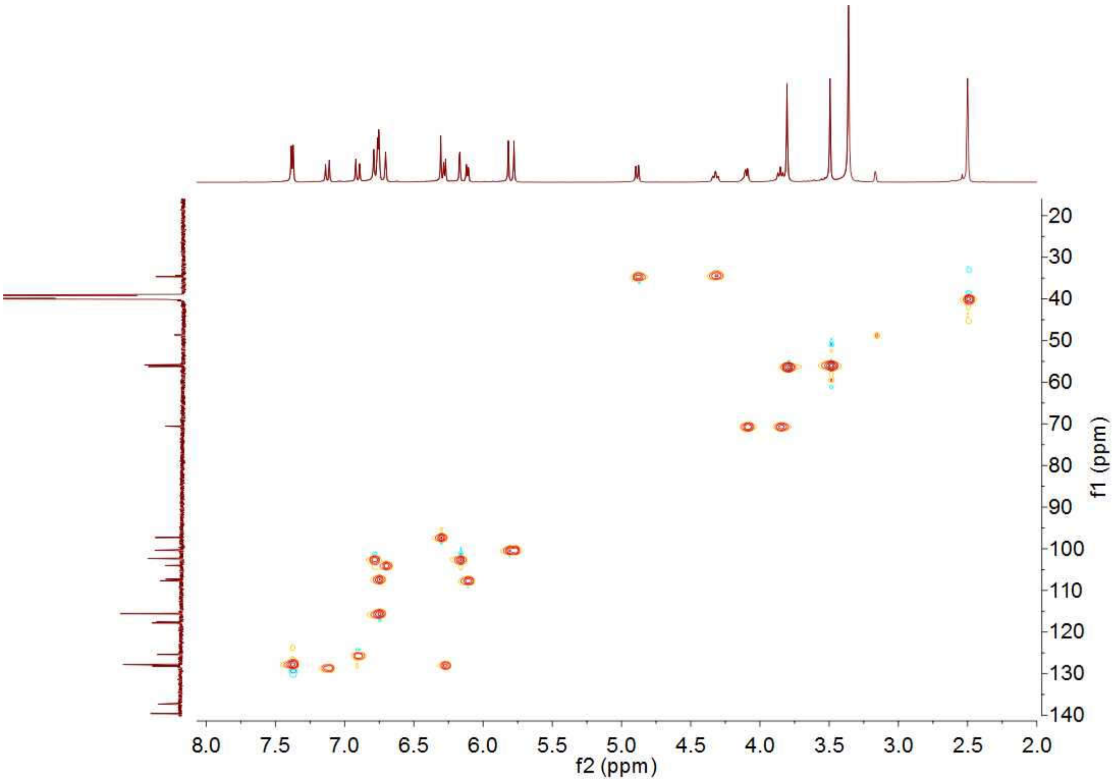

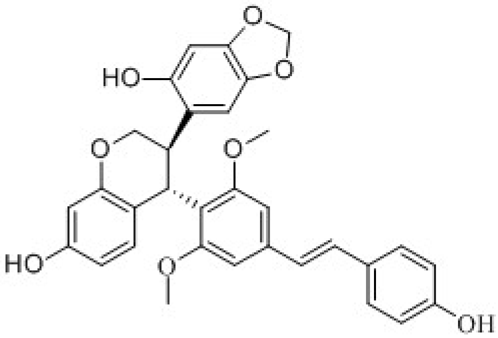

S10. HMBC spectrum of Davidiol E (1)

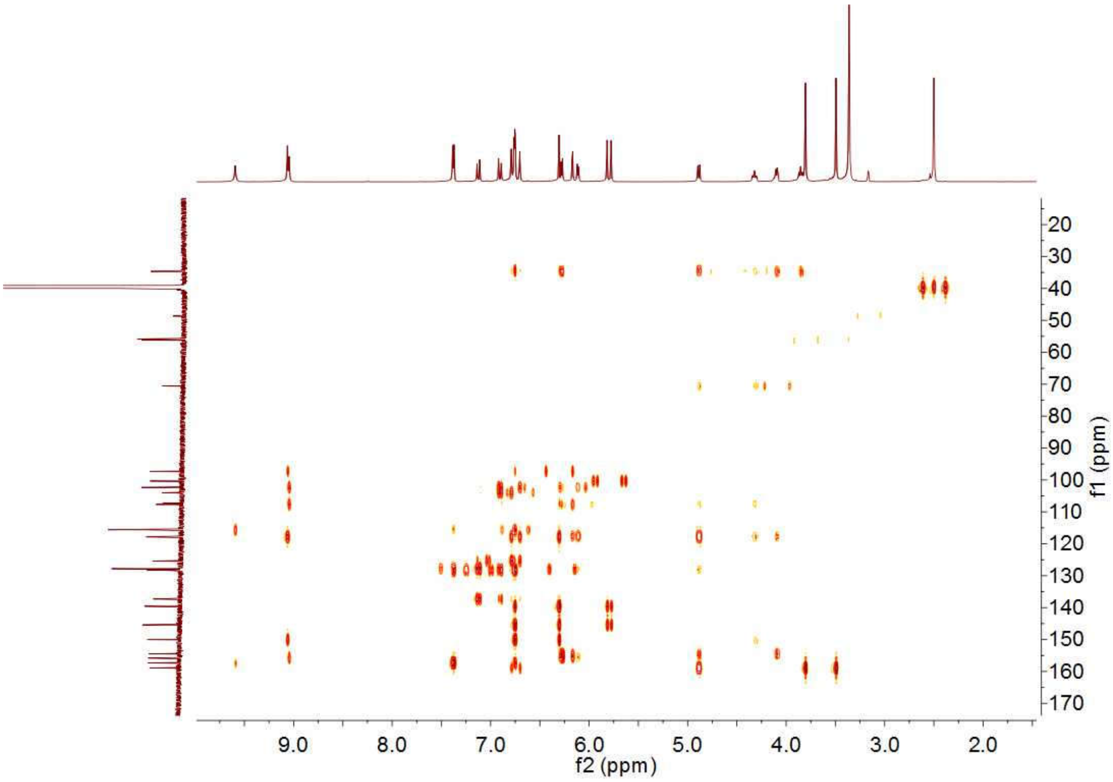

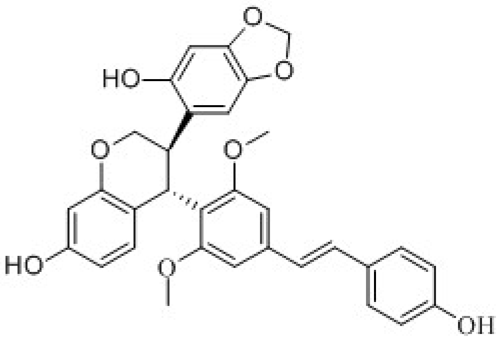

S11. ROESY spectrum of Davidiol E (1)

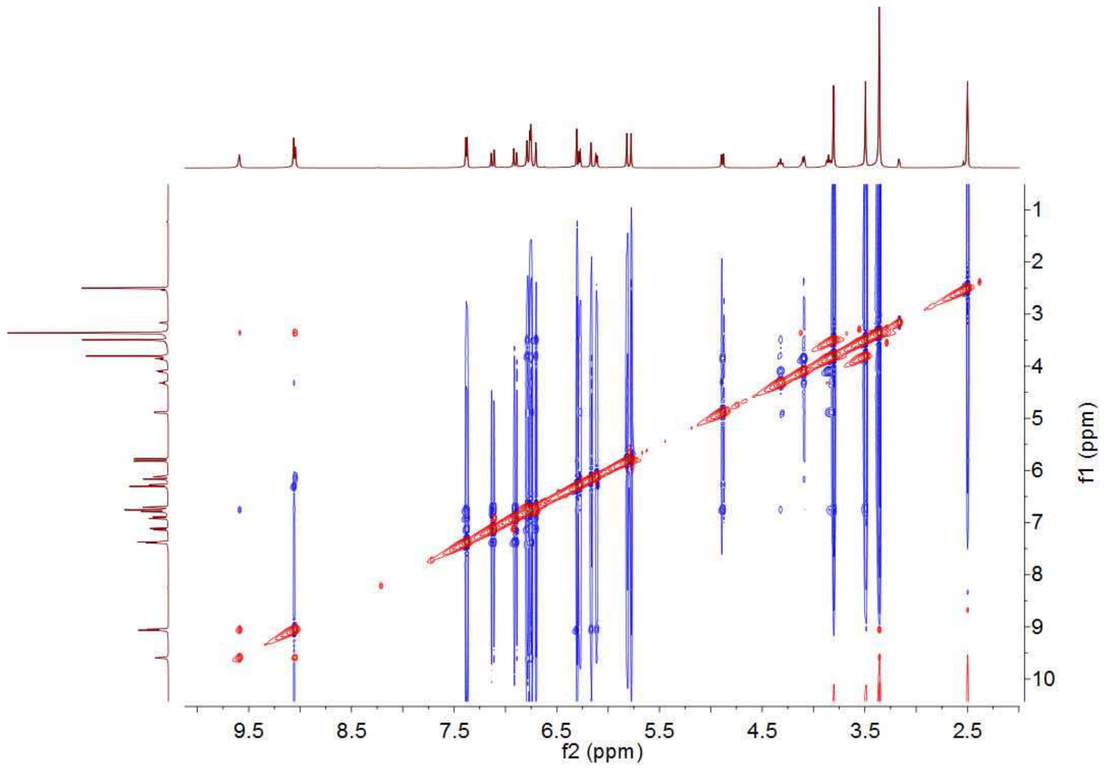

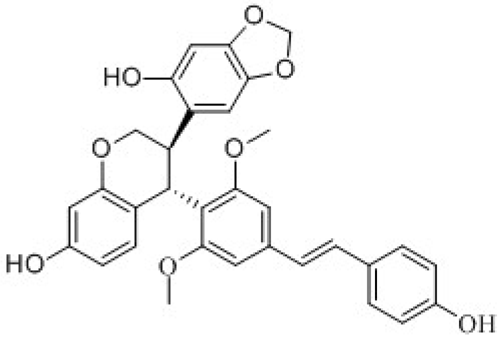

S12. ECD spectrum of Davidiol E (1)

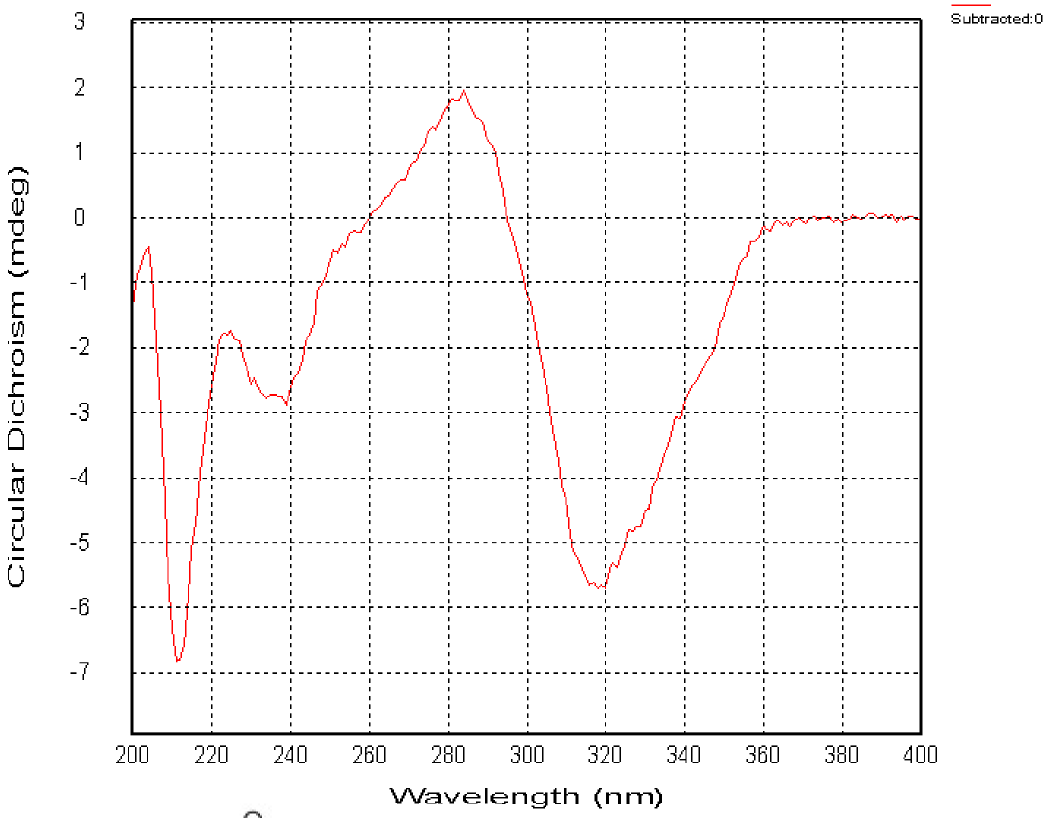

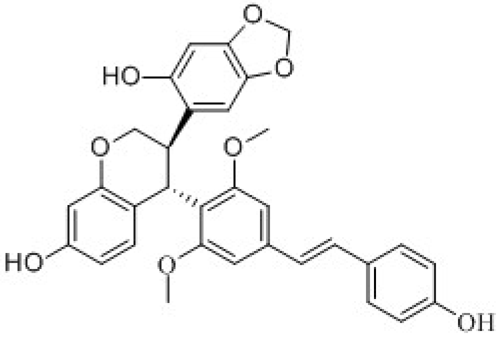

S13. UV spectrum of Davidiol F (2)

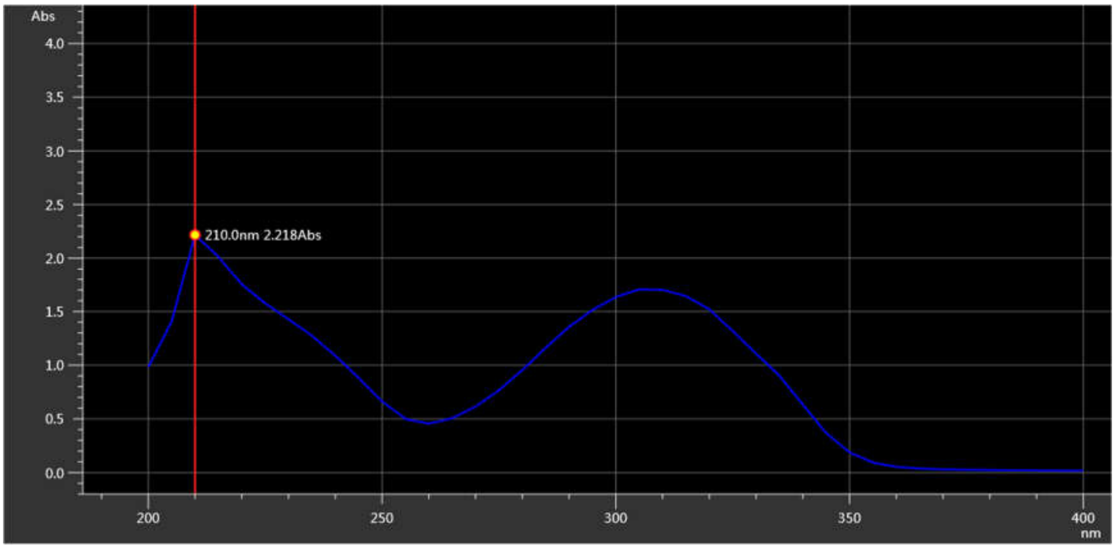

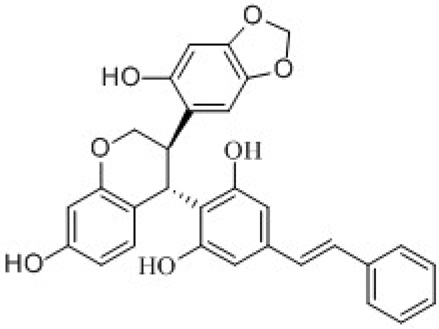

S14. IR spectrum of Davidiol F (2)

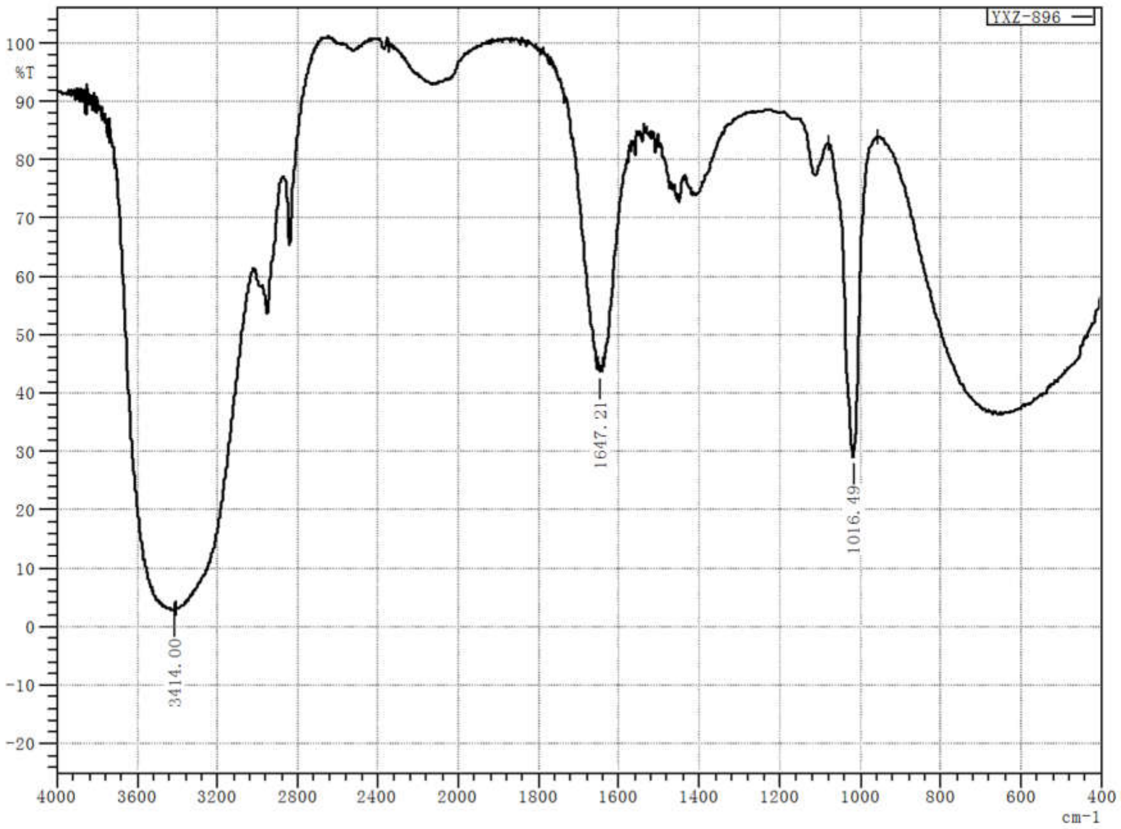

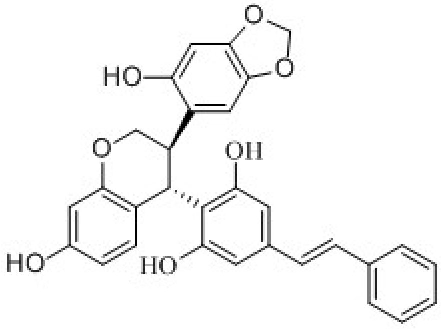

S15. HRESIMS of Davidiol F (2)

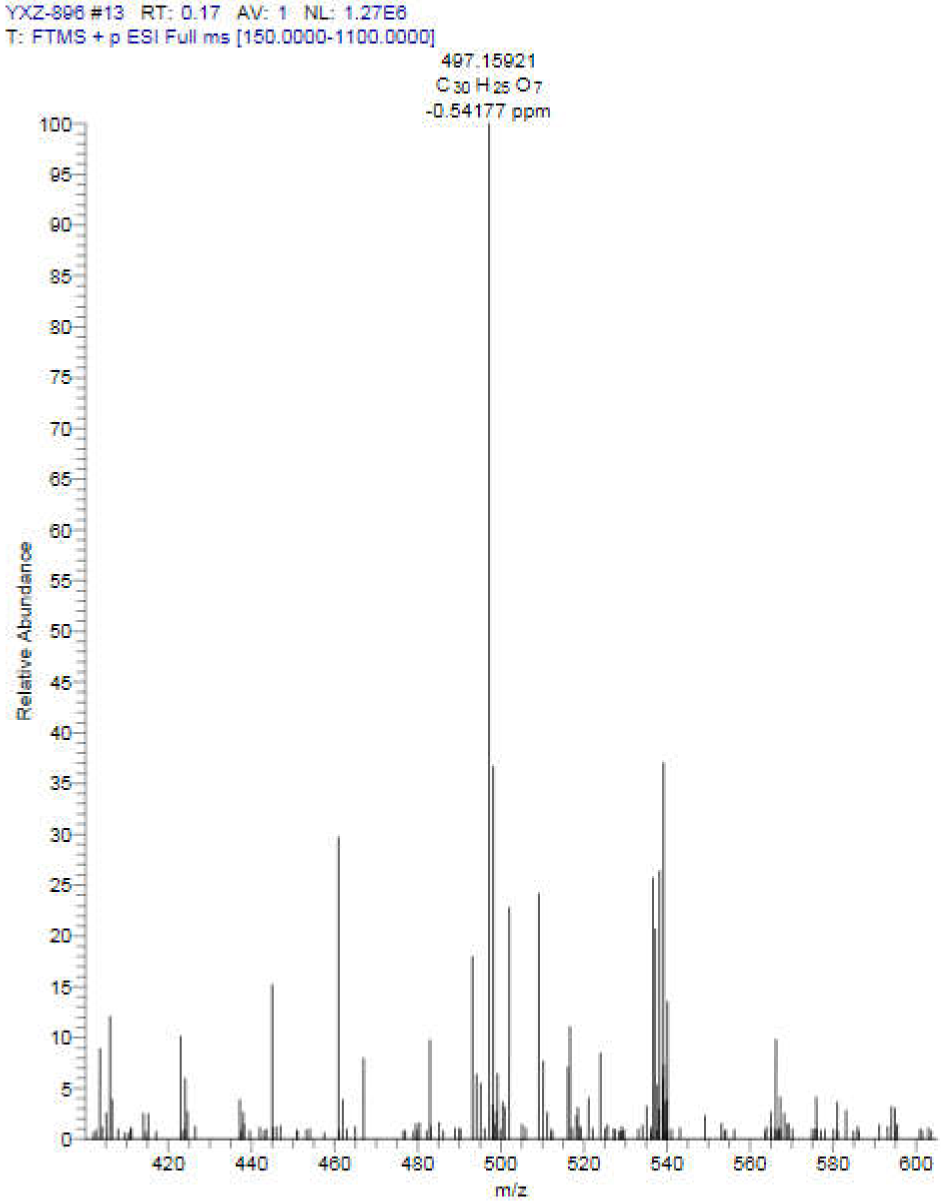

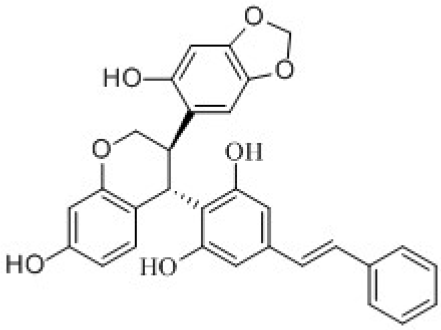

S16. ^{1}H NMR spectrum (600 MHz, DMSO-d_6) of Davidiol F (2)

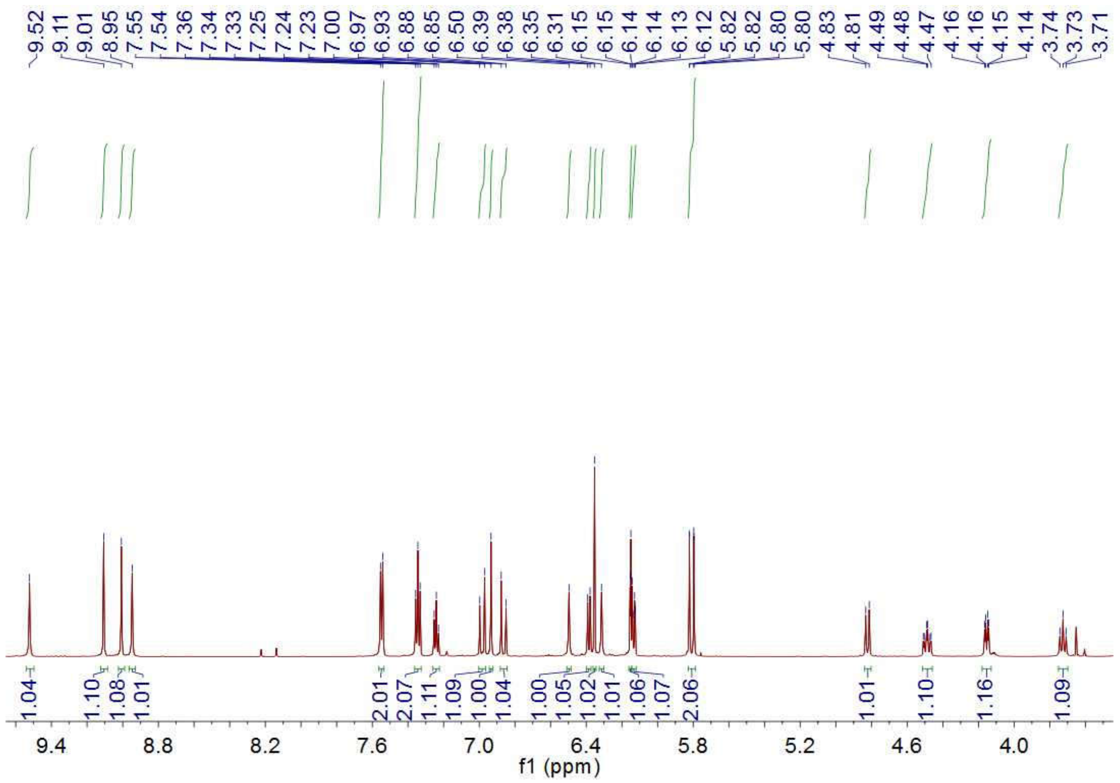

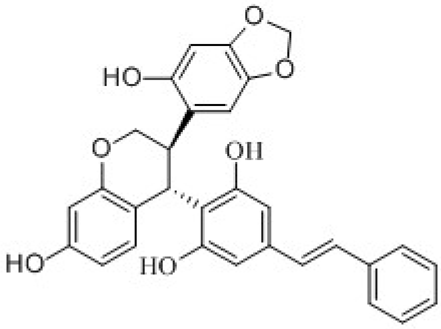

S17. ^{13}C NMR spectrum (150 MHz, DMSO-d_6) of Davidiol F (2)

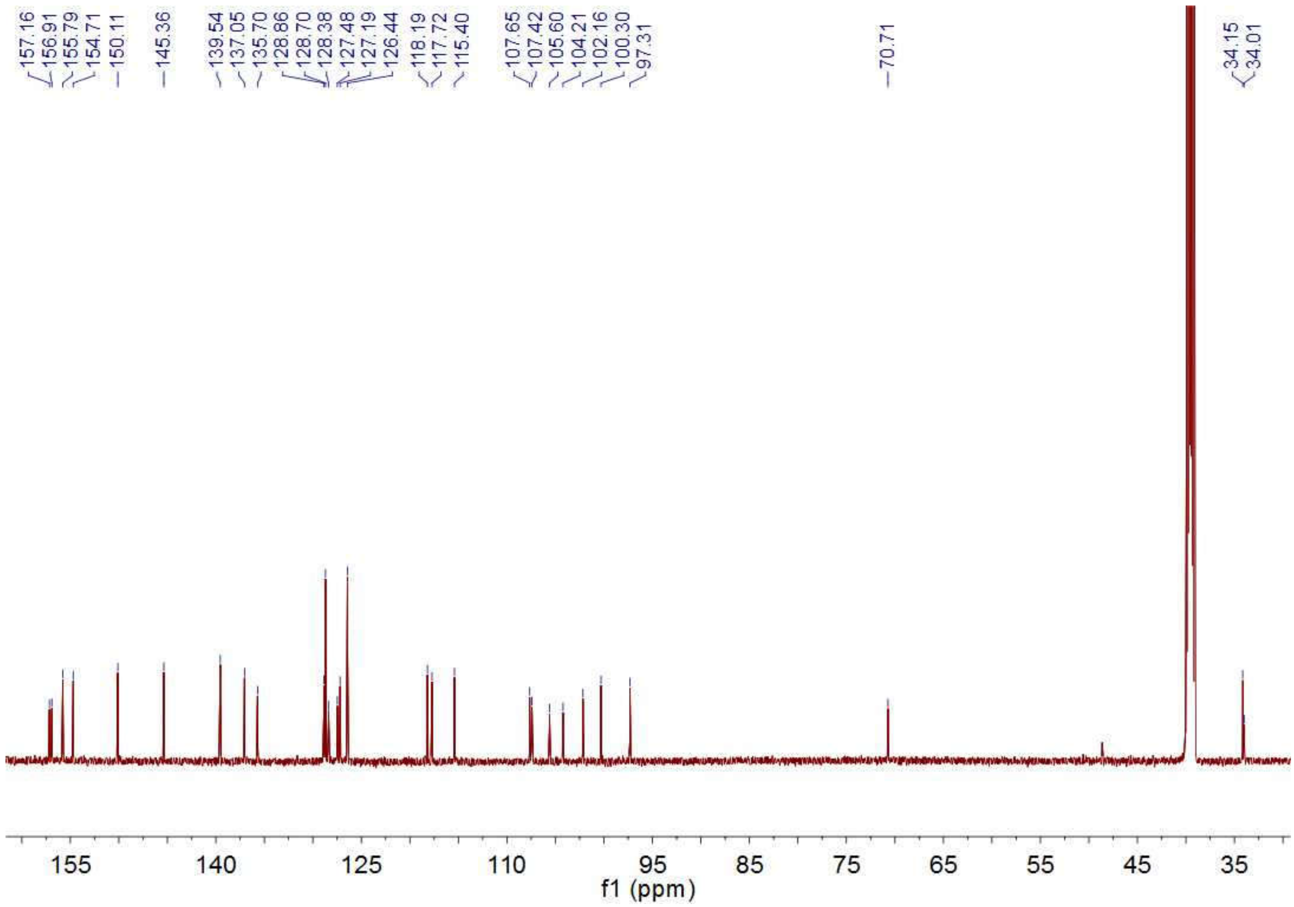

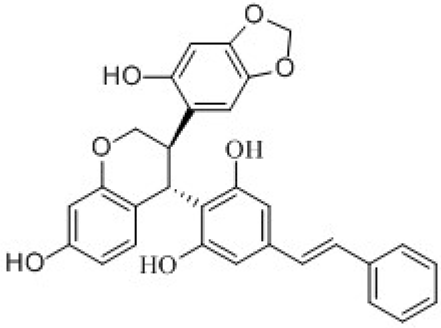

S18. DEPT 135° spectrum (150 MHz, DMSO-d_6) of Davidiol F (2)

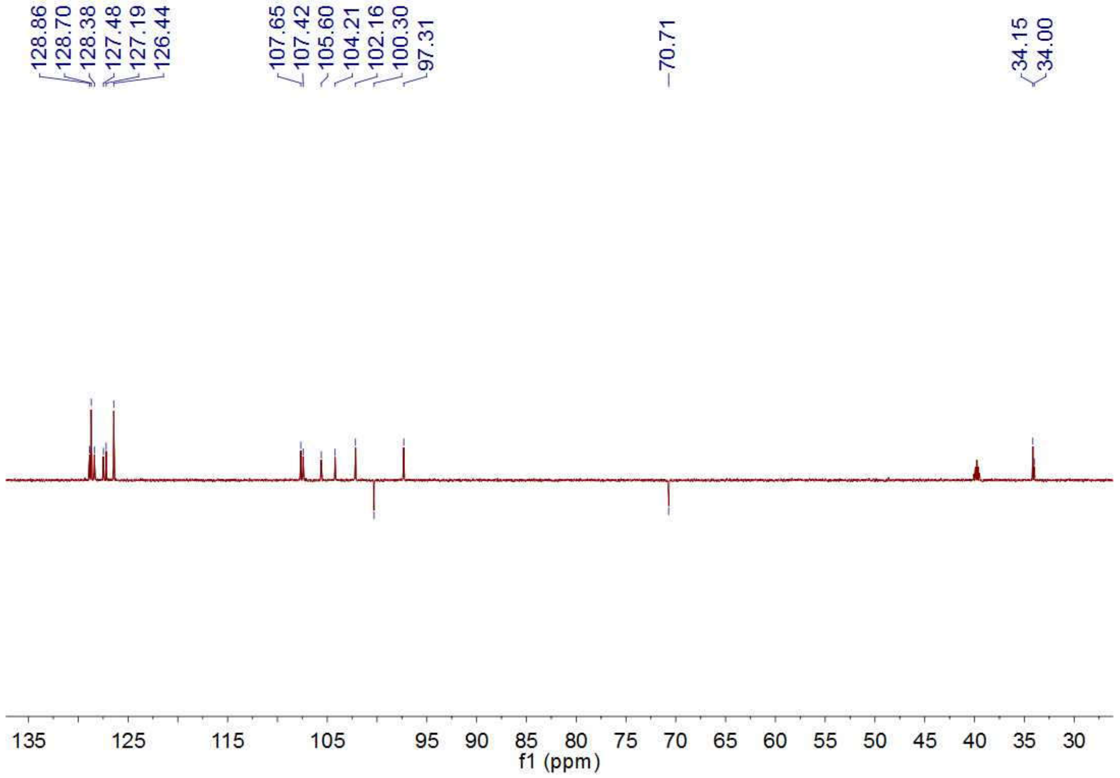

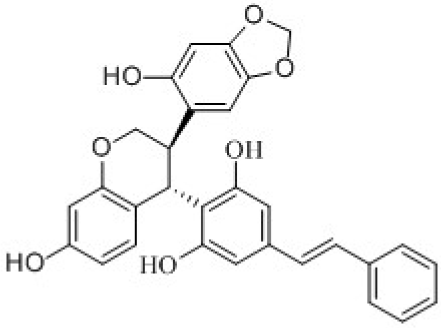

S19. COSY spectrum of Davidiol F (2)

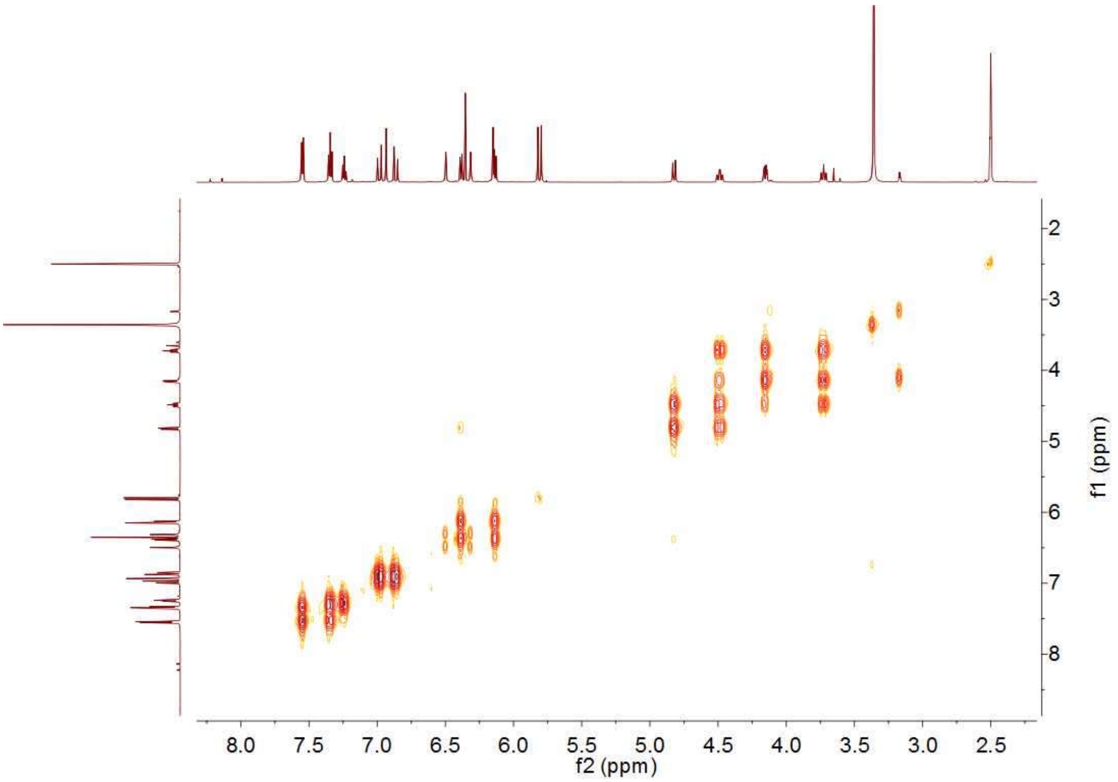

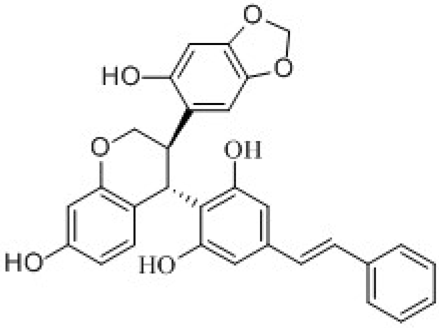

S20. HSQC spectrum of Davidiol F (2)

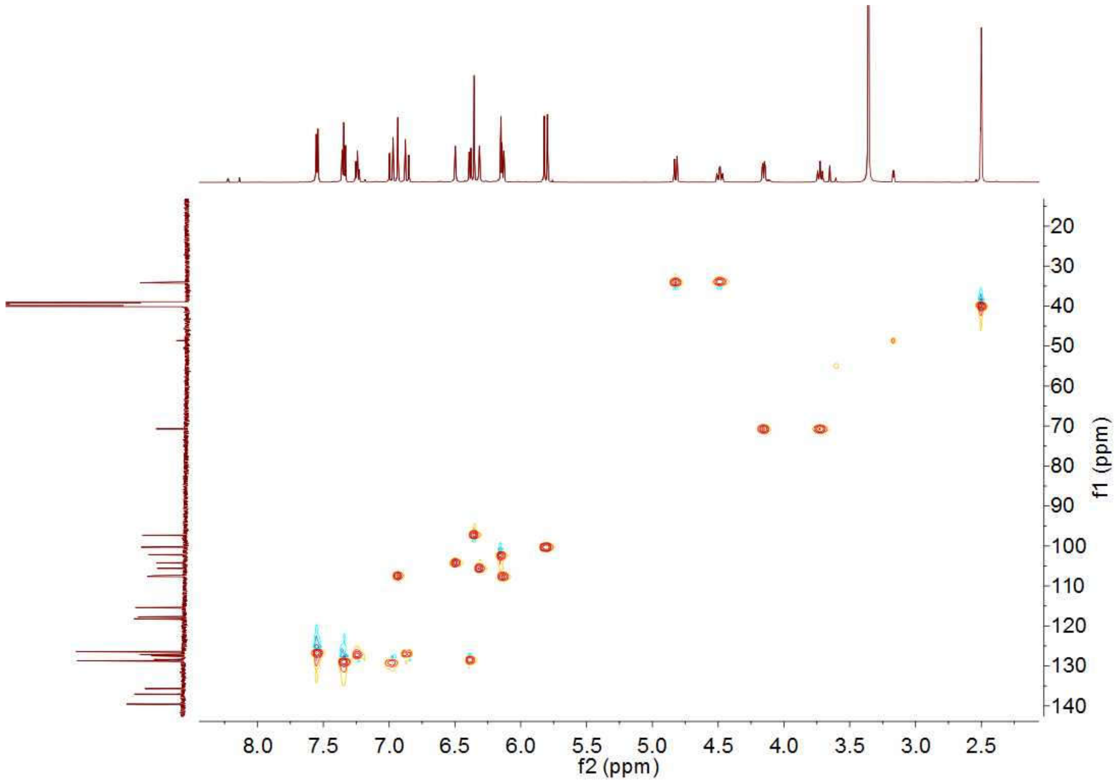

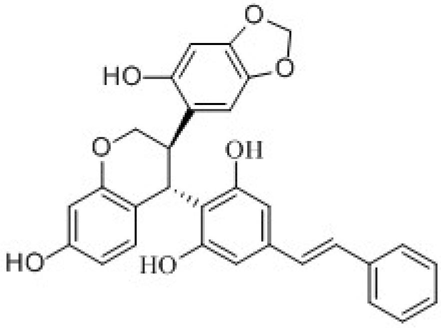

S21. HMBC spectrum of Davidiol F (2)

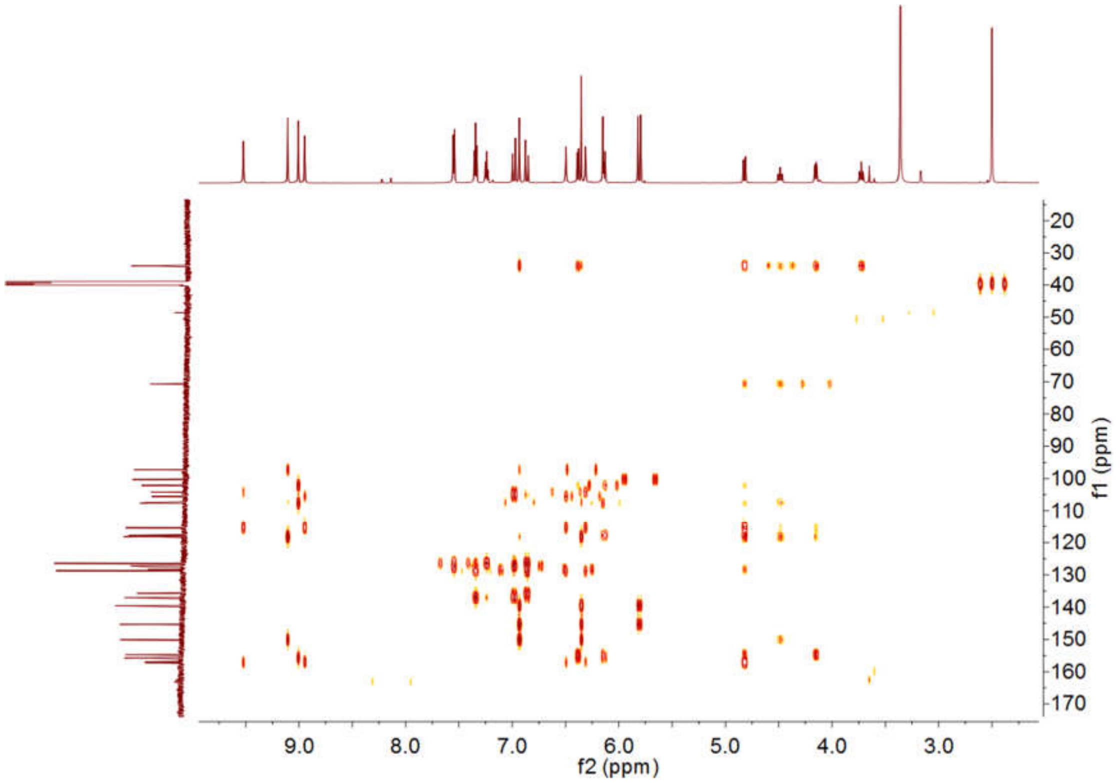

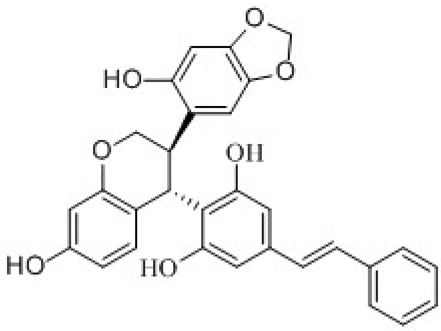

S22. ROESY spectrum of Davidiol F (2)

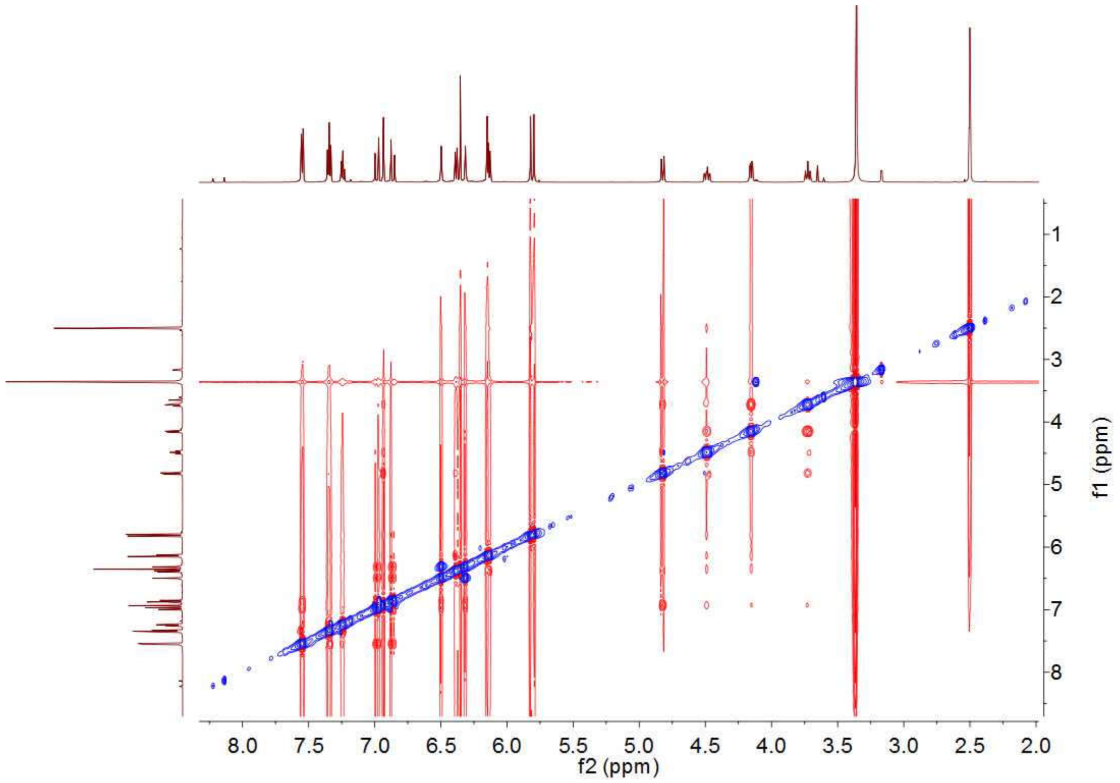

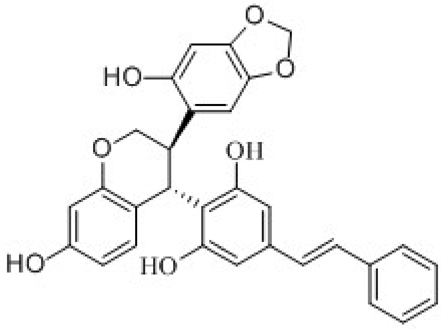

S23. ECD spectrum of Davidiol F (2)

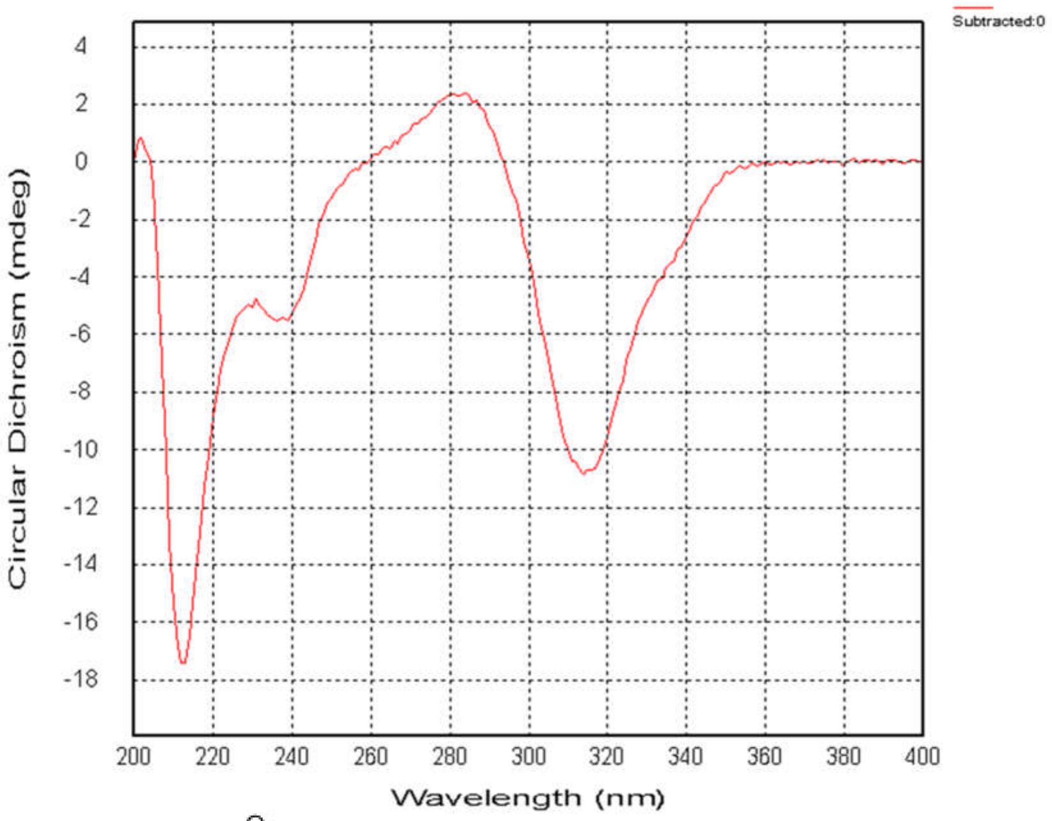

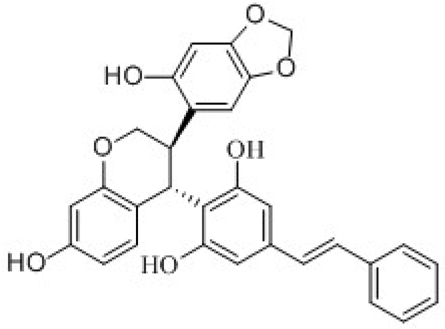

S24. UV spectrum of Davidinin A (3)

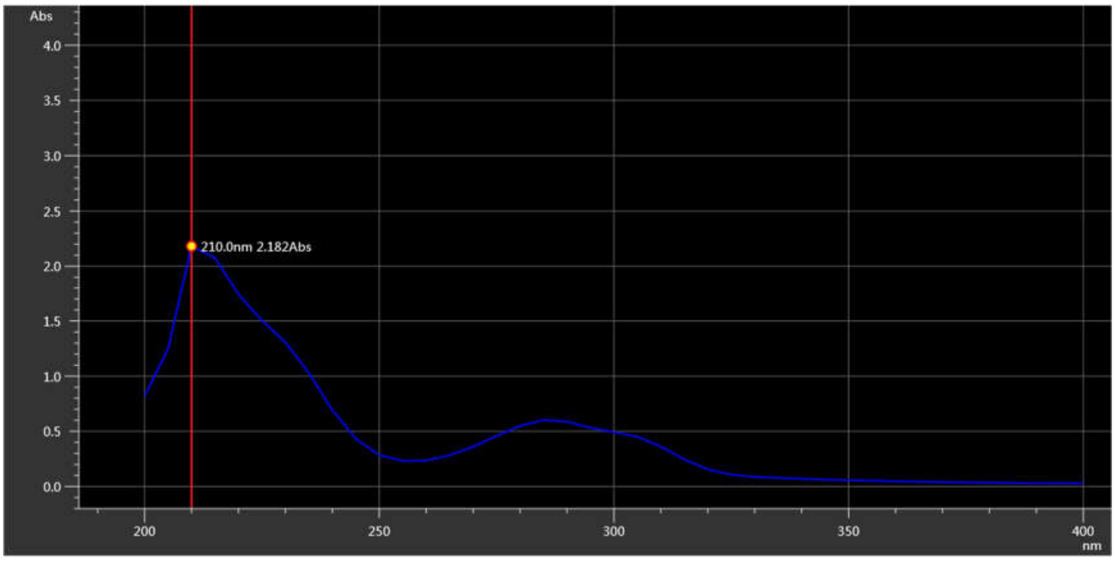

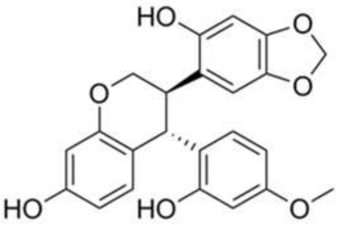

S25. IR spectrum of Davidinin A (3)

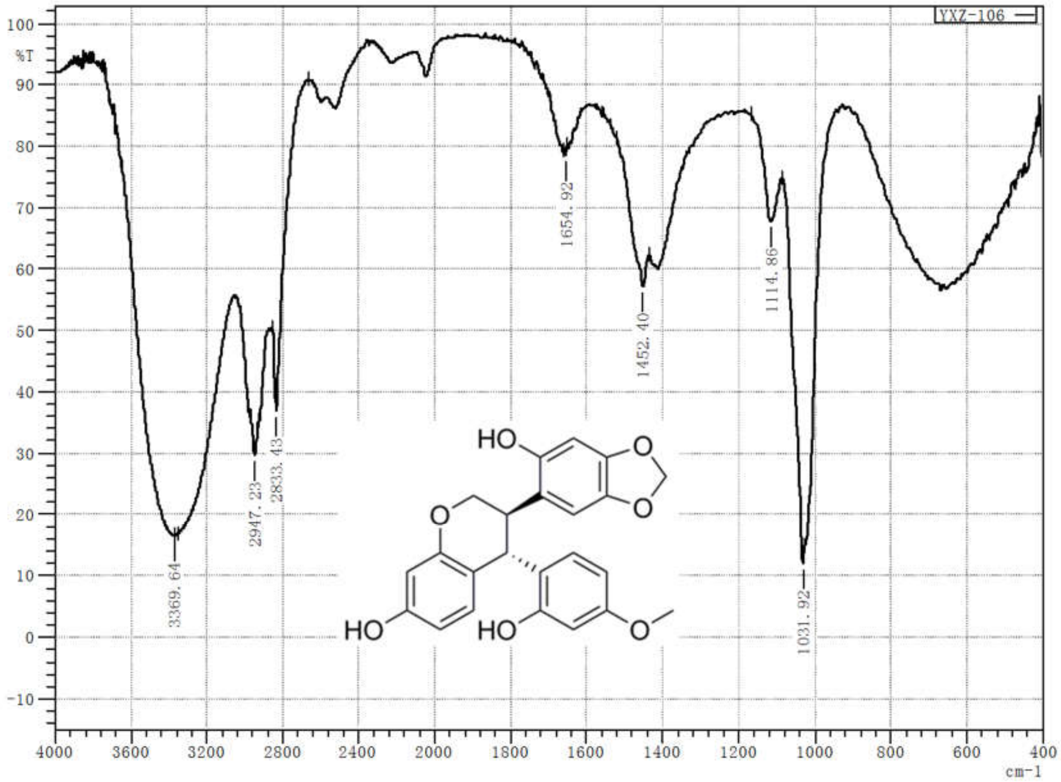

S26. HRESIMS of Davidinin A (3)

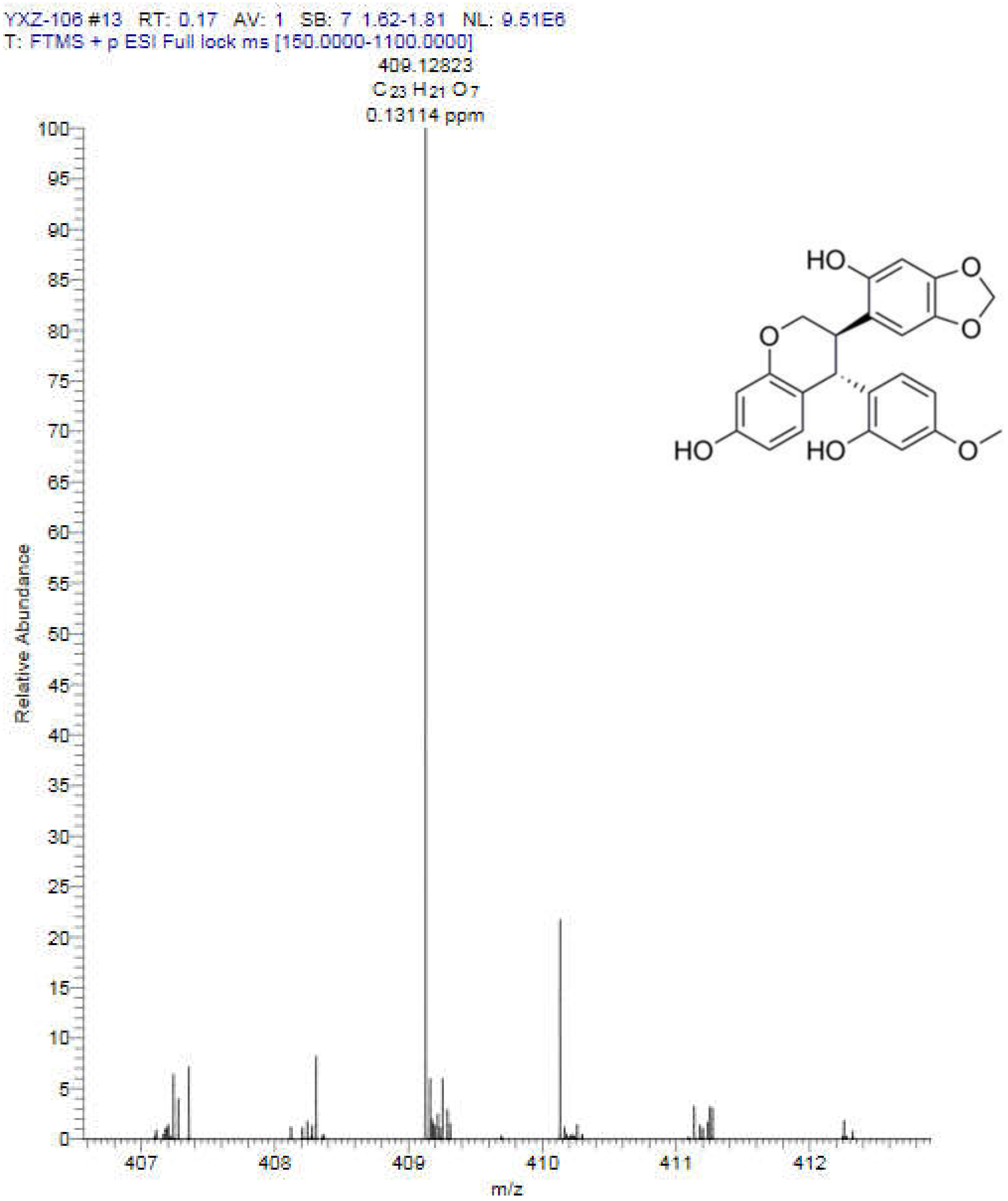

S27. ^{1}H NMR spectrum (600 MHz, MeOH-d_4) of Davidinin A (3)

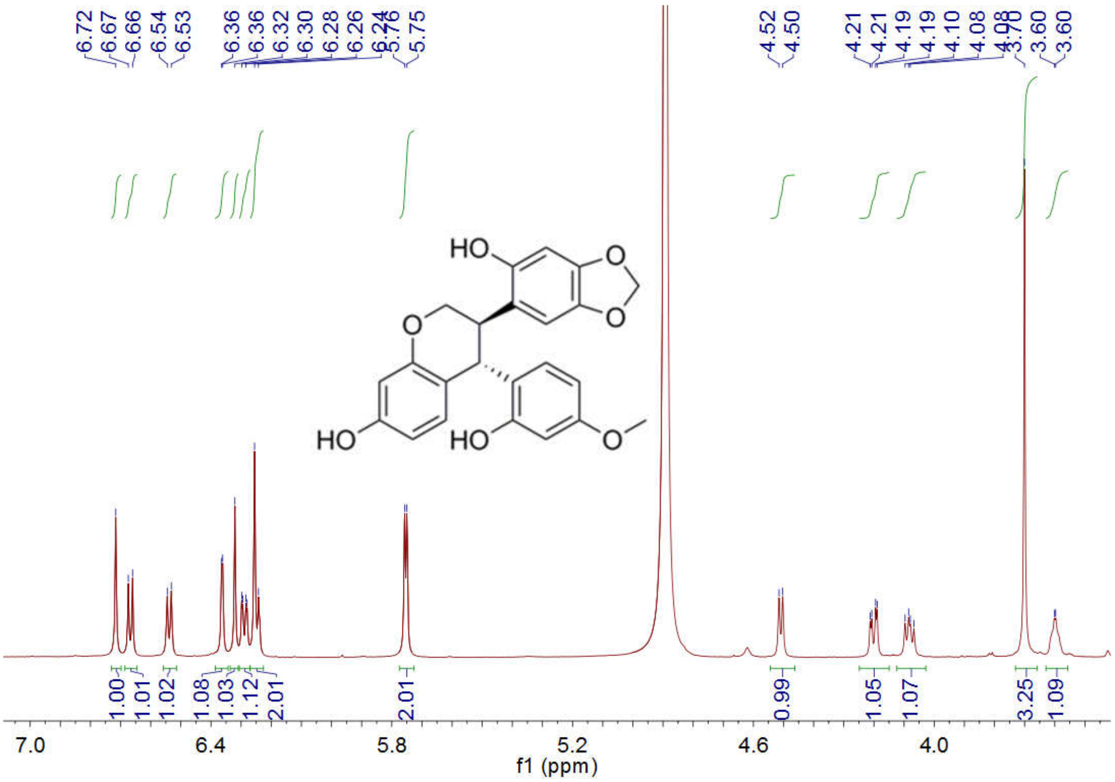

S28. ^{13}C NMR spectrum (150 MHz, MeOH-d_4) of Davidinin A (3)

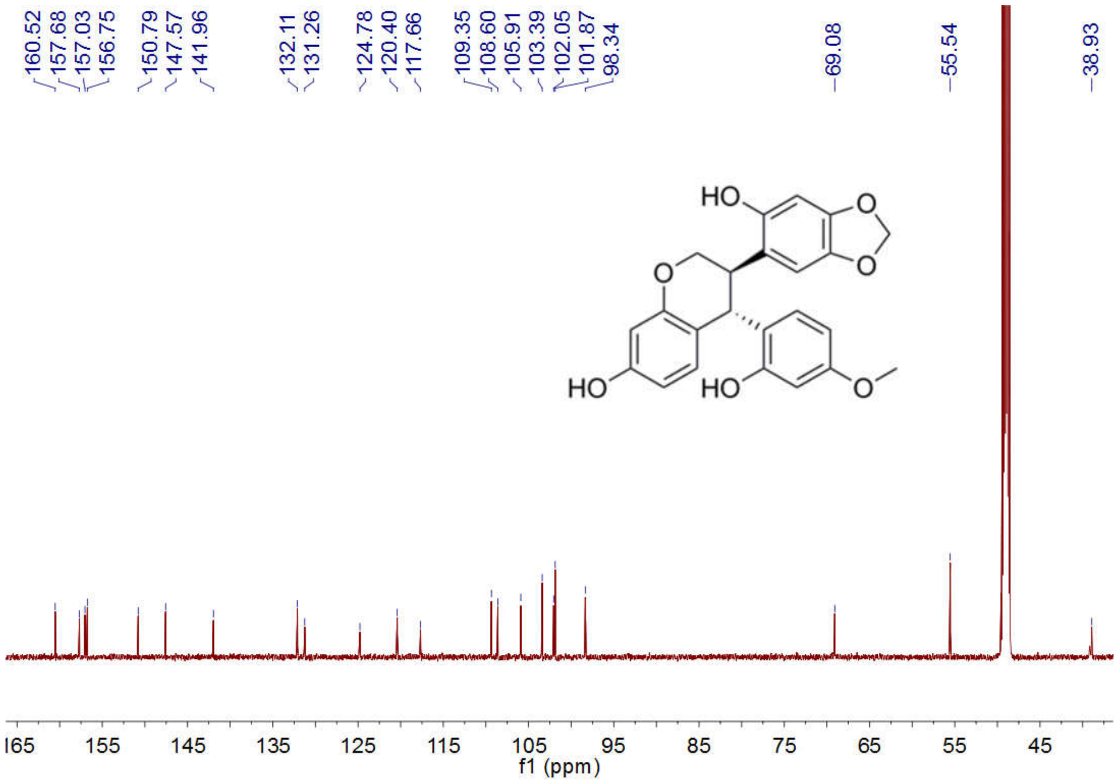

S29. DEPT 135º spectrum (150 MHz, MeOH-d_4) of Davidinin A (3)

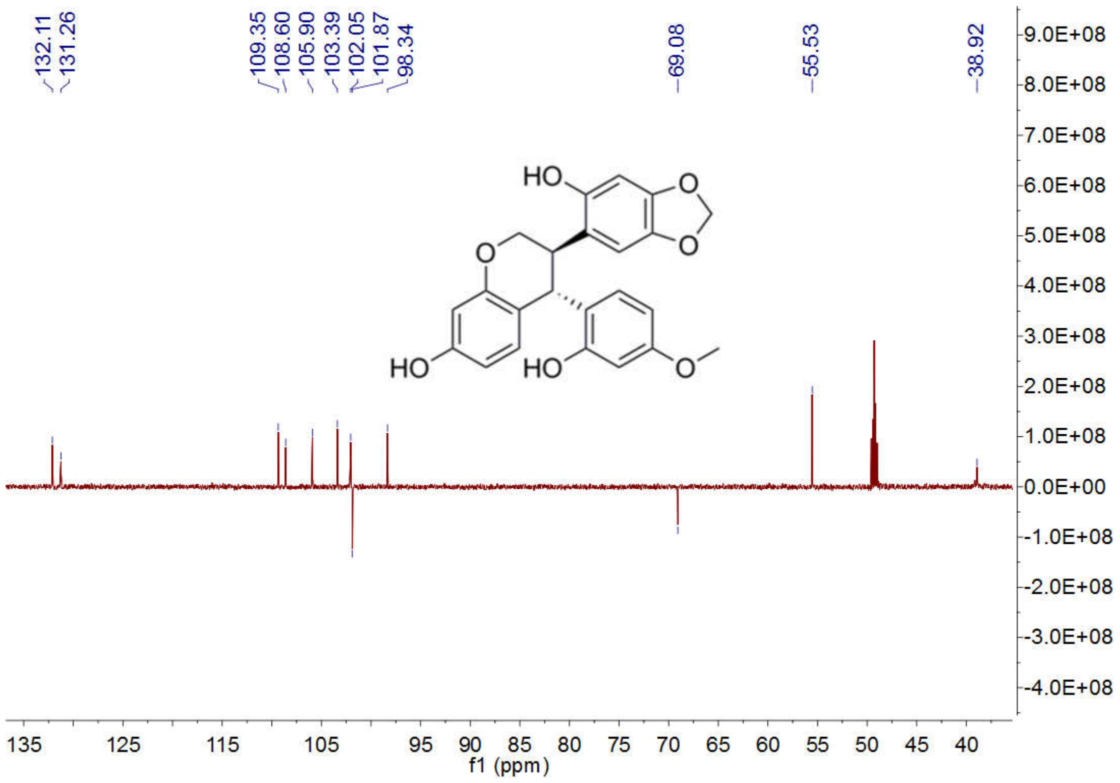

S30. COSY spectrum of Davidinin A (3)

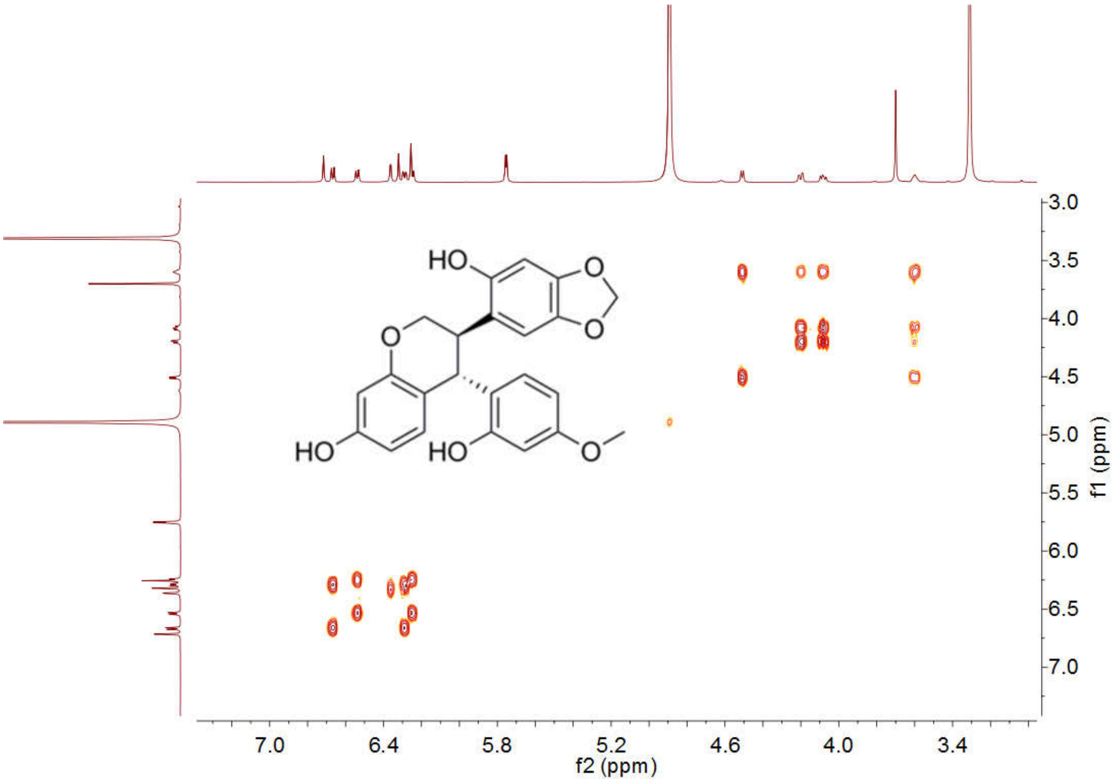

S31. HSQC spectrum of Davidinin A (3)

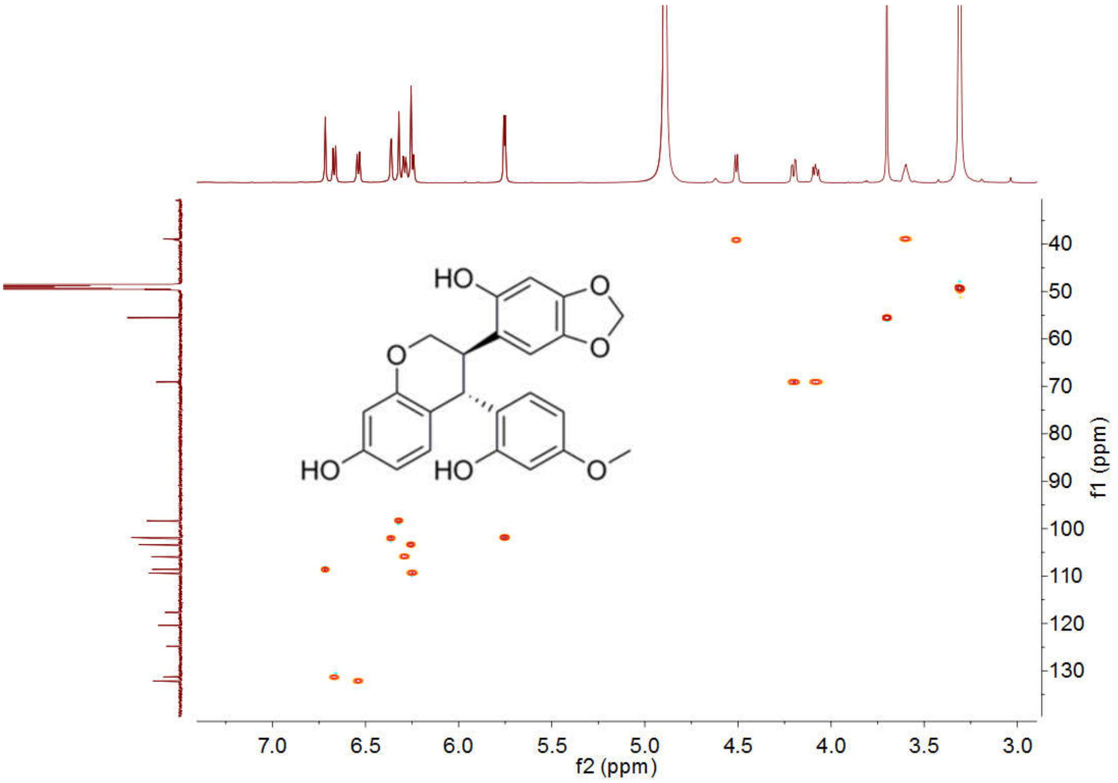

S32. HMBC spectrum of Davidinin A (3)

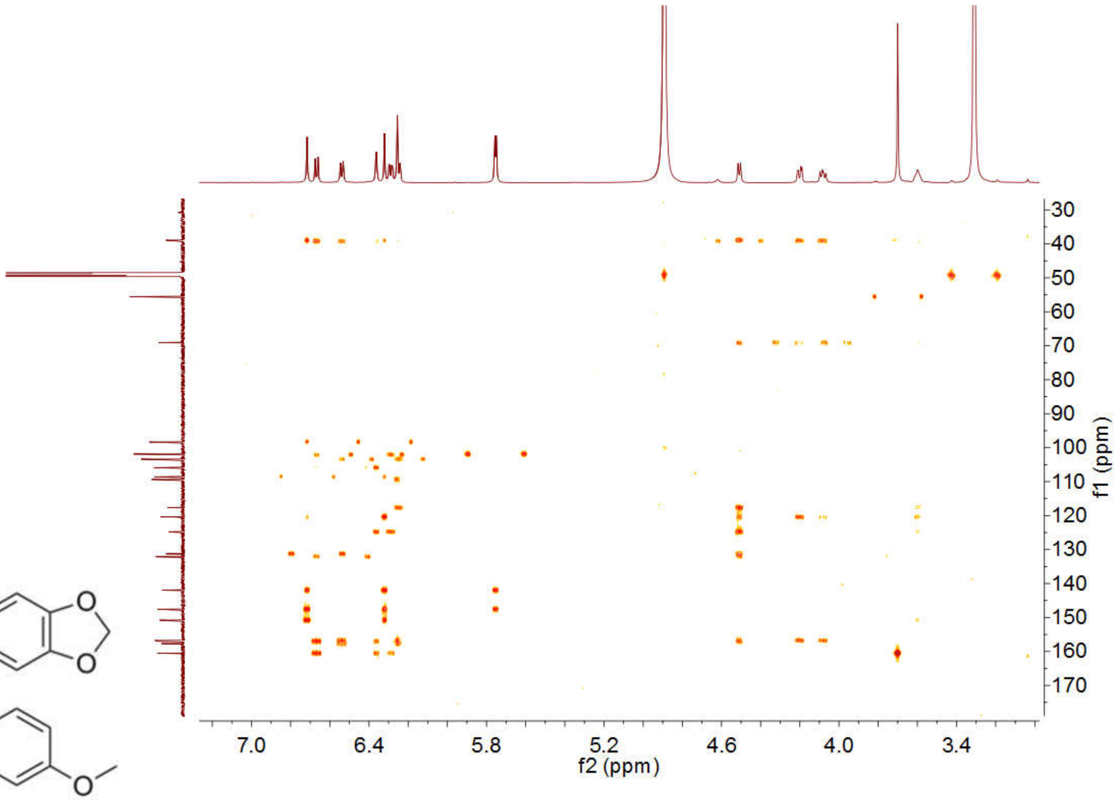

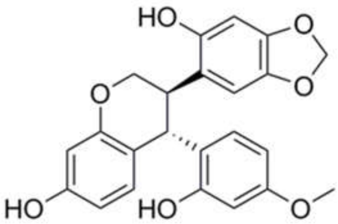

S33. ROESY spectrum of Davidinin A (3)

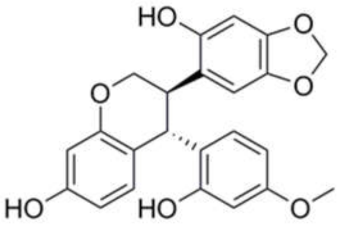

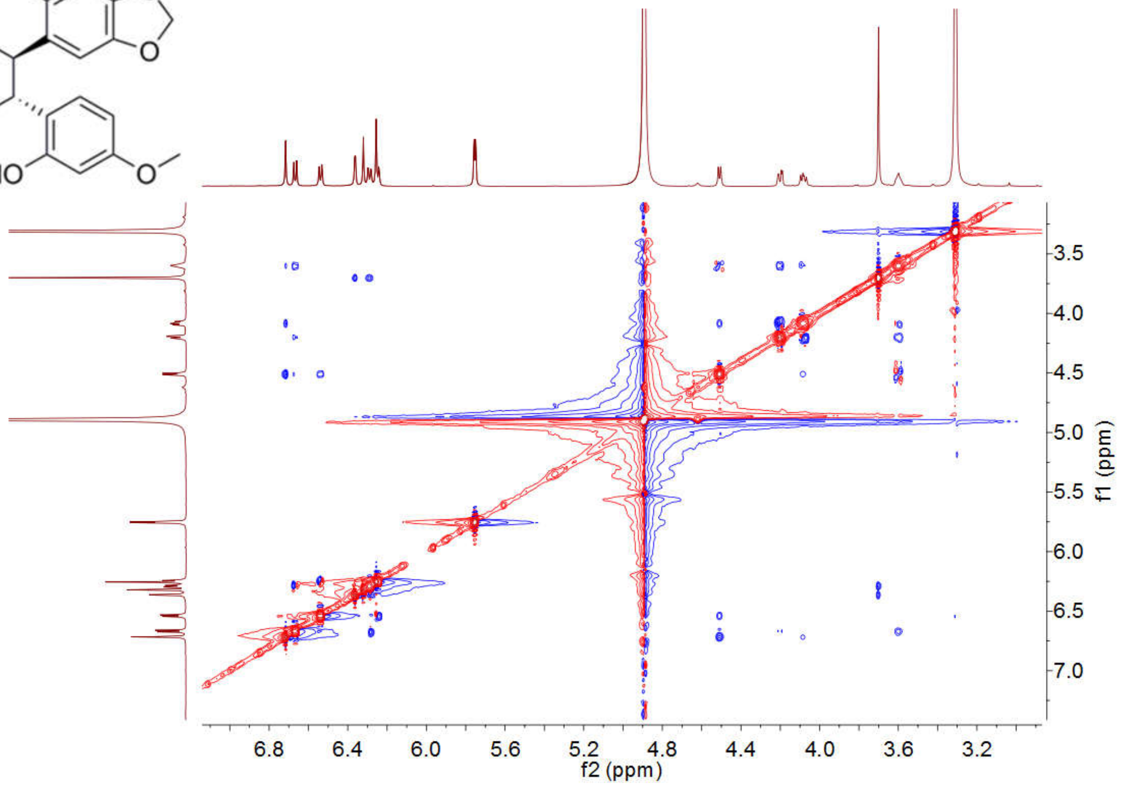

S34. ECD spectrum of Davidinin A (3)

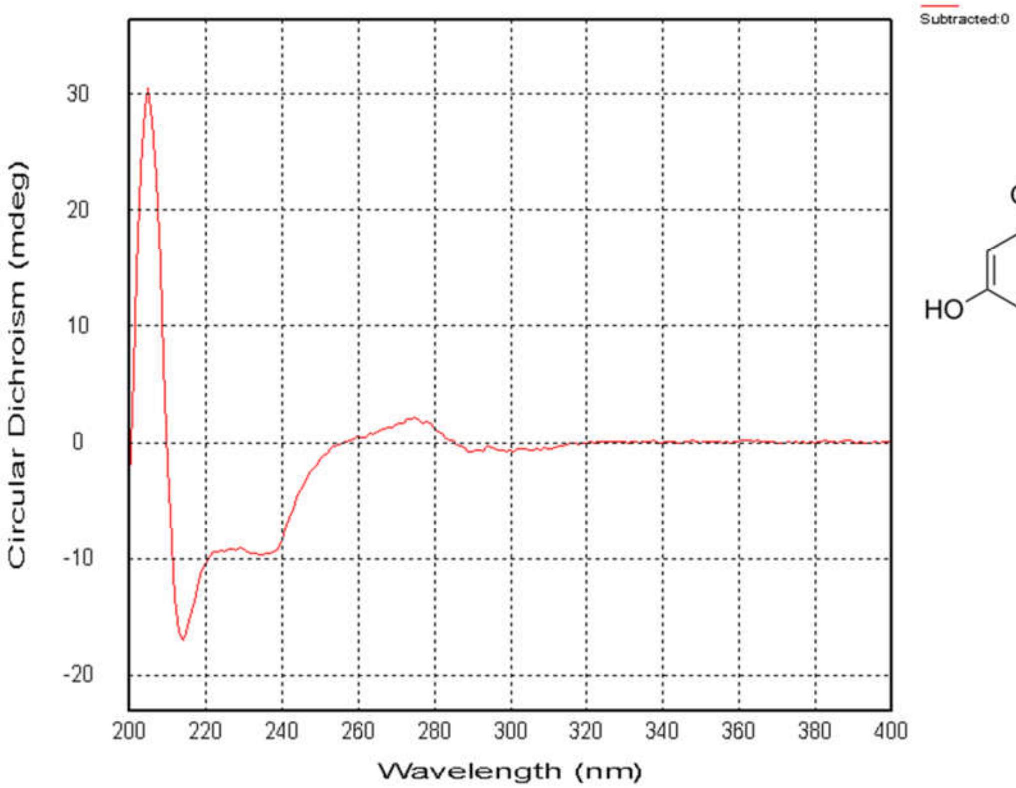

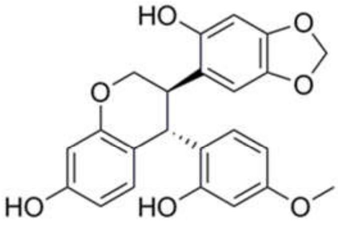

S35. UV spectrum of Shandougenine C (4)

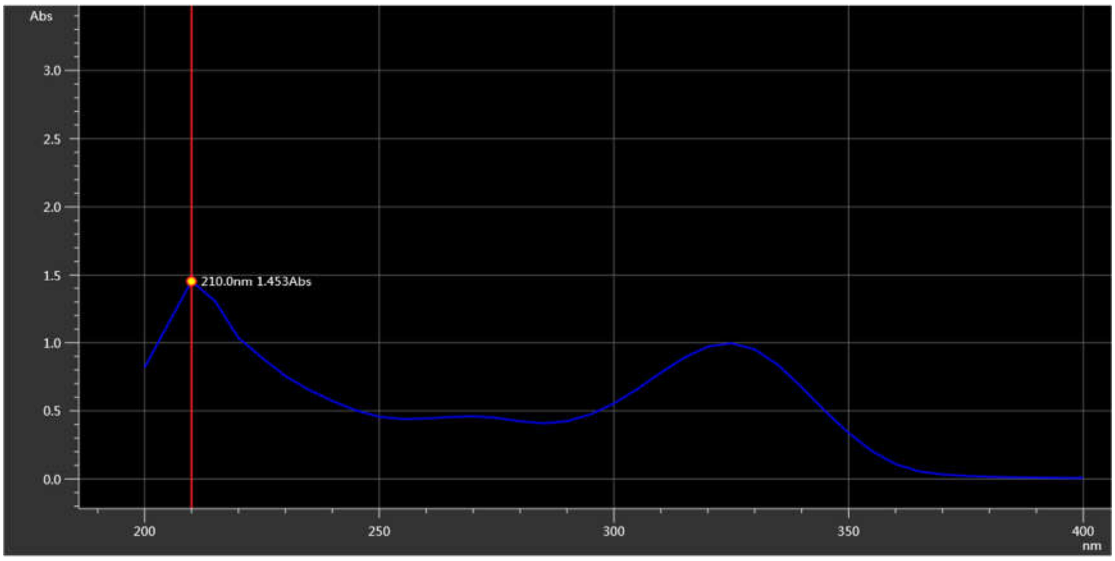

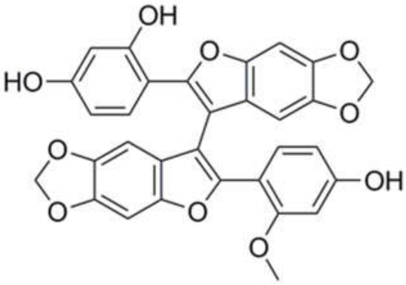

S36. IR spectrum of Shandougenine C (4)

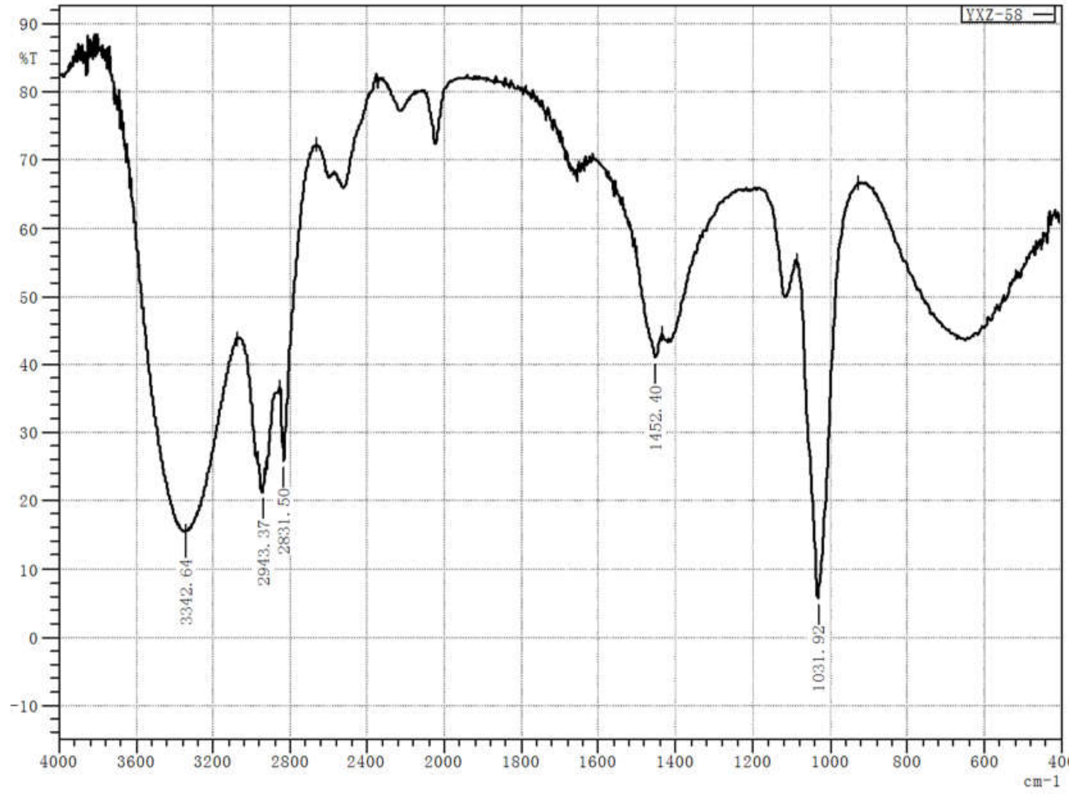

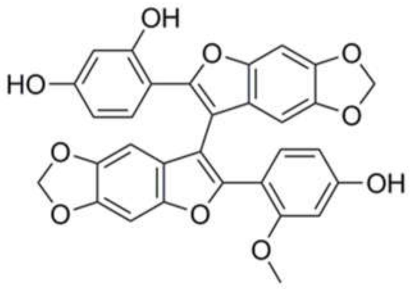

S37. HRESIMS of Shandougenine C (4)

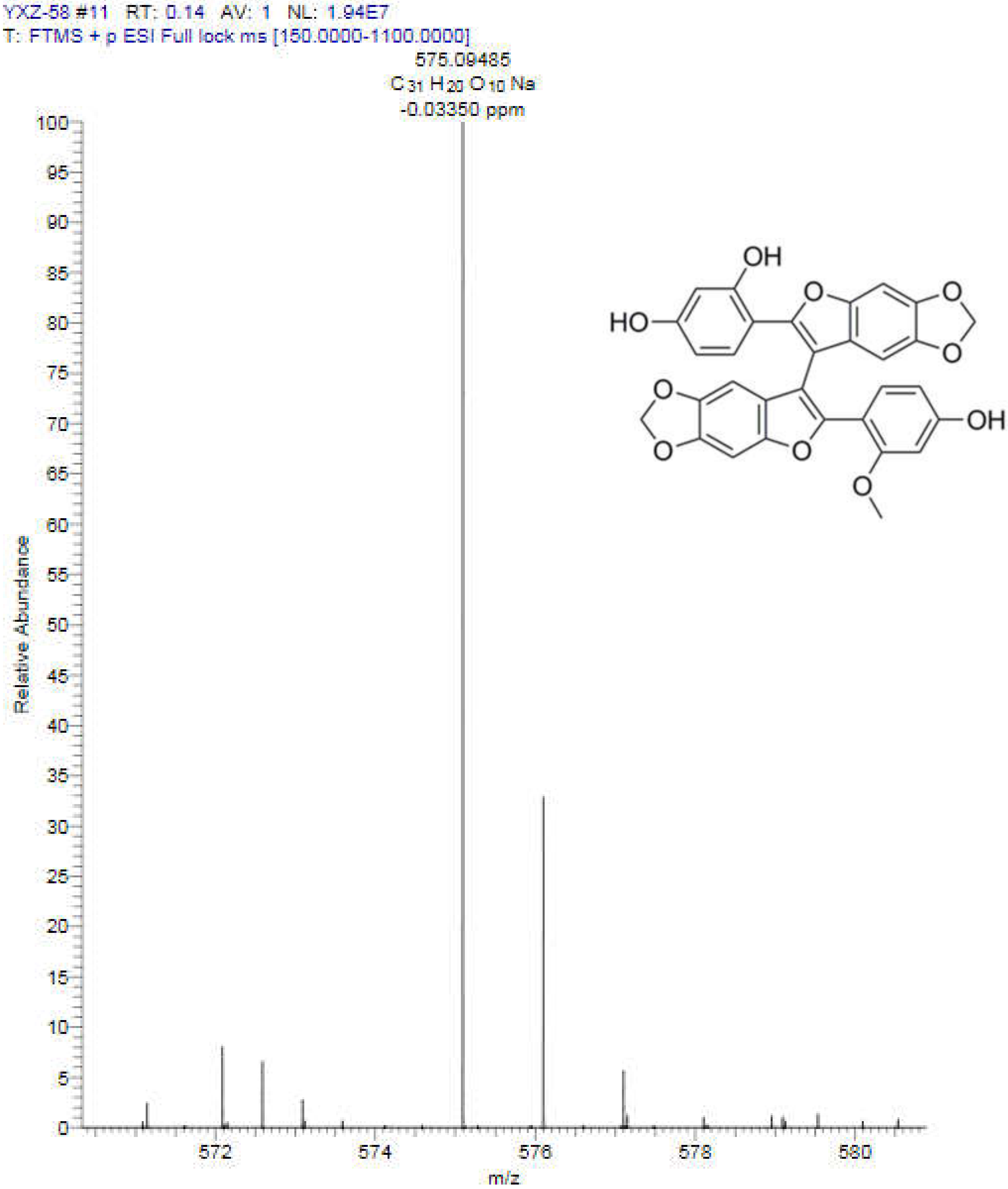

S38. ^{1}H NMR spectrum (600 MHz, MeOH-d_4) of Shandougenine C (4)

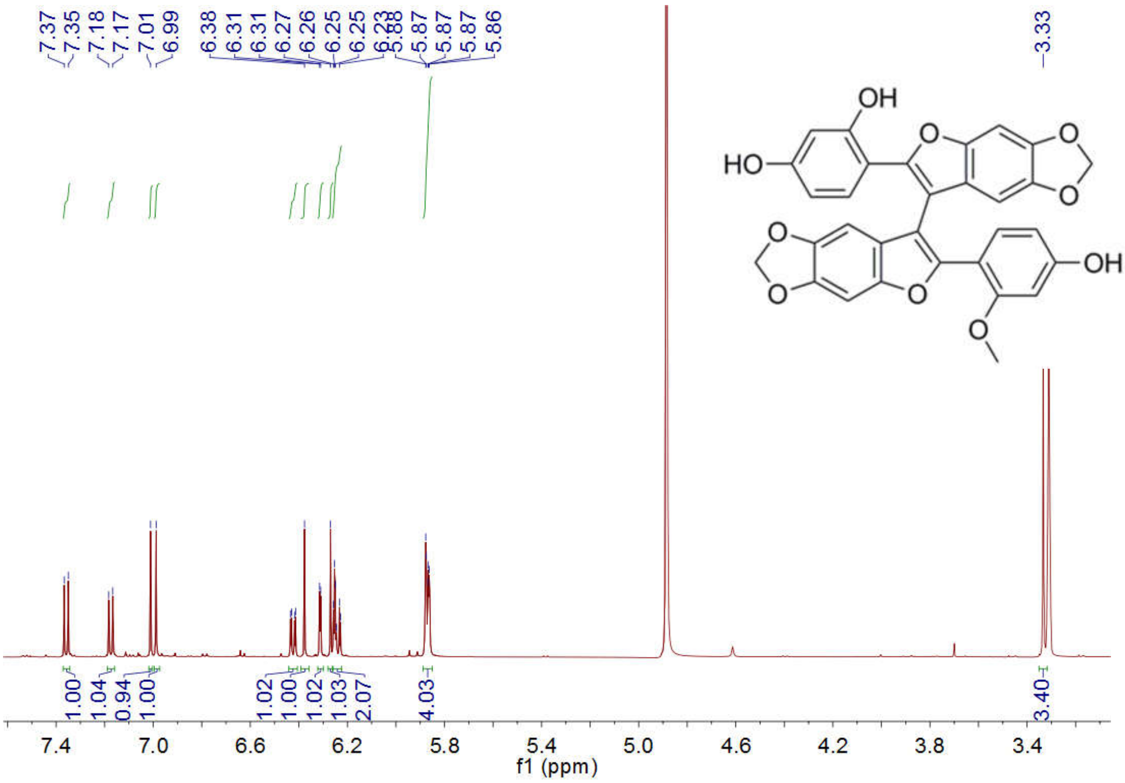

S39. ^{13}C NMR spectrum (150 MHz, MeOH-d_4) of Shandougenine C (4)

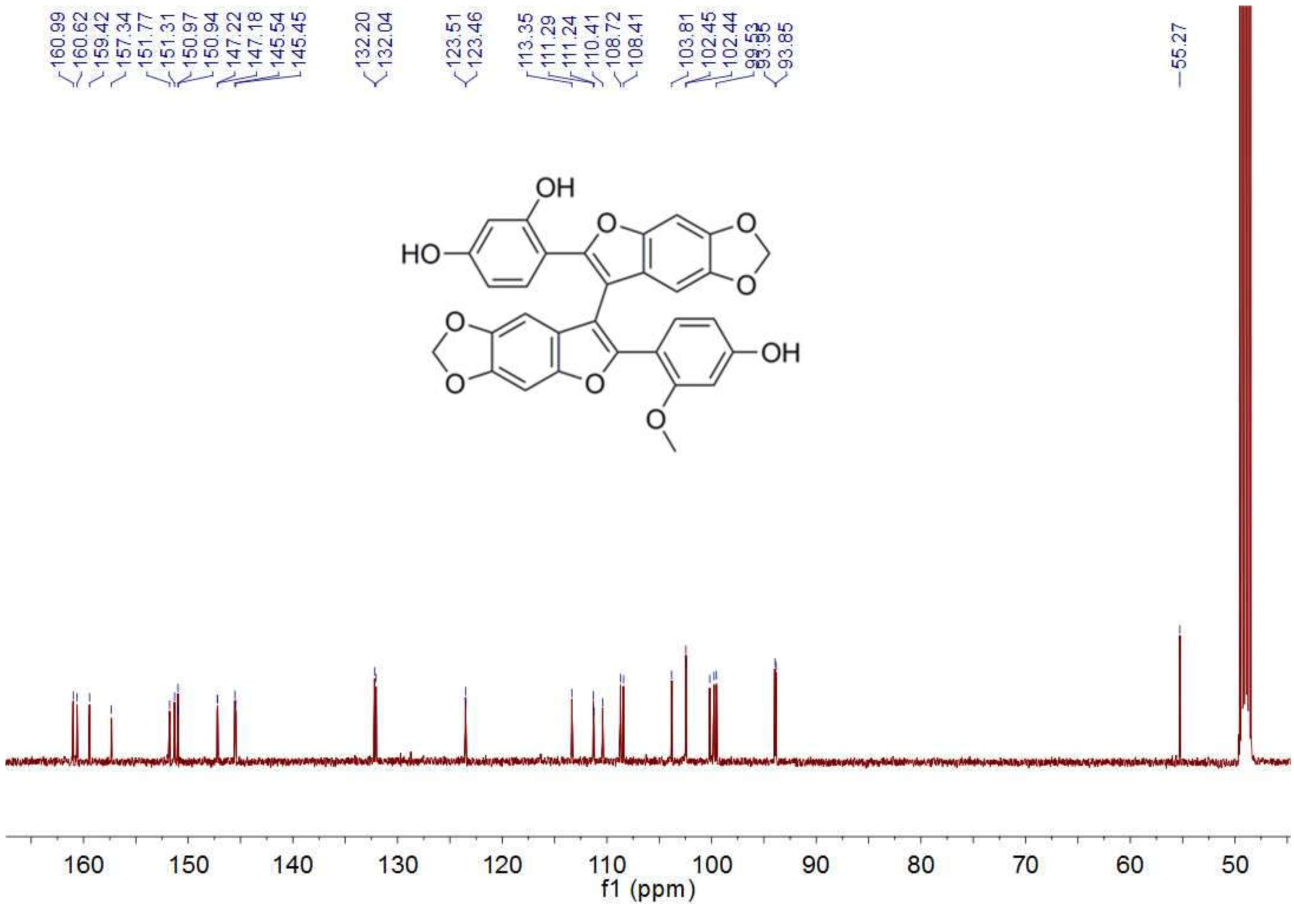

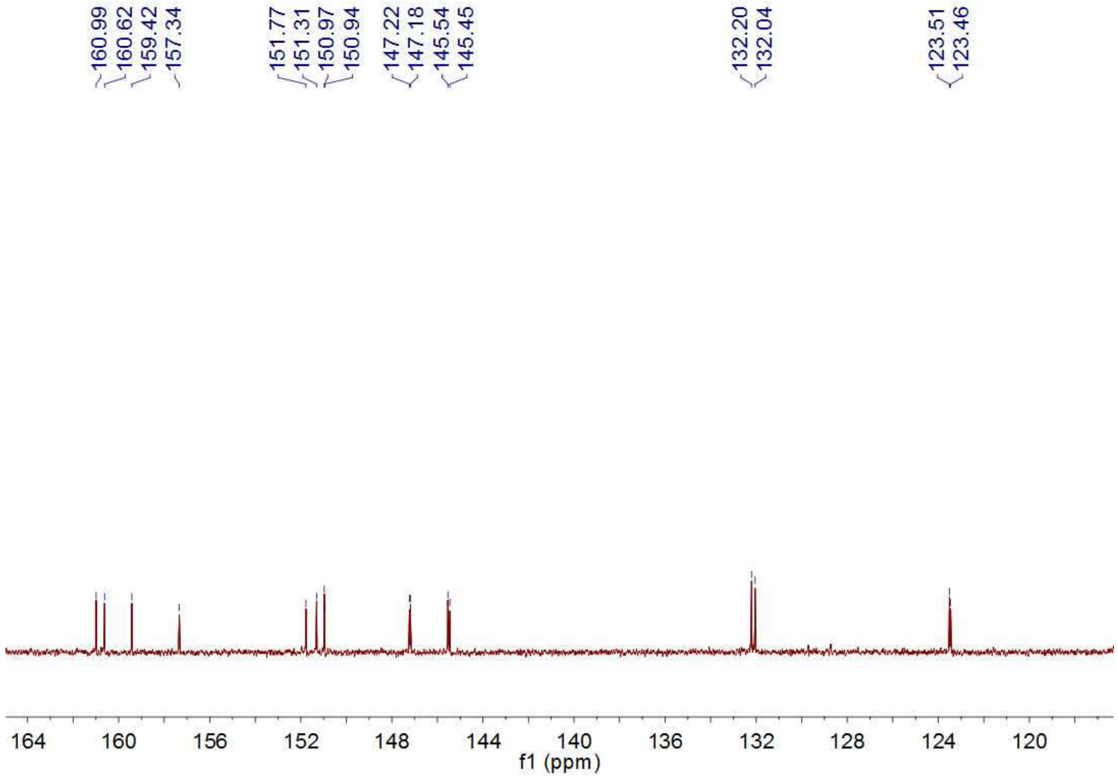

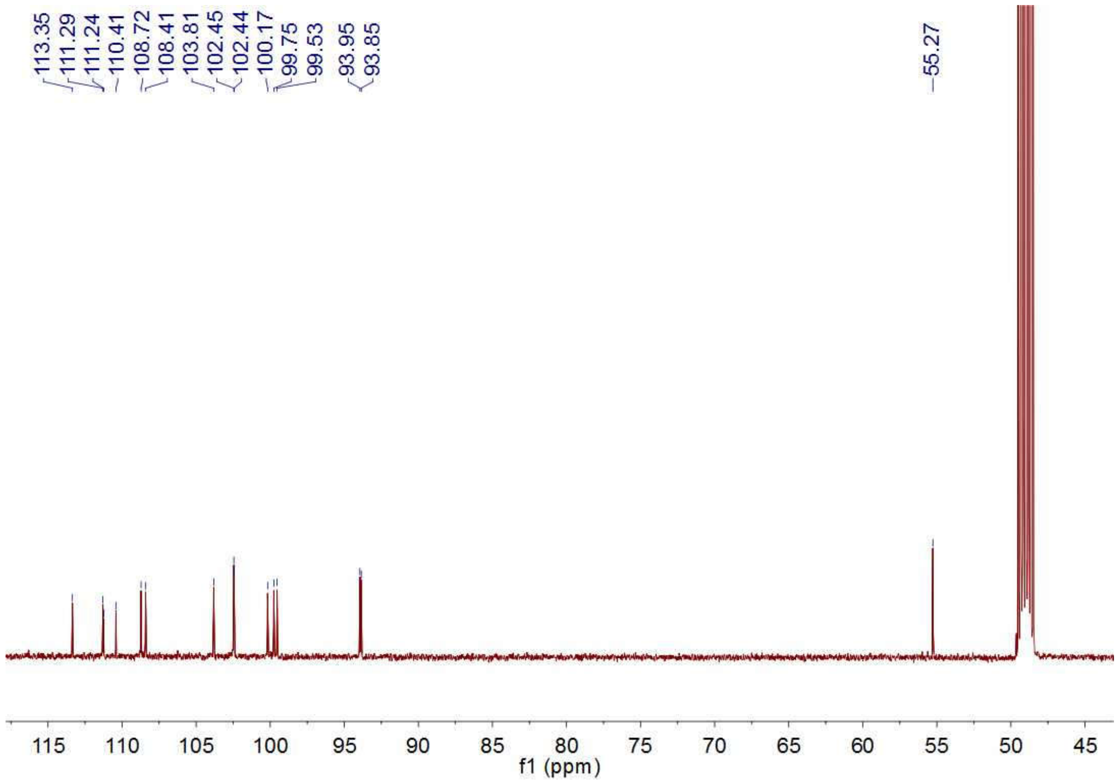
113.35
111.29
111.24
110.41
108.72
108.41
103.81
102.45
102.44
100.17
99.75
99.53
93.95
93.85
55.27
115 110 105 100 95 90 85 80 75 70 65 60 55 50 45
f1 (ppm)

S40. DEPT 135º spectrum (150 MHz, MeOH-d_4) of Shandougenine C (4)

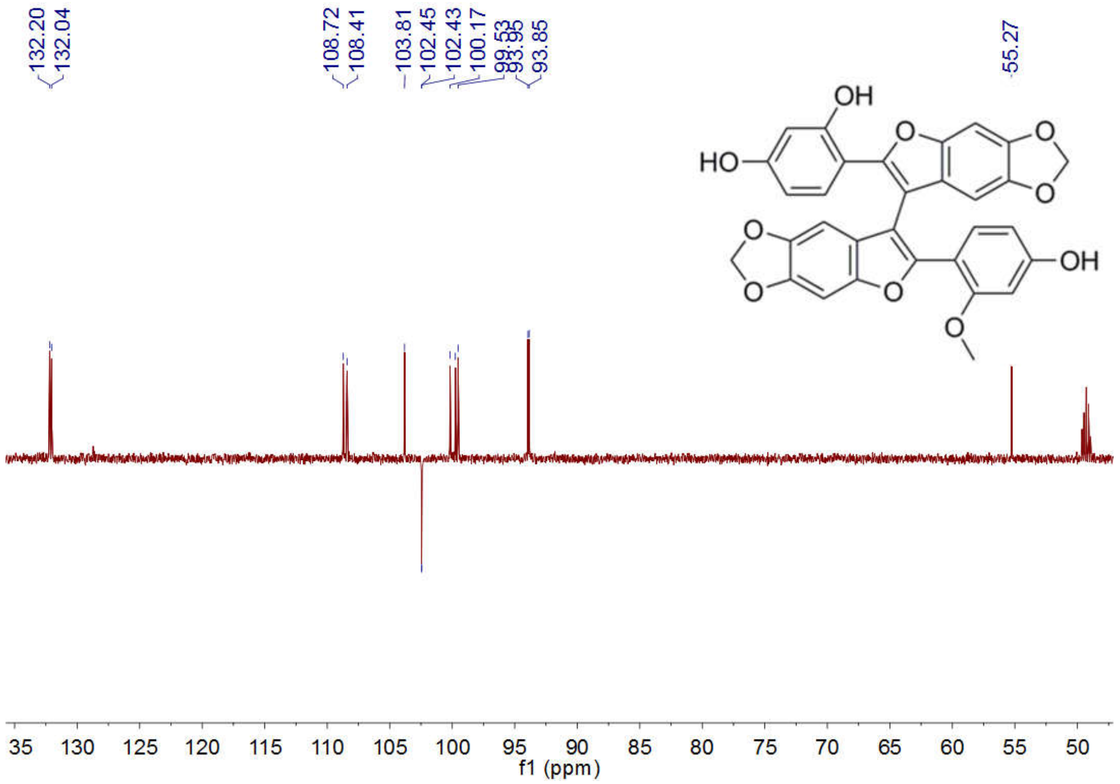

S41. COSY spectrum of Shandougenine C (4)

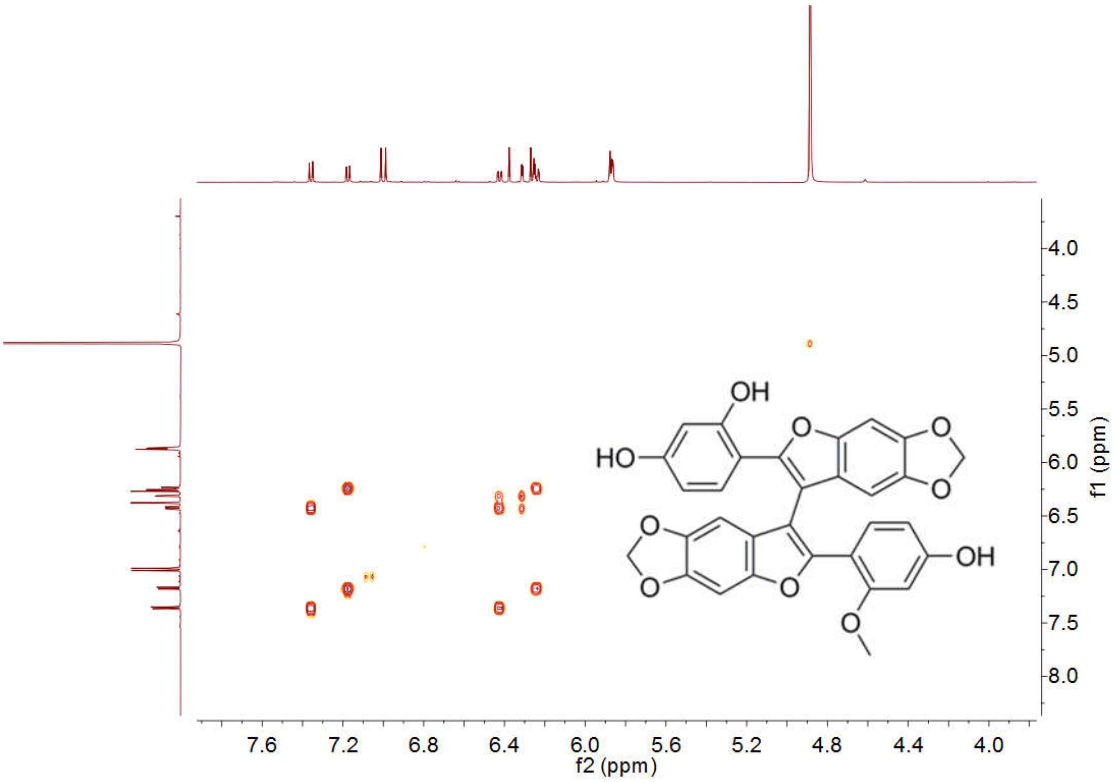

S42. HSQC spectrum of Shandougenine C (4)

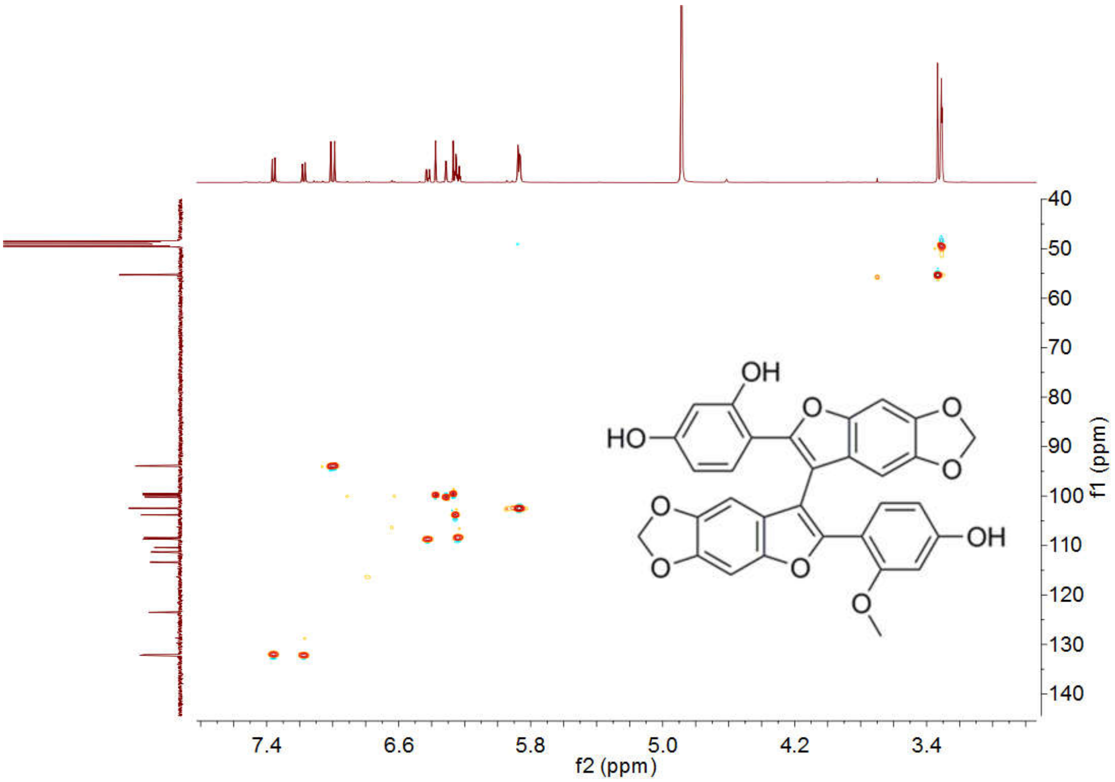

S43. HMBC spectrum of Shandougenine C (4)

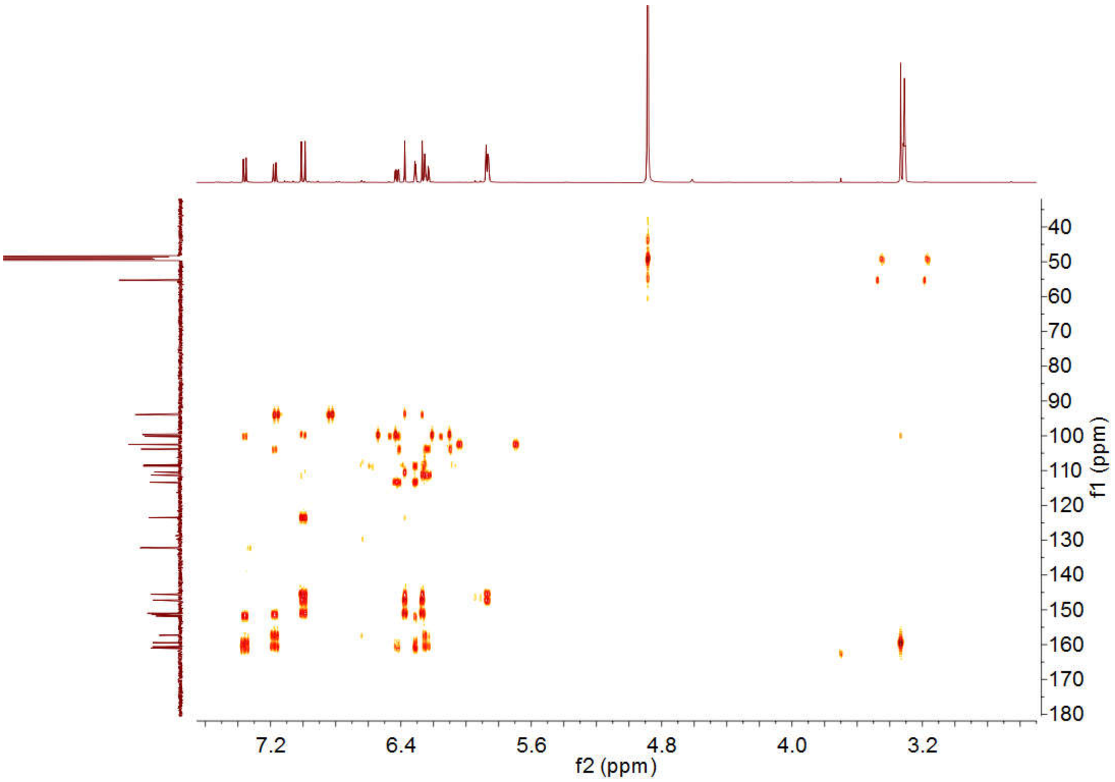

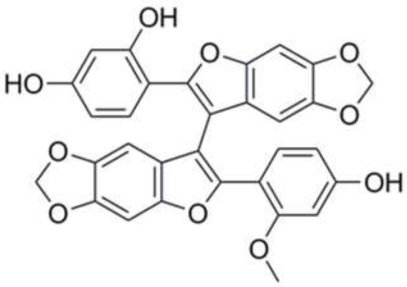

S44. ROESY spectrum of Shandougenine C (4)

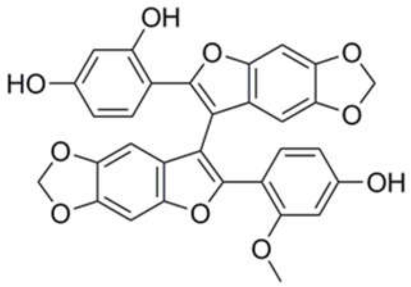

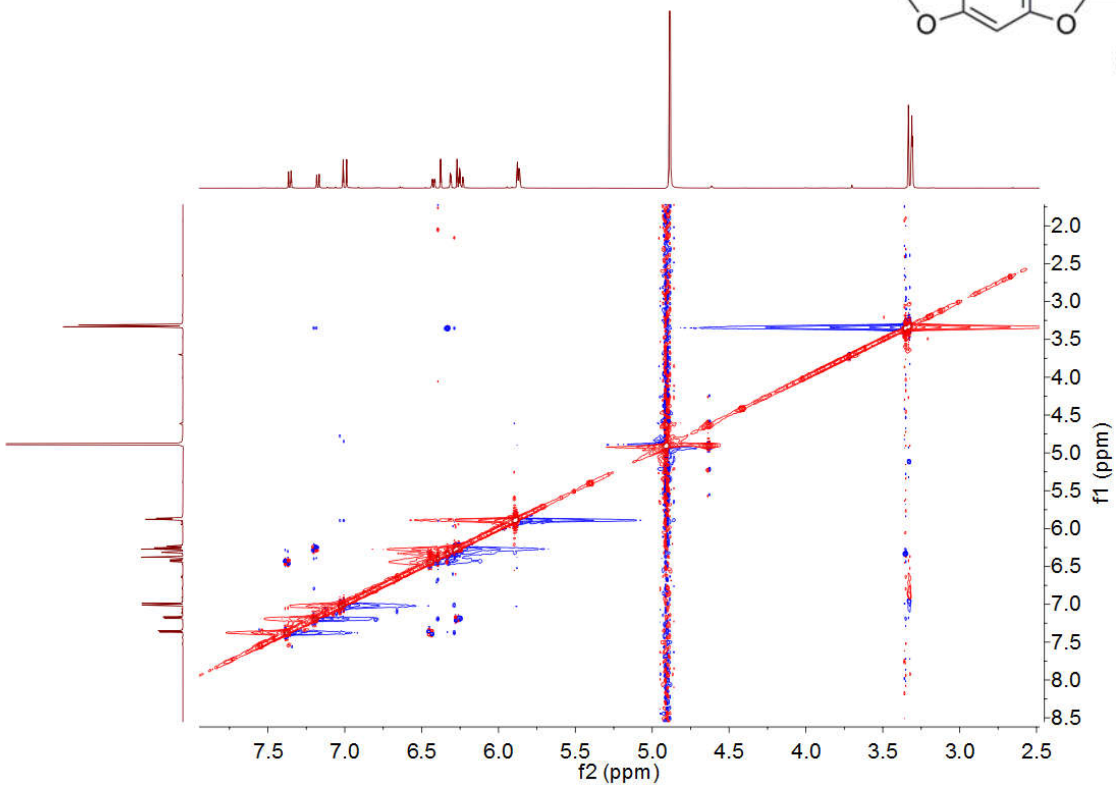

S45. The HPLC chromatograms of compounds 1-10

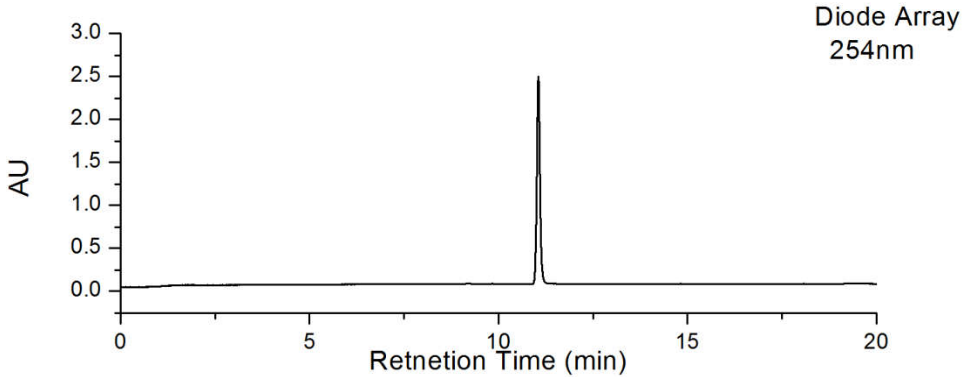

The HPLC chromatograms of compound **1**

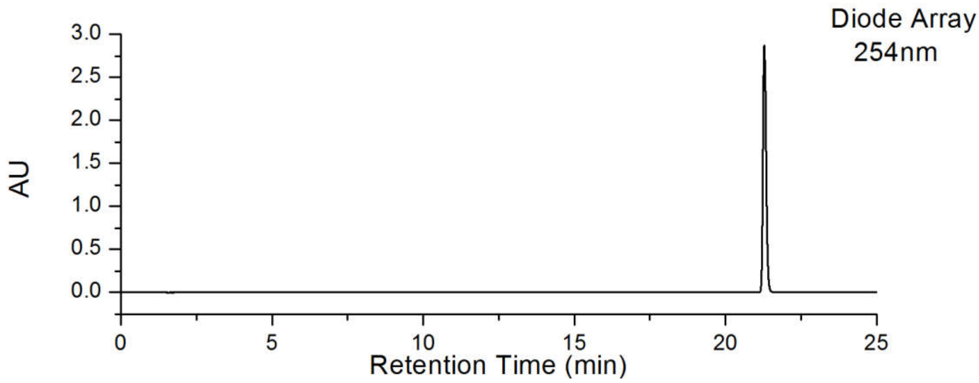

The HPLC chromatograms of compound **2**

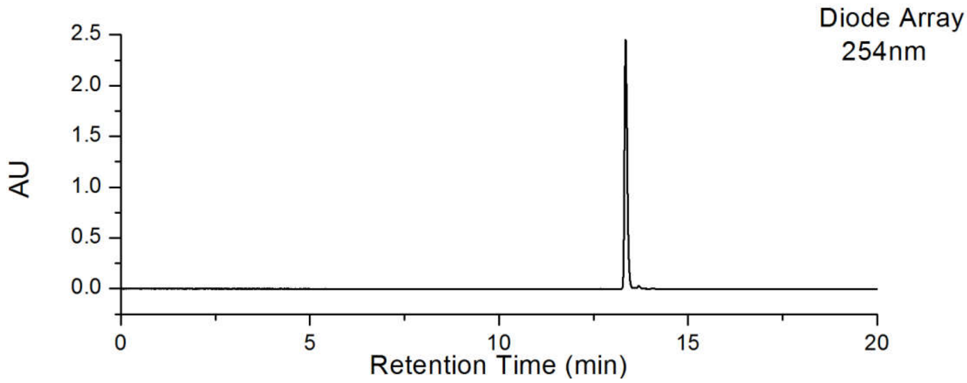

The HPLC chromatograms of compound **3**

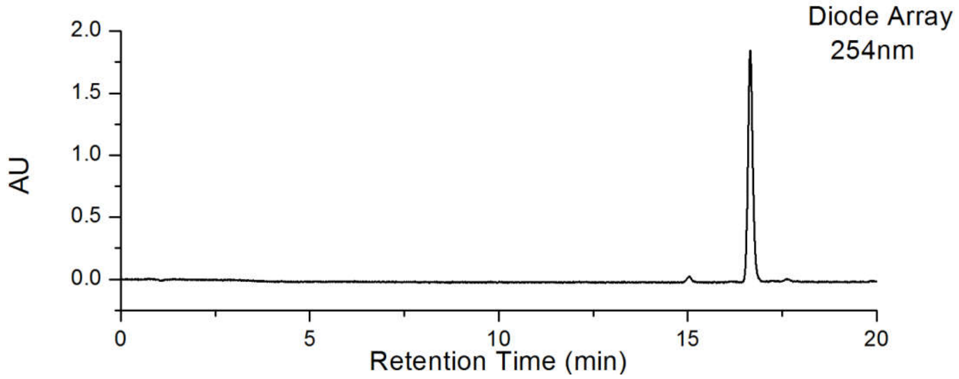

The HPLC chromatograms of compound **4**

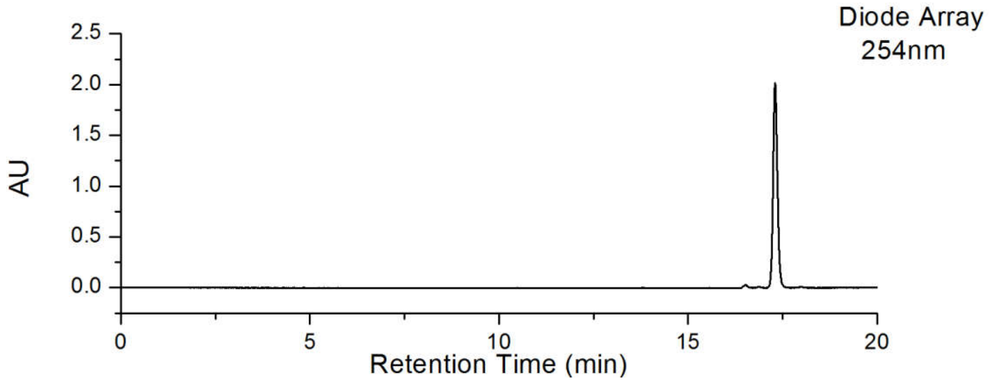

The HPLC chromatograms of compound **5**

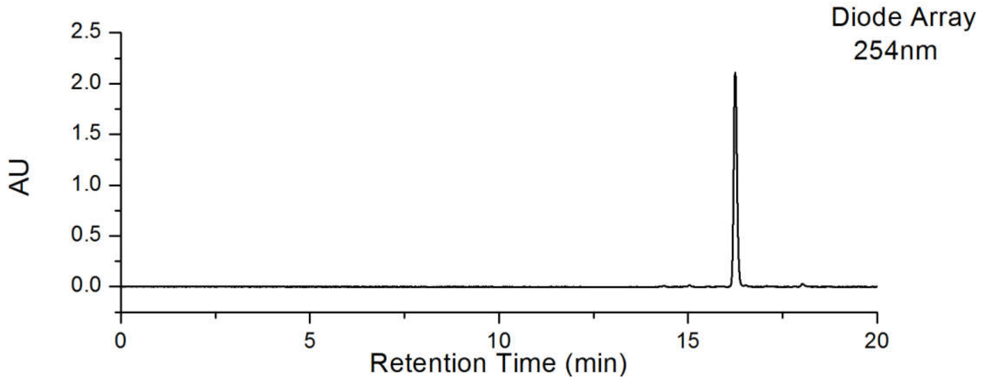

The HPLC chromatograms of compound **6**

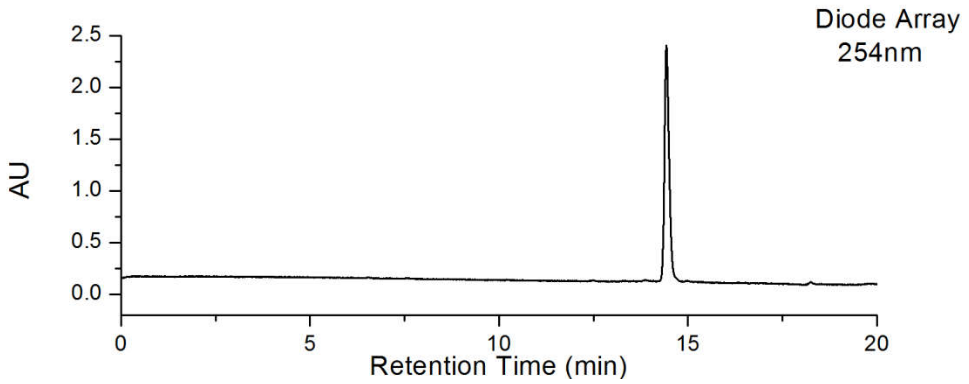

The HPLC chromatograms of compound **7**

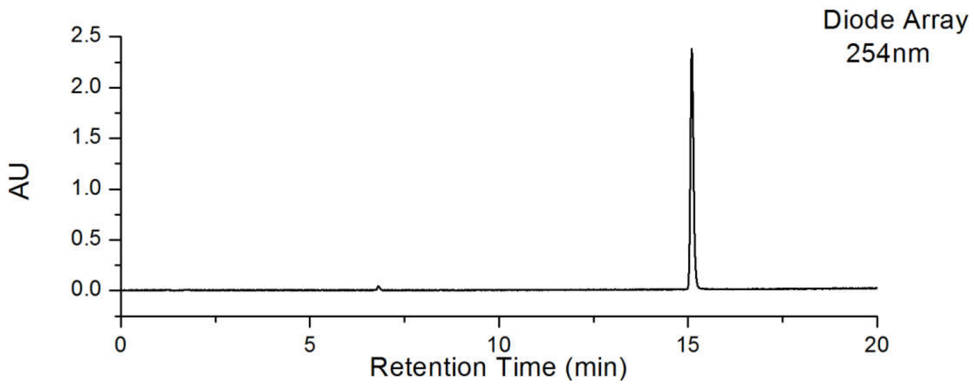

The HPLC chromatograms of compound **8**

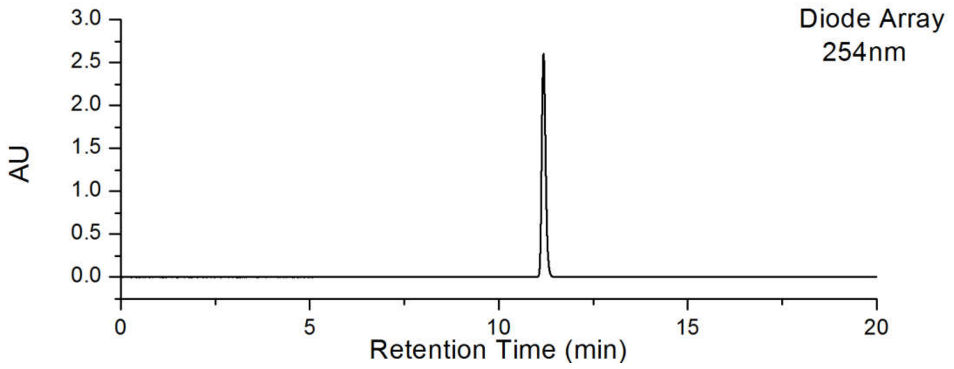

The HPLC chromatograms of compound **9**

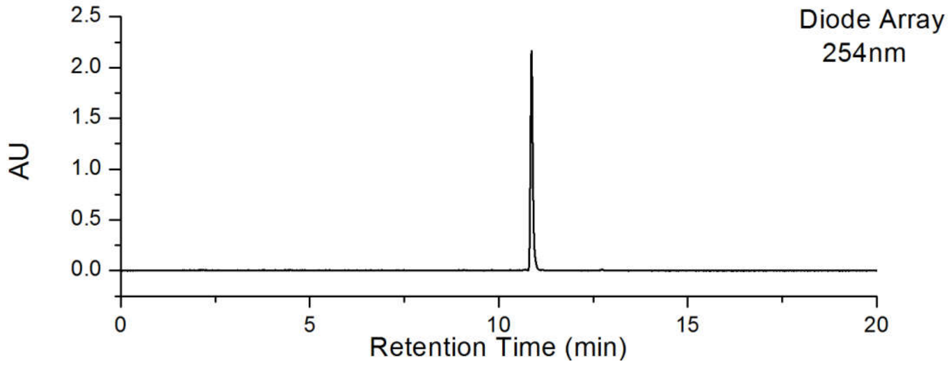

The HPLC chromatograms of compound **10**

S46. Screening methodology validation

There are several papers reported that IRAP-mOrange and GLUT4-eGFP could be applied to detect the GLUT4 translocation in L6 [1-3] and 3T3-L1 cells [4, 5]. In order to validate the feasibility of our IRAP translocation assay for discovering potential hypoglycemic agents, we have observed the effects when the GLUT4-eGFP or IRAP-marked L6 cells treated with insulin and berberine which are definitely pharmacodynamic GLUT4 agonists. L6 cells which stably express IRAP-mOrange and GLUT4-eGFP were cultured in α-MEM supplemented with 10% fetal bovine serum and 1% antibiotics (100 U/mL penicillin and 100 μg/mL streptomycin) at 37 ºC in 5% CO2. L6 cells was seeded in 48 well plates, and incubated until 100% confluence and then starved in serum-free MEM-α for 2 h. Afterwards, L6 cells were treated with insulin (10 nM) and berberine (5 μM). The cells were taken photos with a laser-scanning confocal microscope LSM 700 (Carl Zeiss, Jena, Germany) to supervise the IRAP-mOrange and GLUT4-eGFP translocation. And the images were captured with 555 nm excitation laser every 10 seconds in first 5 minutes and then every 5 minutes in later 30 minutes. The numerical aperture and object distance of the microscope were 1.3mm and 0.21mm. And magnification bar was 50μm.

During the experiment, as time went on, we could observe the green and red fluorescence enhanced significantly after treating with insulin and berberine in L6 cells (Figure S1). The results showed that GLUT4 and IRAP simultaneously

translocated onto the plasma membrane in 30 min when adding the GLUT4 agonist. GLUT4 has mainly been recruited to the PM throughout to the GLUTs storage vesicles (GSV). Three main proteins stored in GSV are GLUT4, IRAP, and Sortilin [6]. It was reported that IRAP and GLUT4 displayed a strong colocalization [7, 8] in many researches. Thus, detecting the IRAP can indirectly reflect the situation of GLUT4. So our results could be explained that detecting the IRAP-mOrange fluorescence could indirectly reflect the GLUT4 translocation. As the red fluorescence is more conspicuous than green fluorescence for observation, so we choose the IRAP-mOrange fluorescence assay for reflecting GLUT4 translocation.

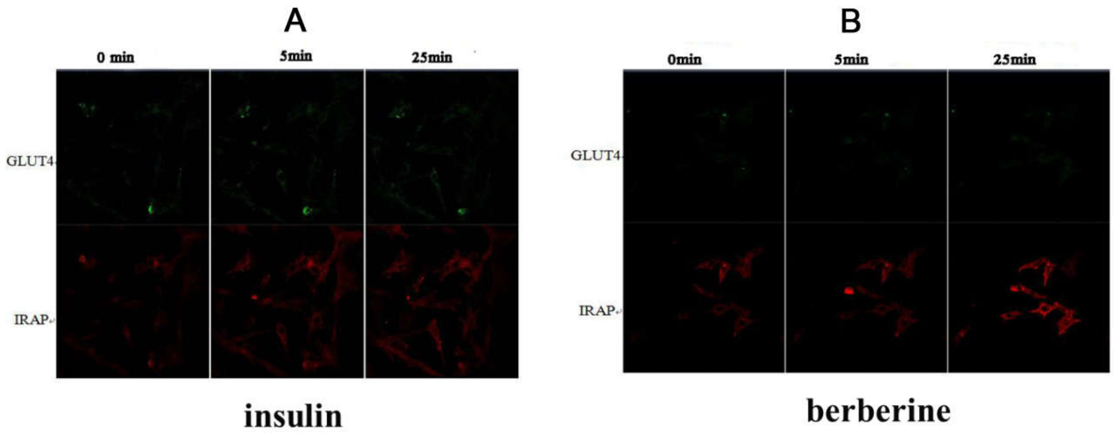

Figure S1. L6 cells were infected with IRAP-mOrange and GLUT4-eGFP in order to detect externalized GLUT4 translocation by confocal microscopy. (**A**) Confocal images in L6 cells incubated in the absence (0 min) or presence of insulin for 5min, 30 minutes. (**B**) Confocal images in L6 cells incubated in the absence (0 min) or presence of berberine for 5 min, 30 minutes.

Reference

1. X. Wang, F. Qu, Z. Chen, T. Liang and A. Qu, Labeling and imaging of GLUT4 in live L6 cells with quantum dots, *Biochem Cell Biol*, 2009, **87**, 687-694.
2. Q. Zhou, X. Yang, M. Xiong, X. Xu, L. Zhen, W. Chen, Y. Wang, J. Shen, P. Zhao and Q. H.

Liu, Chloroquine Increases Glucose Uptake via Enhancing GLUT4 Translocation and Fusion with the Plasma Membrane in L6 Cells, *Cell Physiol Biochem*, 2016, **38**, 2030-2040.

3. M. Huang, P. Zhao, M. Xiong, Q. Zhou, S. Zheng, X. Ma, C. Xu, J. Yang, X. Yang and T. C. Zhang, Antidiabetic activity of perylenequinonoid-rich extract from Shiraia bambusicola in KK-Ay mice with spontaneous type 2 diabetes mellitus, *J Ethnopharmacol*, 2016, **191**, 71-81.
4. L. Bai, Y. Wang, J. Fan, Y. Chen, W. Ji, A. Qu, P. Xu, D. E. James and T. Xu, Dissecting multiple steps of GLUT4 trafficking and identifying the sites of insulin action, *Cell Metab*, 2007, **5**, 47-57.
5. L. Jiang, J. Fan, L. Bai, Y. Wang, Y. Chen, L. Yang, L. Chen and T. Xu, Direct quantification of fusion rate reveals a distal role for AS160 in insulin-stimulated fusion of GLUT4 storage vesicles, *J Biol Chem*, 2008, **283**, 8508-8516.
6. J. Shi and K. V. Kandror, Sortilin is essential and sufficient for the formation of Glut4 storage vesicles in 3T3-L1 adipocytes, *Dev Cell*, 2005, **9**, 99-108.
7. A. Kumar, J. C. Lawrence, Jr., D. Y. Jung, H. J. Ko, S. R. Keller, J. K. Kim, M. A. Magnuson and T. E. Harris, Fat cell-specific ablation of rictor in mice impairs insulin-regulated fat cell and whole-body glucose and lipid metabolism, *Diabetes*, 2010, **59**, 1397-1406.
8. B. R. Rubin and J. S. Bogan, Intracellular retention and insulin-stimulated mobilization of GLUT4 glucose transporters, *Vitam Horm*, 2009, **80**, 155-192.
